# A general method for quantitative fractionation of mammalian cells

**DOI:** 10.1083/jcb.202209062

**Published:** 2023-03-15

**Authors:** Yael Udi, Wenzhu Zhang, Milana E. Stein, Inna Ricardo-Lax, Hilda A. Pasolli, Brian T. Chait, Michael P. Rout

**Affiliations:** 1 Laboratory of Cellular and Structural Biology, The Rockefeller University, New York, NY, USA; 2 Laboratory of Mass Spectrometry and Gaseous Ion Chemistry, The Rockefeller University, New York, NY, USA; 3 Laboratory of Virology and Infectious Disease, The Rockefeller University, New York, NY, USA; 4 Electron Microscopy Resource Center, The Rockefeller University, New York, NY, USA

## Abstract

Subcellular fractionation in combination with mass spectrometry–based proteomics is a powerful tool to study localization of key proteins in health and disease. Here we offered a reliable and rapid method for mammalian cell fractionation, tuned for such proteomic analyses. This method proves readily applicable to different cell lines in which all the cellular contents are accounted for, while maintaining nuclear and nuclear envelope integrity. We demonstrated the method’s utility by quantifying the effects of a nuclear export inhibitor on nucleoplasmic and cytoplasmic proteomes.

## Teaser

Udi et al. present a new method for quantitative subcellular fractionation of a wide range of mammalian cells while maintaining nuclear integrity. This method provides a fast and reliable means to perform mass spectrometry and DeDuvian enrichment analyses for the different cellular compartments.

## Introduction

The eukaryotic nucleus is surrounded by the nuclear envelope (NE) and contains most of the cell’s genetic material in the form of chromosomes (Clark et al., 2019; Webster et al., 2009). It represents the most prominent of several membrane-delimited organelles, each with its own specific and dynamic composition (Cohen et al., 2018; Cole, 2016; Schrader et al., 2015). Nucleocytoplasmic trafficking of macromolecules is a continuous highly regulated process occurring between the cytoplasm and the nucleus (Alberts et al., 2002; Christie et al., 2016; Macara, 2001; Silver, 1991; Wente and Rout, 2010; Yoneda, 1997). The correct nucleocytoplasmic localization of each macromolecule is a key for maintaining cell homeostasis (Bauer et al., 2015; Park et al., 2011).

The transport of molecules in and out of the nucleus is mediated by nuclear pore complexes (NPCs) embedded within the NE. A single NPC is composed of multiple copies of ∼30 different proteins, termed nucleoporins (Nups; Dultz et al., 2022; Stewart, 2022; Tingey et al., 2022; Wing et al., 2022), and is essential not only for nucleocytoplasmic trafficking but also for regulating genome organization and expression (Simon and Rout, 2014; Tingey et al., 2022). Abnormal nucleocytoplasmic localization of proteins has been linked to pathogenesis of many human diseases, such as cancer, metabolic, cardiovascular, and neurodegenerative diseases (Chung et al., 2018; Holmes et al., 2019; McLane and Corbett, 2009). More specifically, mislocalization of oncoproteins, tumor suppressors, and other cancer-related proteins, can interfere with normal cellular homeostasis and lead to tumor development and metastasis (Wang and Li, 2014). There are several mechanisms that may lead to protein mislocalization such as alteration of the trafficking machinery, altered protein targeting signals, and changes in protein modifications and interactions (Bauer et al., 2015; Hung and Link, 2011).

Given the importance of proper protein localization and its effect on pathological states, methods to fractionate mammalian cell lines for further biochemical studies into nuclear and cytoplasmic fractions are potentially of great utility. Ideally, such methods should be rapid, straightforward, reproducible, and be easily adaptable to multiple mammalian cell types, requiring only modest amounts of starting material. The method should ideally produce a manageable number of final fractions without loss-inducing wash steps. Each fraction should represent a single subcellular compartment (e.g., nuclei, NE) or sets of subcellular compartments (e.g., cytoplasmic membranes) and be recovered in high yield concentration and purity. For nuclear studies, the fractions should include morphologically intact nuclei and NEs, both to ensure retention of nuclear and NE proteomes and to allow ultrastructural analyses. Many published methods and commercially available kits lack some or all of these desirable characteristics, and thus are of particular but not general utility (Ogawa and Imamoto, 2021). For example, although the classic hepatocyte nuclear and nuclear envelope fractionations (Blobel and Potter, 1966; Kay et al., 1972) led to major discoveries concerning the organization of the nucleus, including detailed proteomic characterizations (Cronshaw et al., 2002; Wisniewski et al., 2016), they were largely restricted to one particularly favorable cell type. Here we presented a mammalian cell fractionation protocol that meets the aforementioned criteria. Our protocol yields three fractions: cytoplasm, cytoplasmic membranes (endoplasmic reticulum, Golgi, mitochondria, etc.), and nuclei. In turn, the nuclei fraction can be further fractionated into nucleoplasmic and NE fractions. As a proof of principle, we took advantage of this protocol’s proteomic suitability to demonstrate the effect of a known export factor inhibitor upon the nucleocytoplasmic distribution of cellular proteins, revealing how such inhibitors may preferentially affect cancer cells.

## Results

### An optimized subcellular fractionation protocol for mammalian cells

**Figure 1.**
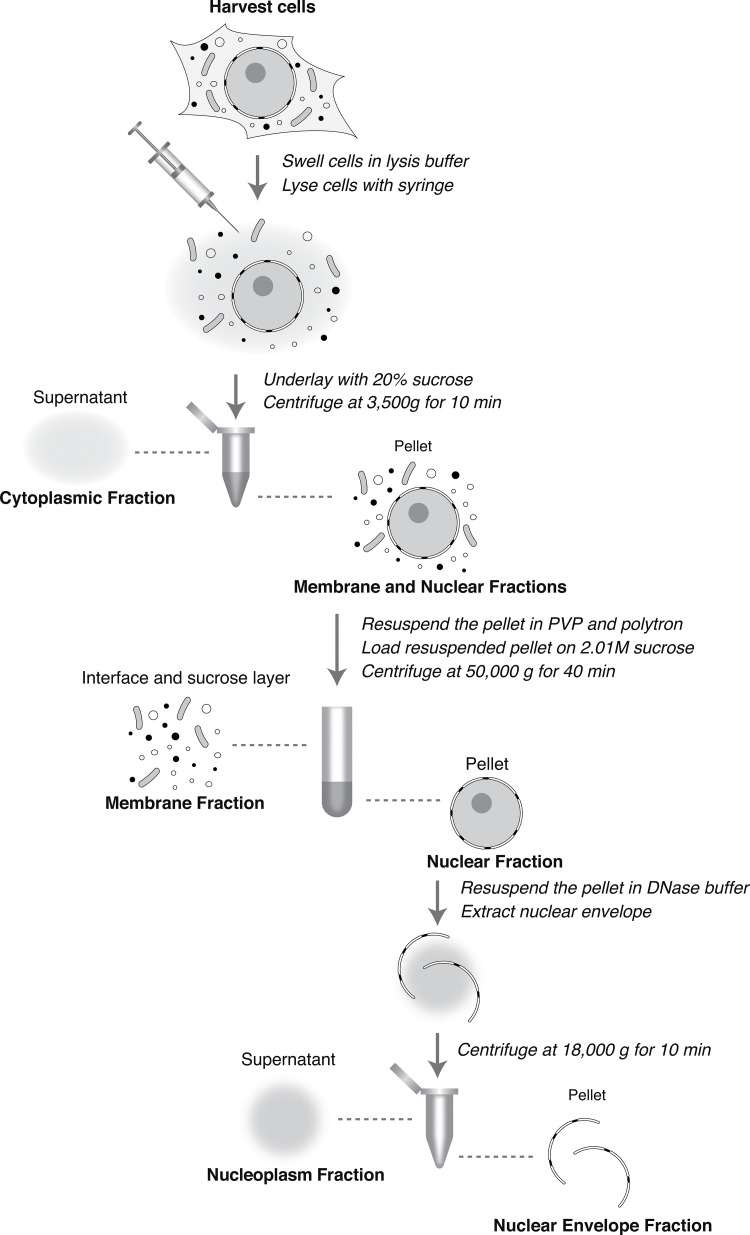
**Outline of the fractionation protocol.** The protocol steps are outlined, employing cell lysis, and several centrifugation steps. Cells were fractionated into four distinct fractions: cytoplasm, membrane, NE, and nucleoplasm.

In order to develop a reliable and reproducible fractionation protocol for mammalian cells, we started by modifying a method that has been successfully applied to the quantitative fractionation and proteomic analysis of *Saccharomyces cerevisiae* and *Trypanosoma brucei* cells, using the stabilizing agent polyvinylpyrrolidone (PVP) in our lysis buffer (Cronshaw et al., 2002; DeGrasse et al., 2008; Matunis, 2006; Niepel et al., 2017 *Preprint*; Obado et al., 2016; Rout and Field, 2001; Strambio-de-Castillia et al., 1995). This is a well-characterized polymer, and it is used in many medical and technical applications (Koczkur et al., 2015; Kurakula and Rao, 2020). PVP is known to stabilize nuclei against disintegration (Niepel et al., 2017 *Preprint*), although, due to the difference in characteristics between mammalian cells, *S. cerevisiae* and *T. brucei* a significant redesign of these protocols for the subcellular fractionation of mammalian cells was needed (Fig. 1). It is rapid, straightforward, reproducible, and generates a minimum number of discrete and defined fractions recovered in high yield, concentration, and enrichment, including morphologically intact nuclei and NEs (below). Two further advantages of this protocol are the small amounts of cells needed and its adaptivity to a variety of cell lines by adjusting three simple parameters during cell lysis (lysis buffer volume, permeabilizing detergent concentration, and degree of cell shear; see Materials and methods). Briefly, cells are harvested and washed once with ice cold PBS. Next, the cells are briefly allowed to swell on ice in the lysis buffer. Depending on the specific cell line, the ratio of lysis buffer to cell slurry can be adjusted between 8:1 and 5:1 with the permeabilizing non-ionic detergent percentage in the lysis buffer being varied between 0.015 and 0.045%—amounts low enough to avoid membrane solubilization while promoting cell lysis. Once the cells are swollen, they are lysed through gentle shearing with a syringe and needle. The progress of cell lysis is monitored by phase contrast microscopy to ensure both sufficient lysis and dispersal of cytoplasmic material away from the nuclei. After this step, the lysed cells are underlaid with 20% sucrose in a PVP-containing buffer and centrifuged. The resulting supernatant represents the cytoplasmic fraction, and the pellet contains the membranous material and nuclei. The pellet is then resuspended in a PVP-containing buffer and further homogenized by a polytron to separate any membranous non-NE material from the nuclei. The resulting suspension is then overlaid on 2.01 M Sucrose in a PVP-containing buffer and subjected to ultracentrifugation, after which the interphase between the buffer and the sucrose layer contains the “membrane” fraction (comprising cytoplasmic membranes and organelles plus other larger cytoplasmic materials) with the nuclei pelleting at the bottom of the tube. At this stage, there are three fractions: cytoplasm, membranes, and nuclei. The nuclei can be further fractionated into nucleoplasmic and NE fractions through resuspension in a DNAse I-containing buffer and centrifugation to separate the released NEs from the resulting nucleoplasmic suspension. The entire protocol requires ∼3 h.

**Figure S1.**
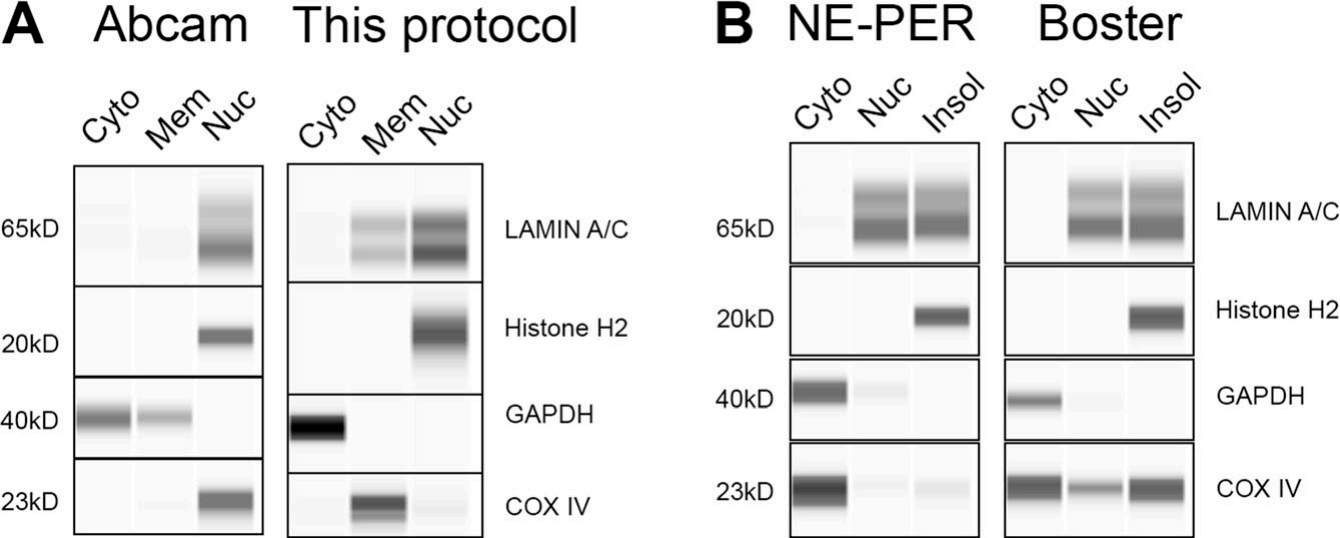
**Wes protein analysis of HeLa cells fractionated using commercial kits. (A)** The fractions were blotted for the relevant cellular markers using the Wes ProteinSimlple capillary system. Abcam cell fractionation kit compared with our protocol (duplicate for Fig. 2 B HeLa cell line). **(B)** NE-PER and Boster extraction kits.

In order to test the advantage of this protocol over the commercially available kits, we have employed three different commercially available extraction/fractionation kits to fractionate HeLa cells: #78833, Thermo Fisher Scientific; #AR0106, Boster; and #ab109719, Abcam (Fig. S1). The main disadvantage of the commercial kits is the unknown detergent or detergents used and their concentration. For example, the kit #ab109719, Abcam provides two detergents, “detergent I” and “detergent II,” that are added to the lysis buffers on different steps during the protocol. The composition and concentration of the detergent are required for any further downstream mass spectrometry analysis. Furthermore, the enrichment of the different fractions is inferior to the enrichments we presented (Fig. S1). Lastly, in some commercial extraction kits, there is an insoluble nuclear fraction that is discarded; however, as can be seen in Fig. S1, there is a considerable amount of proteins of interest in this fraction, and so using these kits for downstream quantitation will result in imprecise depiction of the studied system.

### The subcellular fractionation protocol can be optimized for various mammalian cell lines

**Figure 2.**
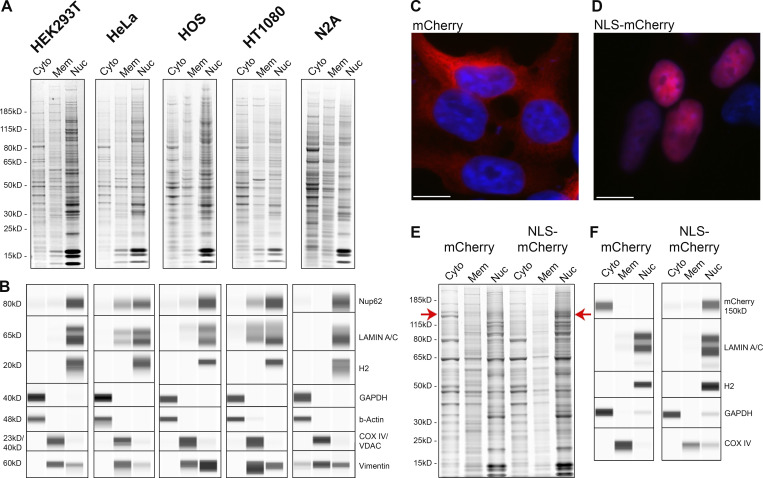
**Sub-cellular fractions from five different cell lines and nuclear intactness. (A)** SDS-PAGE profile of the proteins in the subcellular fractions from five different cell lines obtained during fractionation described in Fig. 1. Gel was stained with AquaStain. **(B)** Wes protein analysis. The fractions were blotted for the relevant cellular markers using the Wes ProteinSimple capillary system. **(C and D)** Fluorescence images of HEK293T cells transfected with mCherry-LacZ (C) Or with NLS-mCherry-LacZ construct (D). Cell nuclei were stained with DAPI. Fluorescent images were obtained with the Revolve R4, Model: RVL2-K2 with Olympus 60× Plan Fluorite Oil IRIS Phase 3 objective with an NA of 1.25 at room temperature. Images were acquired with Echo Pro version 6.4.1 and processed with Photoshop. Scale bar, 10 μm. **(E)** SDS-PAGE profile of the proteins in the subcellular fractions of HEK293T cells transfected with mCherry-LacZ and NLS-mCherry-LacZ. Red arrows indicate the mCherry-LacZ band on the gel. Gel was stained with AquaStain. **(F)** Wes protein analysis. The fractions were blotted for the relevant cellular markers and mCherry using the Wes ProteinSimple capillary system. All the samples for SDS-PAGE and Wes were loaded at an equal total protein concentration of 0.5 mg/ml. Source data are available for this figure: SourceData F2.

We tested our protocol on five different cell lines, derived from different tissues: HEK293T (derived from human embryonic kidney cells), HeLa (human cervical cancer cells), HOS (human osteosarcoma cells), HT1080 (human fibrosarcoma cells), and N2A (mouse neuroblastoma cells). The protocol was slightly adjusted for each cell line based on the three parameters mentioned above. Each fraction was analyzed by SDS-PAGE and Coomassie blue staining (Fig. 2 A). Notably, the histones protein banding pattern is highly enriched in the nuclear fractions of all the cell lines. The different fractions were also immunoblotted for subcellular markers (Fig. 2 B), and all cell lines demonstrate extremely high enrichment of each marker in its corresponding fraction; thus, the cytoplasmic protein GAPDH and cytoskeletal protein β-actin are almost exclusively in the cytoplasmic fraction, the membrane fractions are highly enriched for the mitochondrial markers (CoxIV or VDAC) and vimentin, and the nuclear marker Histone H2A.Z is almost exclusively in the nuclear fractions. Hence, each one of the fractions presented here is highly enriched with the appropriate markers (see below Fig. 4). In our protocol, we found that vimentin is highly enriched in the membrane fraction rather than in the cytoplasmic fraction. This phenomenon can be explained by the perinuclear cage structure formed by vimentin filaments, likely extracted relatively intact and so co-sedimenting with other larger cellular membranous structures (Patteson et al., 2019), and in addition, vimentin has been previously reported to associate with different cellular organelles (mitochondria, Golgi apparatus, endosomes, and lysosomes); hence, its enrichment in the membrane fraction is expected (Gao and Sztul, 2001; Hartig et al., 1998; Styers et al., 2004; Tang et al., 2008). Lamin A/C was also detected to some extent in the membrane fraction. Importantly, detectable amount of lamin in the membrane fraction does not appear to indicate nuclear disruption, since no histones were detected in the same fraction. The presence of lamin A/C in this fraction can be attributed at least in part to the lamin A/C released from the NE during mitosis (Dittmer and Misteli, 2011). The differences between the different cell lines in lamin levels present in the membrane fraction is probably a result of the different expression levels of lamin in different cell lines, as it has been previously reported that lamin A/C expression is higher in HeLa cells compared with HEK293 cells (Piekarowicz et al., 2017). Finally, small amounts of Nup62 were detected in the membrane fraction as well. These are attributed to the annulate lamellae (AL) organelles present in the cytoplasm. AL are membrane sheets embedded with pore complexes (ALPC) continuous with the membrane network of the ER (Hampoelz and Baumbach, 2023; Raghunayakula et al., 2015; Ren et al., 2019). The immunoblots for GAPDH, actin, histones, vimentin, and membrane markers were quantified (see Materials and methods) with ImageJ software (Table 1). Quantification of the immunoblots further supports the high enrichment of the different fractions.

**Table 1.** Wes quantification of the cellular markers for the different cell lines

		Cyt%	Memb%	Nucl%
HEK293T	GAPDH	99.08	0.00	0.92
	Actin	99.84	0.00	0.16
	COX IV	0.00	99.67	0.33
	Vimentin	0.00	83.37	16.63
	H2	0.00	0.00	100.00
HeLa	GAPDH	100.00	0.00	0.00
	Actin	100.00	0.00	0.00
	COX IV	0.82	98.70	0.47
	Vimentin	0.00	97.51	2.49
	H2	0.00	0.00	100.00
HOS	GAPDH	100.00	0.00	0.00
	Actin	100.00	0.00	0.00
	COX IV	0.80	97.90	1.30
	Vimentin	0.00	64.98	35.02
	H2	0.00	0.00	100.00
HT1080	GAPDH	100.00	0.00	0.00
	Actin	97.10	2.90	0.00
	COX IV	3.59	95.95	0.46
	Vimentin	0.00	88.40	11.60
	H2	0.00	0.00	100.00
N2A	GAPDH	91.10	0.00	8.90
	Actin	98.34	1.54	0.12
	VDAC	0.00	98.05	1.95
	Vimentin	3.71	88.76	7.53
	H2	0.00	0.00	100.00

### The integrity of the nuclei is maintained during fractionation

**Figure S2.**
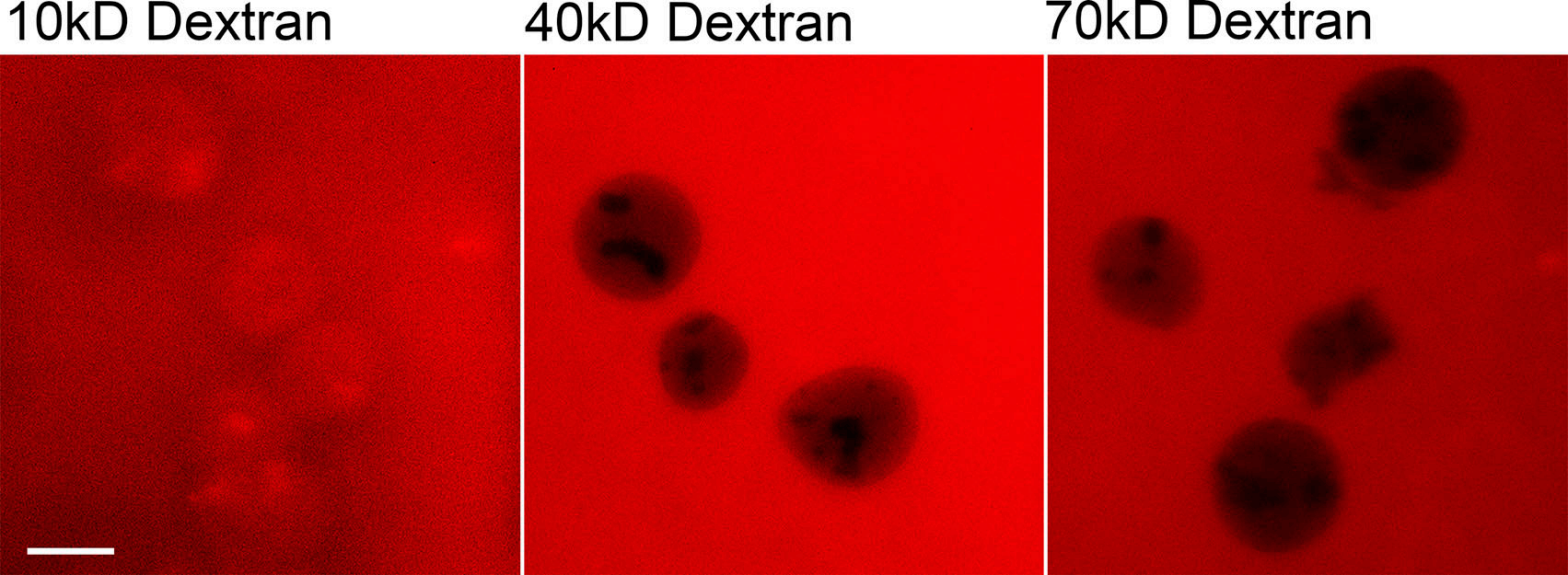
**Nuclear permeability assay.** HEK293T nuclei were stained with 10 kD, 40 kD, and 70 kD Tetramethylrhodamine Dextran. Scale bar, 10 μm.

One of the major challenges in cellular fractionation is maintaining the integrity of the nuclei, avoiding leakage of native nucleoplasmic assemblies (Ogawa and Imamoto, 2021). In order to test whether nuclear integrity is indeed maintained, we transiently transfected HEK293T cells with two mCherry reporters, one of which carries a nuclear localization signal to drive the reporter into the nucleoplasm while the other lacks the signal and so will remain in the cytoplasm. Both were incorporated into a LacZ fusion to prevent passive diffusion via the NPC that occurs with much smaller exogenous constructs (Kane et al., 2018; Wuhr et al., 2015; Fig. 2, C and D). We fractionated these cells, and the fractions were analyzed by SDS-PAGE (Fig. 2 E). Strikingly, even by Coomassie blue staining, the bands corresponding to the reporters being restricted to their appropriate fractions can be identified (Fig. 2 E, red arrow). The high degree of differential partitioning was confirmed by immunoblotting for mCherry in addition to the standard markers (Fig. 2 F). This result confirms the general integrity of the nuclei, without significant leakage of nucleoplasmic materials (or of cytoplasmic materials into the nucleus), after fractionation. To further verify the integrity of the fractionated nuclei, we tested the permeability of the isolated nuclei with fluorescently labeled Dextran in a range of molecular weights (10–70 kD). As expected (Ferrando-May et al., 2001; Raices and D’Angelo, 2022), the isolated nuclei showed high permeability of the 10 kD Dextran, very little permeability of the 40 kD, and little to no permeability of the 70 kD Dextran (Fig. S2). This result further supports that the NPC and the NE are kept intact during our fractionation protocol, and importantly, as the great majority of cellular proteins are in large assemblies (Wuhr et al., 2015) loss of active transport will not lead to their nucleocytoplasmic redistribution as these assemblies are too large to passively diffuse across the NPC.

### Nuclear envelopes can be efficiently segregated from nucleoplasm

**Figure 3.**
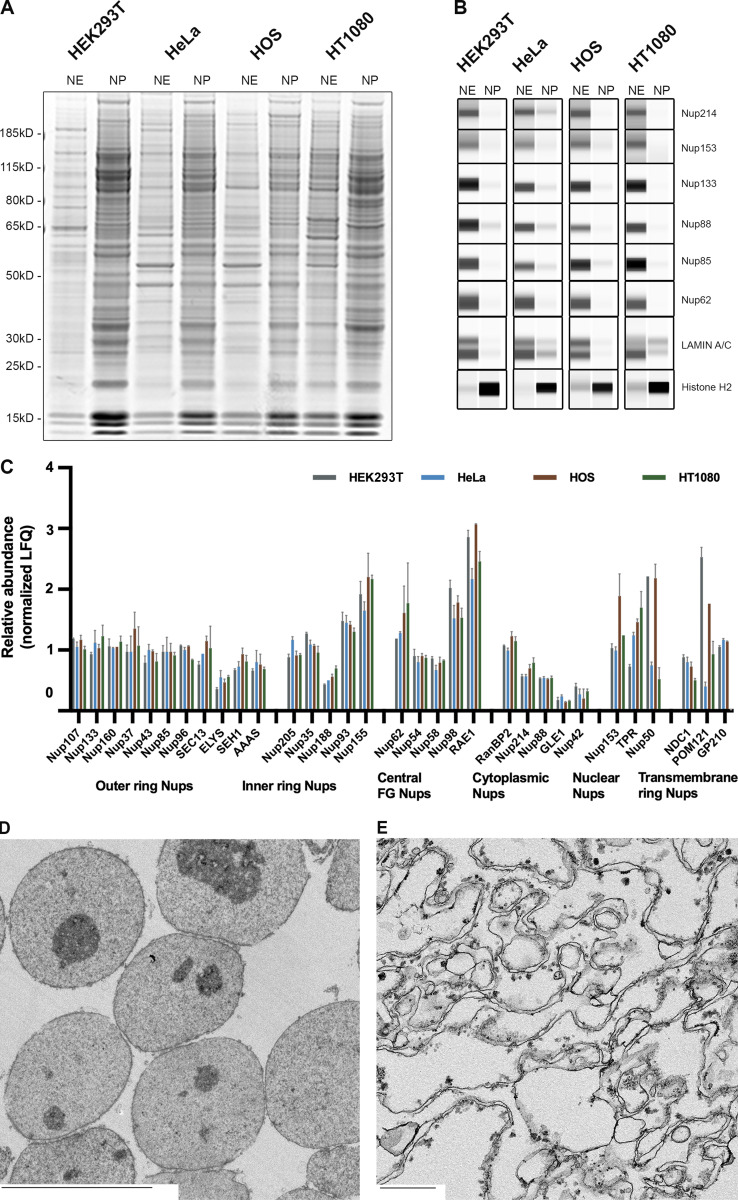
**NE segregation from the nucleoplasm. (A)** SDS-PAGE profile of the proteins in the NE and the nucleoplasm (NP) from different cell lines. Gel was stained with AquaStain. **(B)** Wes protein analysis of the NE and NP fractions. The fractions were blotted for different NPC proteins, Histone, and Lamin A/C. **(C)** Label-free MS analysis of the NE fractions from different cell lines. Proteins are organized according to their localization within the NPC. Error bars represent SD calculated using Microsoft Excel. **(D)** EM image of the nuclear fraction of HEK293T cells. Scale bar, 10 μm **(E)** EM image of the NE fraction of HEK293T cells. Scale bar, 600 nm. Source data are available for this figure: SourceData F3.

**Figure S3.**
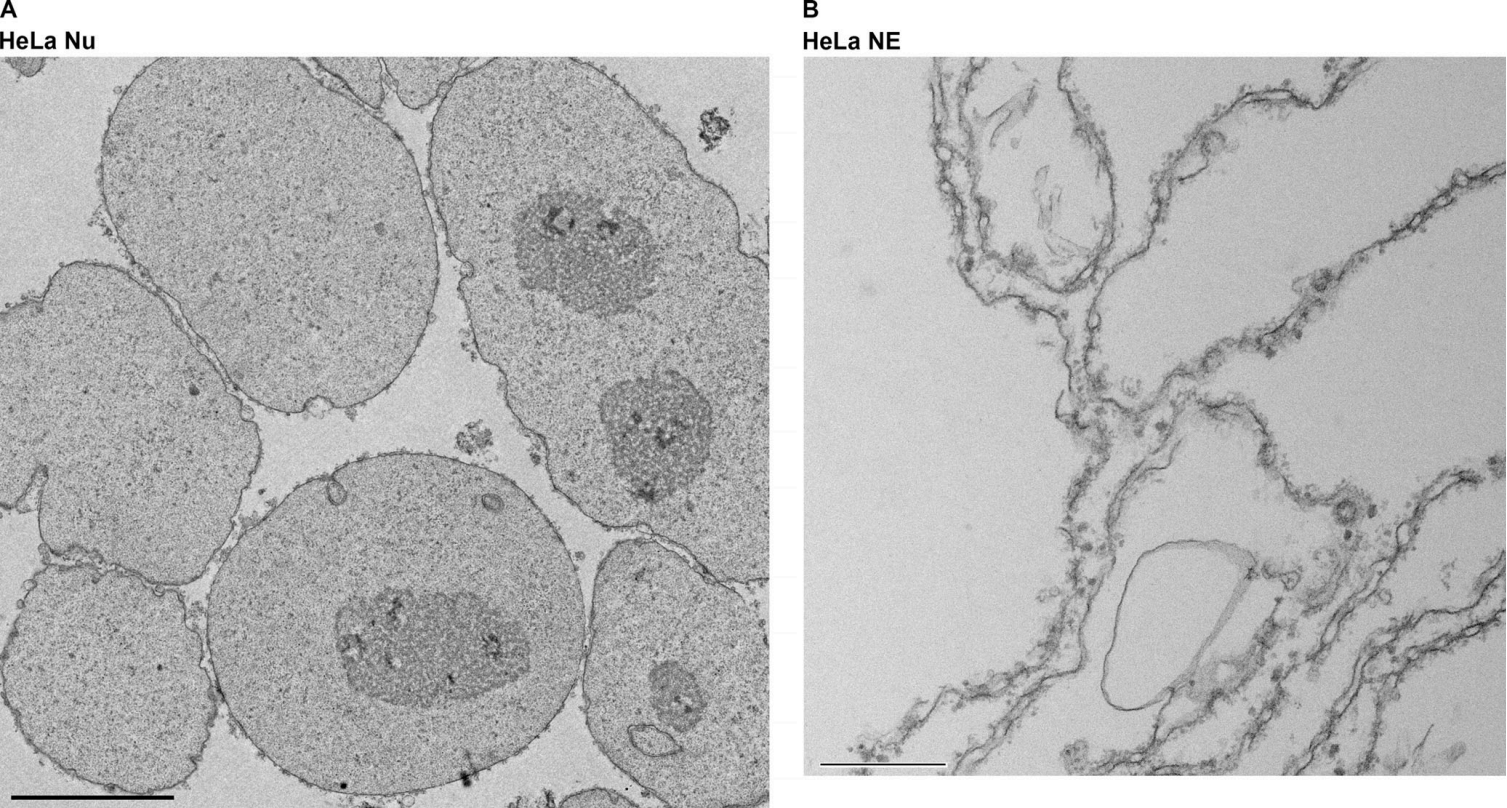
**Left: EM image of the nuclear fraction of HeLa cells.** Scale bar, 4 μm. Right: EM image of the NE fraction of HeLa cells. Scale bar, 500 nm.

We next used a one-step, high-yield approach to release the NEs by combining DNase, RNase, and Heparin (Cronshaw et al., 2002) to minimize the number of fractions to two, while still producing NEs of acceptable enrichment and high morphological intactness. Notably, this step can be tuned by varying the heparin concentration in order to increase the degree of peripheral chromatin extraction from the NEs; as this chromatin is specifically associated, some applications may wish to be more conservative in its removal (as we were here). The resulting NEs were analyzed by SDS-PAGE (Fig. 3 A). A panel of commercially available anti-Nup antibodies was used in order to immunoblot for their abundance between the two fractions. As expected, all the Nups we tested were found in the NE fraction for all the cell lines (Fig. 3 B). We also similarly examined the distribution of lamin A/C and histone H2 between the two fractions, and again, as expected, the lamin signal was predominantly in the NE fraction, whereas the histone signal was predominantly in the nucleoplasmic fraction (Table 2). To complement our biochemical assays, we used quantitative label-free mass spectrometry (MS) to further characterize the NE fraction of the different mammalian cell lines. All mammalian NPC components were identified for each of the cell lines tested. Notably, the stoichiometric ratios of the different NPC components were in good agreement with previously published data (Fig. 3 C; Ori et al., 2013). To further assess the morphological integrity of both the nuclei and the NEs, we obtained transmission electron microscopy images of thin section embedded samples of these fractions (Fig. 3, D and E and Fig. S3), confirming their morphological integrity and purity.

**Table 2.** Wes quantification of the Histone H2.A signal in the NE and nucleoplasm fractions

	NE %	Nucleoplasm %
HEK293T	2.88	97.12
HeLa	3.46	96.54
HOS	13.07	86.93
HT1080	9.01	90.99

### Proteomic assessment of the method

**Figure 4.**
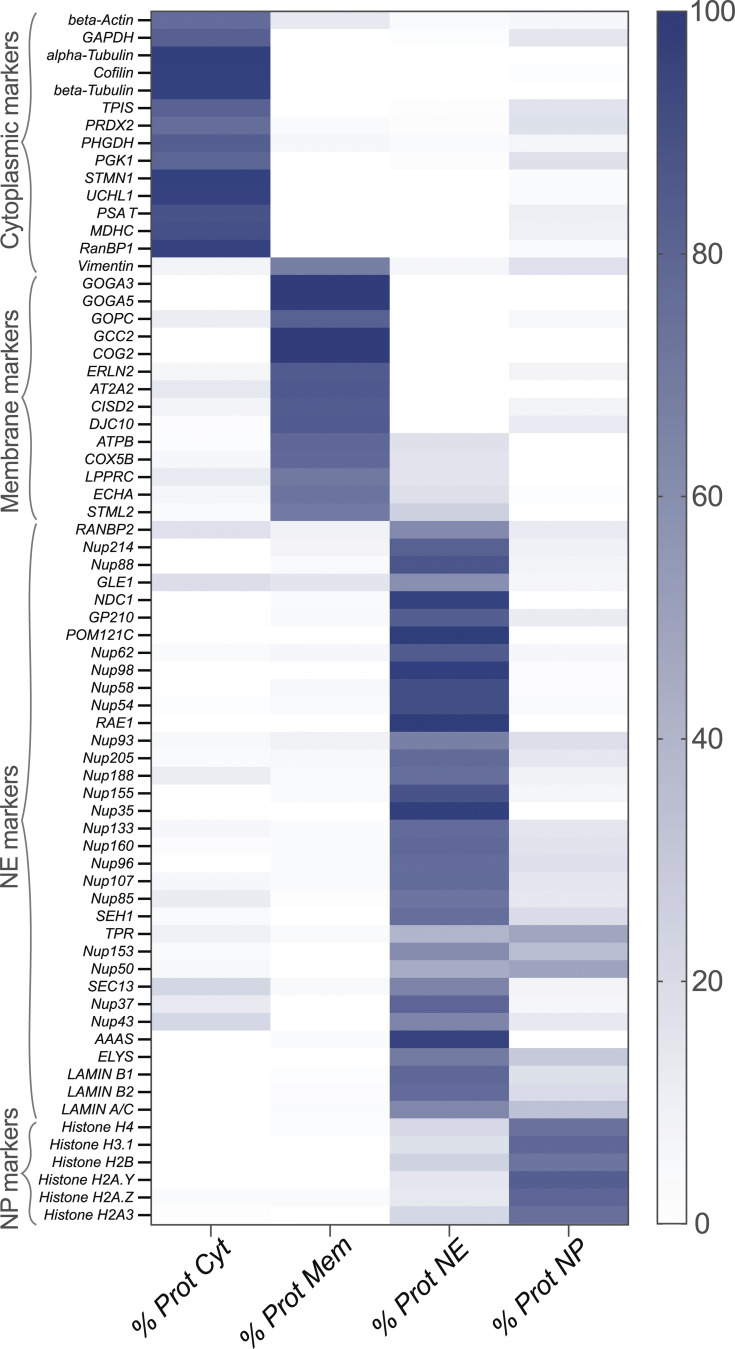
**Heatmap analysis of the label free MS of the different fractions.** Selected markers from each cellular compartment are presented. Heatmap values were calculated as described in Materials and methods.

**Figure S4.**
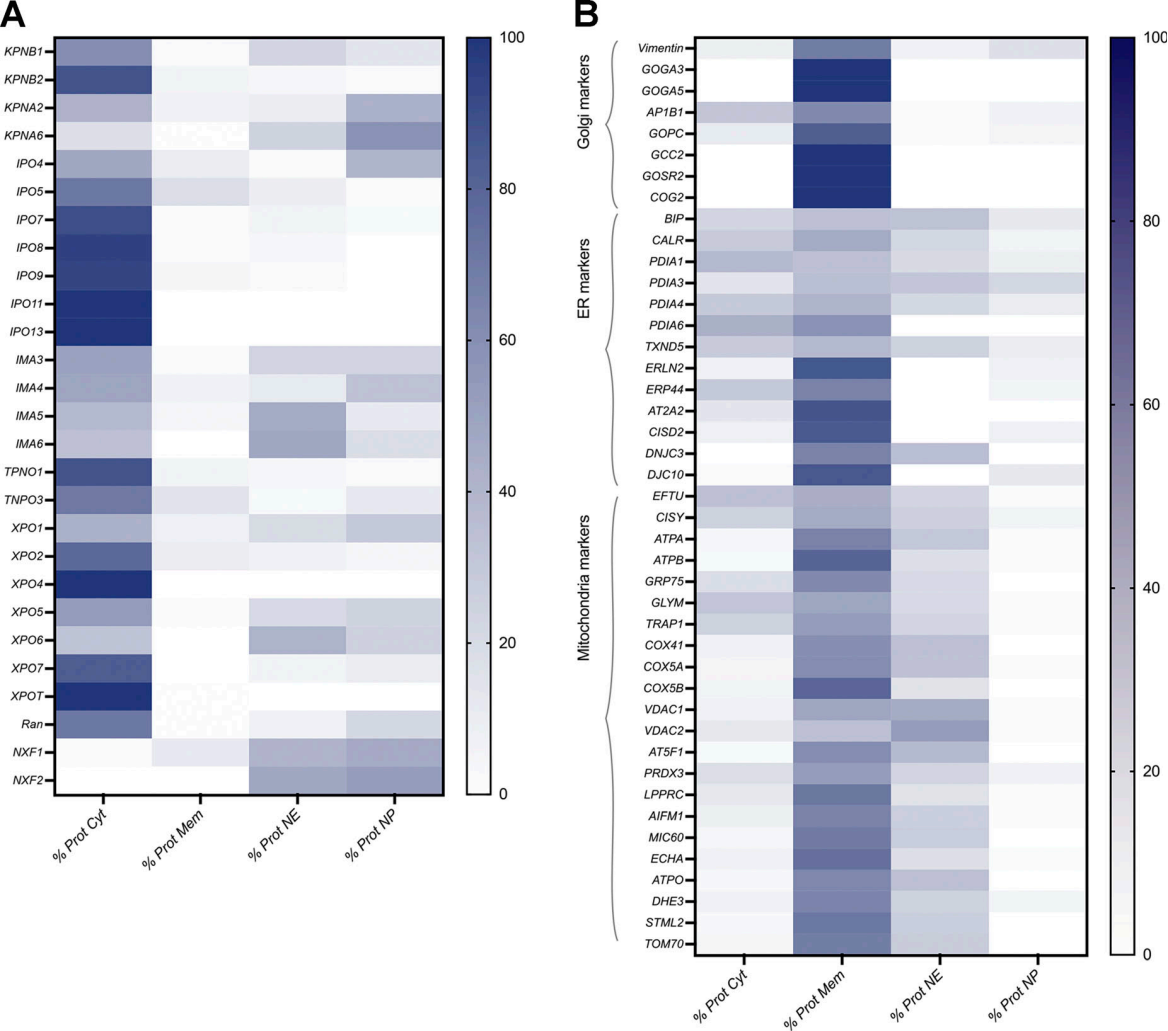
**Heatmap analysis of the label-free MS of the different fractions. (A)** Transport factors. **(B)** Membrane markers.

To further assess the enrichment of each fraction, we performed quantitative label-free MS analyses of the cytoplasmic, membrane, NE, and nucleoplasmic fractions of HEK293T cells, providing a measure of the degree to which each identified protein partitions between these four fractions. Fig. 4 provides a heatmap of the relative abundance of proteins that are considered as markers of the relevant fractions. Notably, all the cytoplasmic markers are found predominantly in the cytoplasmic fraction, the abundance percentage of these proteins all being more that 90% in the cytoplasmic fraction (see Table S1). NPC proteins and lamins serve here as markers for the NE fraction, and these proteins are highly enriched in this fraction. NPC proteins are also found in the membrane fraction as mentioned above. All other known NE-associated proteins are similarly highly enriched in the NE fraction although the nuclear basket proteins, Tpr, Nup153, and Nup50, also have a significant presence in the nucleoplasm. Nup153 and Nup50 are known to exchange relatively rapidly with a nucleoplasmic pool (Rabut et al., 2004), and it was also previously reported that Tpr is localized in discrete intranuclear foci in addition to its NE localization (Frosst et al., 2002). Overall, the analysis confirms the high degree of segregation and enrichment of these fractions. Likewise, the histones as chromatin markers are largely nucleoplasmic, but as expected, a significant fraction is associated with the NE as peripheral chromatin. See Fig. S4 and Tables S2 and S3 for a heatmap of the transport factors (Fig. S4 A and Table S2) and additional membrane markers (Fig. S4 B and Table S3).

### Proteomic analysis of Crm1-mediated nuclear export

**Figure S5.**
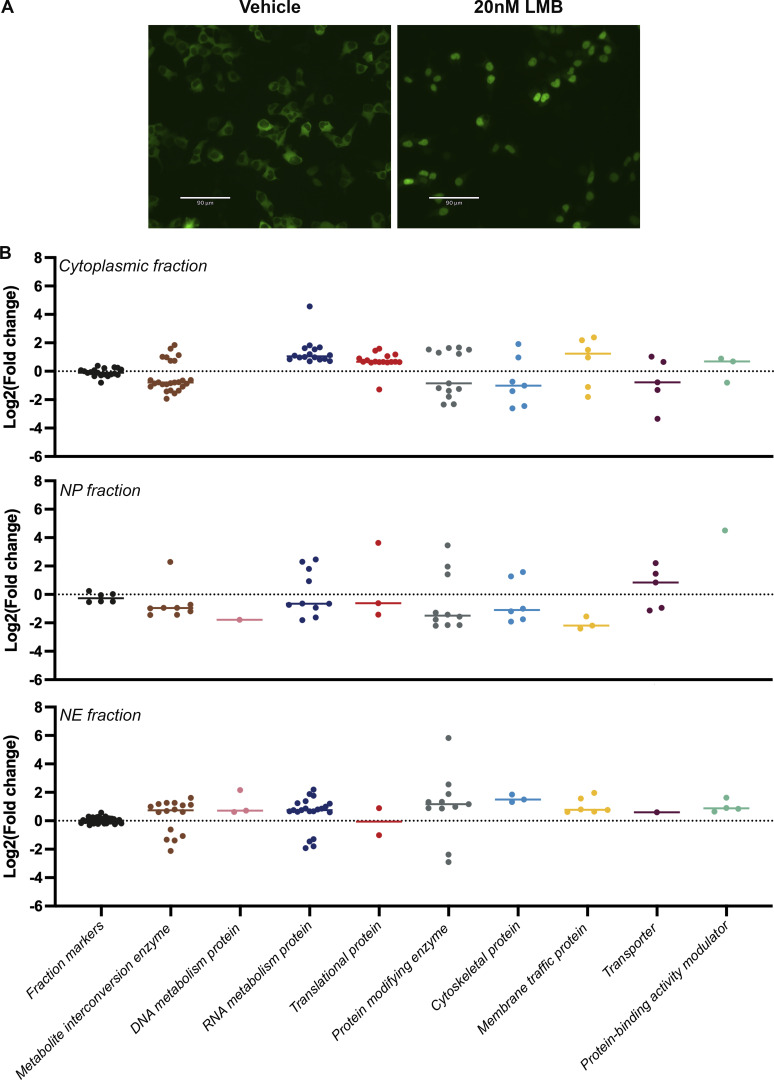
**MS analysis of Crm1 inhibition in HEK293T cells. (A)** Fluorescence images of HEK293T cells stably expressing GFP_2_-NLS-NES treated with vehicle or 20 nM LMB. Fluorescent images were obtained from live cells in DMEM at room temperature with the Revolve R4, Model: RVL2-K2 with Olympus 40× Plan Fluorite Phase Ph2 NA: 0.75. Images were acquired with Echo Pro version 6.4.1. and processed with Photoshop. **(B)** Proteins with most significantly affected cellular distribution (fold change >1.5 and P value <0.05) after LMB treatment are presented in a scatter plot. Proteins are categorized according to protein classes using the PANTHER GeneOntology server.

**Figure 5.**
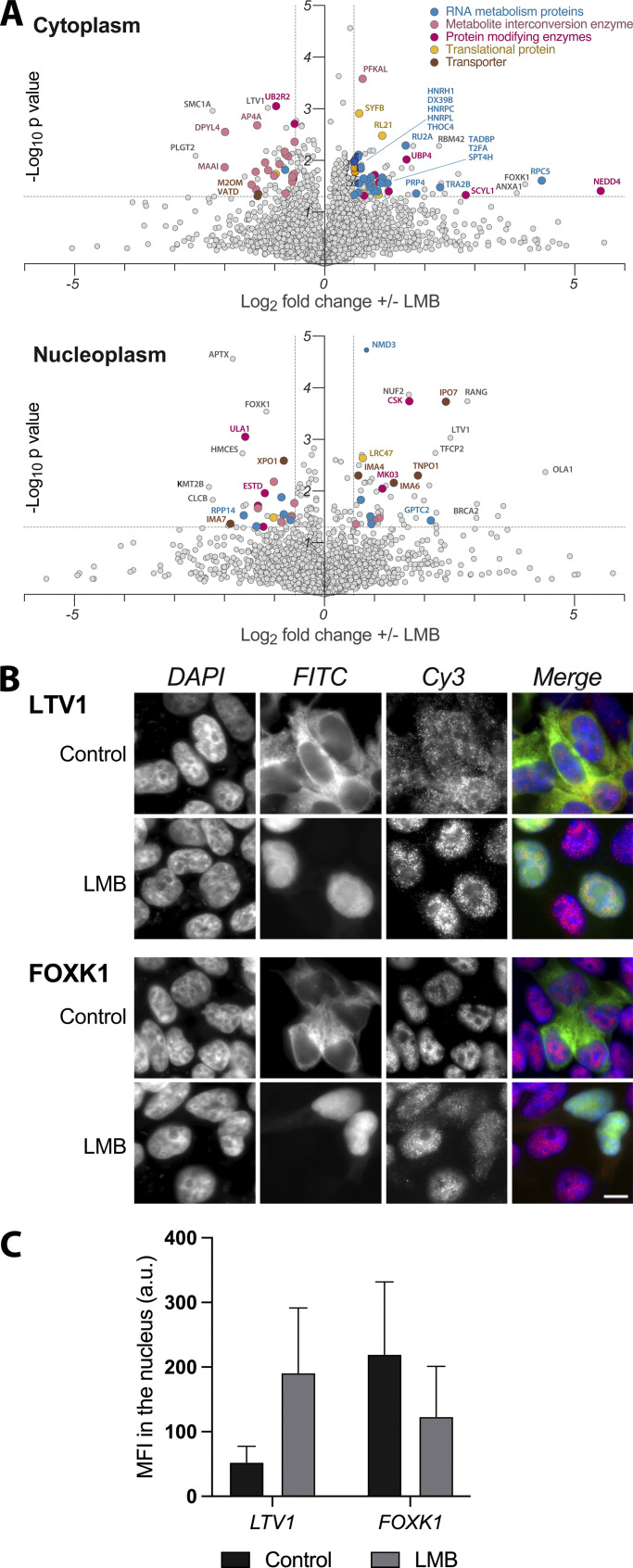
**MS analysis and confirmation of Crm1 inhibition in HEK293T cells. (A)** Volcano plots of the cytoplasm and nucleoplasm fractions representing the redistribution of key proteins as a result of Crm1 inhibition. The proteins with a fold change >1.5 and P value <0.05 are considered significantly changed and are categorized according to protein class. Protein classes are color coded: Cyan—RNA metabolism proteins, pink—metabolite interconversion enzymes, magenta—protein modifying enzymes, yellow—translational proteins, brown—transporters. **(B)** Inhibition of Crm1 in HEK293T cells results in redistribution of key proteins. Immunofluorescence images of HEK293T cells stably expressing GFP_2_-NLS-NES. Top panel: LTV1 (Cy3), bottom panel: FOXK1 (Cy3). Note the change in GFP signal upon LMB treatment. Scale bar, 10 μM. Fluorescent images were obtained with the Revolve R4, Model: RVL2-K2 with Olympus 60× Plan Fluorite Oil IRIS Phase 3 objective with an NA of 1.25 at room temperature at room temperature. Images were acquired with Echo Pro version 6.4.1. and processed with Photoshop. **(C)** Quantification of the nuclear MFI for LTV1 and FOXK1 +/− LMB. Approximately 700 nuclei were quantified for each target and condition and the MFI was averaged for each condition. Error bars represent SD calculated using Microsoft Excel.

**Figure 6.**
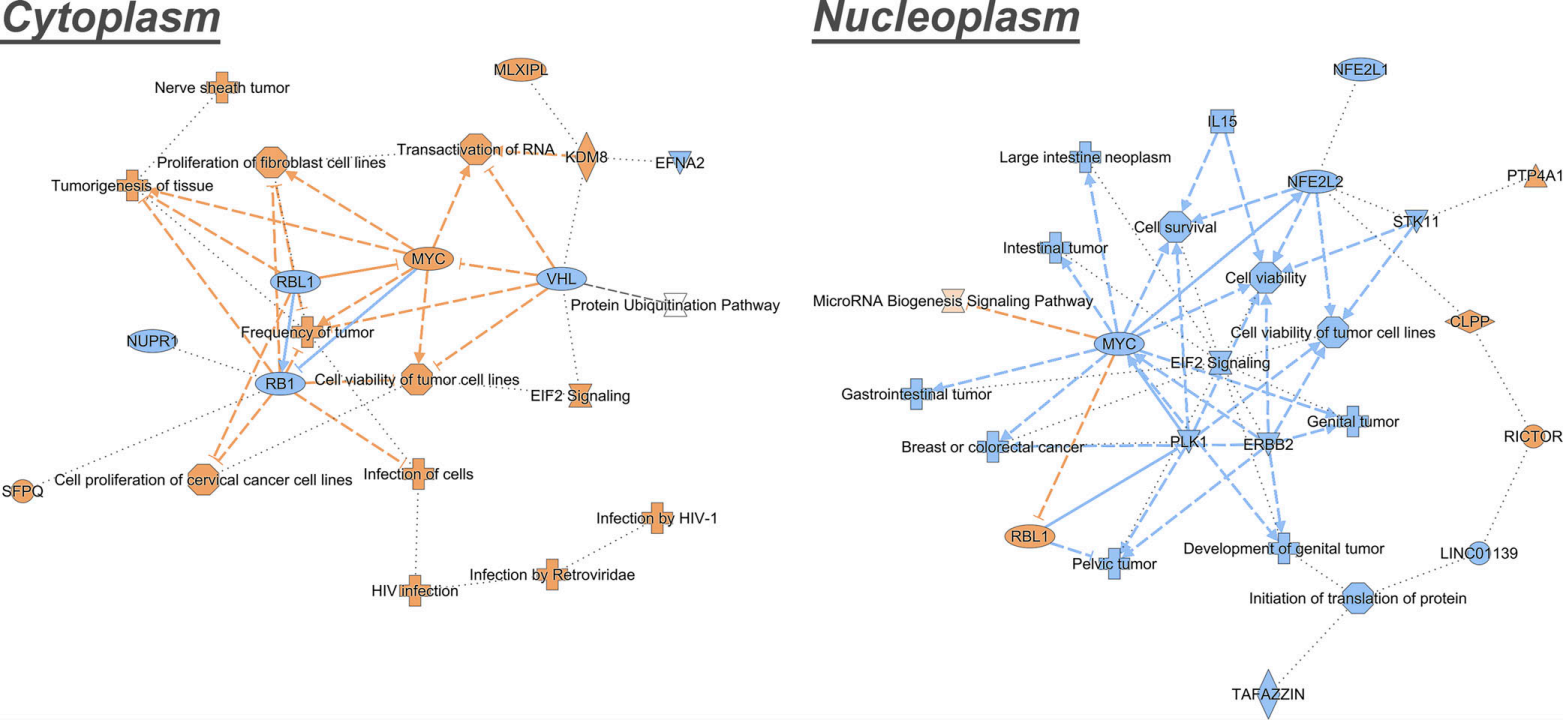
**Canonical pathway analysis reveal correlation between the cytoplasm and the nucleoplasm after Crm1 inhibition.** Orange pathways indicate increase after treatment. Blue pathways indicate decrease after treatment.

We augmented our studies with an assay designed to test the ability of our method to analyze alterations in nucleocytoplasmic proteome distributions. For this purpose, we focused on nuclear export mediated by Crm1 (Xpo1), for three reasons. First, because it mediates the bulk of nuclear protein export (proteins carrying nuclear export sequences [NESs]), we might expect inhibition of Crm1-mediated export to generate a significant nucleocytoplasmic proteome alteration. Second, this alteration—provided that its duration is relatively brief—should be largely due to nucleocytoplasmic redistribution rather than general pleiotropic changes in total protein levels due to loss of cell viability, and so detectable only through a reliable nucleocytoplasmic fractionation protocol (Azizian and Li, 2020; Ferreira et al., 2020; Fukuda et al., 1997; Watanabe et al., 1999). Third, Crm1 alterations are implicated in numerous cancers, as it exports tumor suppressors and oncogenes and many of these proteins were found to be mislocalized in cancer cells (Hill et al., 2014); thus, elevated Crm1 expression levels were found in a wide variety of cancer types (Gao et al., 2015; Inoue et al., 2013; Kojima et al., 2013; Lapalombella et al., 2012; Noske et al., 2008; Schmidt et al., 2013; Shen et al., 2009; Tai et al., 2014; van der Watt et al., 2009; van der Watt et al., 2014; Yao et al., 2009; Yoshimura et al., 2014; Zheng et al., 2014; Zhou et al., 2013). Considering these findings, Crm1 has emerged over the years as a therapeutic target for anticancer therapy, using functional analogs of the Crm1 inhibitor, Leptomycin B (LMB; Green et al., 2015; Parikh et al., 2014; Walker et al., 2013; Zhong et al., 2014); thus, successful characterization of LMB-mediated nucleocytoplasmic redistribution indicates potential utility of the method in a variety of cancer-related studies. In order to find the optimal conditions for Crm1 inhibition, we used HEK293T cells stably transfected with GFP_2_-tagged reporter bearing both an NLS and a Crm1-recognized NES. After even a relatively brief (1 h) incubation with 20 nM LMB, all the GFP signal that was originally in the cytoplasm was localized in the nucleus (Fig. S5 A), and so we fractionated HEK293T cells under these same conditions in order to test the method’s ability to distinguish direct, initial, and perhaps more subtle drug-induced changes, and to avoid toxic and pleiotropic effects on the cells that can be seen with more prolonged LMB exposures (Mutka et al., 2009; Newlands et al., 1996). The resulting cytoplasmic and nucleoplasmic fractions were analyzed by label-free MS, and in order to identify those proteins that were most significantly affected, we chose only proteins with a fold change >1.5 and a P value <0.05. This analysis resulted in a list of ∼100 high likelihood candidate redistributed proteins for each fraction, which we categorized according to protein classes using the PANTHER GeneOntology server (Mi et al., 2013; Mi et al., 2019; Fig. S5 B). Fig. 5 A shows volcano plots of the cytoplasm and the nucleoplasm fractions, with the proteins colored according to protein classes. Notably, Crm1 was redistributed in the cell as a result of LMB treatment as previously reported (Rahmani and Dean, 2017). We also found that RanBP1 was also redistributed in the cell and accumulated in the nucleoplasm under these conditions (Plafker and Macara, 2000). The abundance of metabolite interconversion enzymes is primarily decreased in the cytoplasmic fraction, while increased in the NE (Fig. 5 A and Fig. S5 B). A significantly increased abundance of RNA metabolism proteins was detected in the cytoplasm. This phenomenon is likely due to the changes in mRNA levels, affected by Crm1 inhibition, that exert a significant influence on RNA binding proteins’ localizations (Gilbertson et al., 2018). As for the increased abundance of translational proteins in the cytoplasm, two out of the seven proteins within this category are ribosomal proteins. Ribosomal proteins, like other proteins, are synthesized in the cytoplasm, and then they are actively imported into the nucleus in a karyopherin-mediated process, where they assemble with rRNAs to form the two subunits of the ribosome in the nucleolus (Bassler and Hurt, 2019). In mammalian cells, the import of ribosomal proteins into the nucleus is mediated by RanBP5 and RanBP7 together with RanBP1 (Aitchison and Rout, 2000; Jakel and Gorlich, 1998). Interestingly, when cells were treated with LMB, we detected an increased abundance of RanBP1, RanBP5, and RanBP7 in the nucleoplasm, which may have led to the cytoplasmic accumulation of the ribosomal proteins. Furthermore, the abundance of LTV1, a ribosome biogenesis factor and known export cargo for Crm1 (Seiser et al., 2006; Thakar et al., 2013), was found to increase in the nucleoplasm while decreasing in the cytoplasm (Fig. 5, A, B [upper panel], and C). Another ribosome biogenesis factor with increased abundance in the nucleoplasm is NMD3, a Crm1-interacting pre-ribosomal subunit export adapter (Bai et al., 2013; Thakar et al., 2013). Another noteworthy class of proteins that show a major change in distribution is protein modifying enzymes. Within this category, there are several ubiquitin-related proteins. Protein ubiquitination is implicated in the control of many cellular processes and ubiquitin metabolism enzymes have been identified as either oncogenes or tumor suppressors in various types of cancers (Shi and Grossman, 2010). Other notable proteins with altered nucleocytoplasmic distribution upon LMB treatment include the BRCA2 protein, a known tumor suppressor with key roles in DNA repair (Andreassen et al., 2021), which was found in increased abundance in the nucleoplasm; the ability of Crm1 inhibitors to increase the nucleoplasmic abundance—and so activity—of tumor suppressors such as BRCA2 has been suggested as a major mechanism for their anti-cancer activities (Han et al., 2008; Nakanishi et al., 2007). In the same vein, the cytoplasmic abundance of FOXK1, a transcription factor that was recently reported to be correlated with tumor progression in multiple malignancies (Wencong et al., 2020), is increased while decreased in the nucleoplasm (Fig. 5, A, B [bottom panel], and C)—presumably reducing its tumorigenic activity. Since Crm1-related defects are implicated in various cancers, we sought to supplement this analysis with a canonical pathway analysis using the “Ingenuity Pathway Analysis of complex omics data” (IPA; Ingenuity Systems, Qiagen) in order to have a “bird’s eye” view of the oncogenic events associated with Crm1 inhibition. Notably, analysis of the cytoplasmic and nucleoplasmic fractions shows complementarity; certain pathways represented by components that decrease in the nucleoplasm correspondingly increase in the cytoplasm. For example, components of both the MYC and EIF2 signaling pathways are increased in the cytoplasmic fraction and decreased in the nucleoplasmic fraction (Fig. 6). The MYC oncogene family is causally associated with many types of cancers and its deregulated expression is frequently associated with poor patient prognosis and survival (Chen et al., 2018). MYC proteins are transcriptional modulators involved in many cellular processes including cell growth, cell cycle, apoptosis, and protein translation (Beaulieu et al., 2020; Carroll et al., 2018; Dang, 2016; Duffy et al., 2021; Varmus, 2017). MYC proteins are localized in the cell nucleus and its redistribution to the cytoplasm due to LMB treatment may contribute to their inactivation. Noteworthy, IPO7 and Crm1 genes were previously reported as a positive transcriptional target of c-MYC (Golomb et al., 2012). The redistribution of these proteins as a result of Crm1 inhibition supports the changes we observed for MYC pathway. As for the EIF2 pathway, the nuclear localization of phosphorylated eIF2a has been reported in several metastatic melanoma cell lines (Maida et al., 2019); hence, the redistribution of the EIF2 pathway in the cytoplasm may play an anti-tumorigenic role.

## Discussion

We have developed a rapid, reproducible, and efficient method to fractionate cultured mammalian cells. This protocol does not require any unusual equipment or reagents. In addition, this method is applicable to a range of cell lines. Importantly, the protocol does not require large amount of cells; cells from a confluent 100-mm tissue culture plate are sufficient for a successful fractionation. Finally, this protocol produces only four samples, a sample for each fraction of the cell, and all the cell content is accounted for since there are no wash/discard steps. These two factors allow easy downstream processing for traditional biochemical analyses as well as for in-depth proteomic analyses. Moreover, since this method does not use extreme conditions, the resulting fractions are compatible for further downstream applications where the subcomplex interactions within each compartment are maintained. Since we have demonstrated that the fractions obtained are highly enriched, this provides a reliable means of monitoring proteins of interest in the different cellular compartments.

There are many fractionation methods and commercial kits available; however, they are usually either lengthy, expensive, or require special equipment. The limitations of the commercially available kits have been described above: in particular, limited and incomplete enrichment of the nuclear fraction (Murray et al., 2009), protein leakage from the nuclear fraction (Liu and Fagotto, 2011; Ogawa and Imamoto, 2021), and loss of integrity of the fractionated organelles and component complexes (Huber et al., 2003). Here, we have demonstrated that there is no appreciable leakage from the nuclei and the nuclei and NEs are maintained as morphologically and proteomically intact.

As mentioned above, mislocalization of proteins within the cell contributes to the pathogenesis of many human diseases (Hung and Link, 2011). The availability of reliable and reproducible methods to study these mislocalization events is crucial for a better understanding of these events and may perhaps lead to better therapeutic opportunities in the future. Hence, as a proof of concept, we applied our method to a disease-relevant target, Crm1, to gain a better understanding of the processes and pathways affected by its inhibition. Label-free MS of the fractions allowed us to map the resulting changes demonstrating that the current method can be an important tool for the study of potentially therapeutic nucleocytoplasmic trafficking targets.

Overall, we found a strong interplay between import and export through the NPC and how mislocalization of transport factors plays a significant role in the process. Thus, while one might expect simply to see accumulation of a large set of proteins in the nucleus as a result of Crm1 inhibition, instead we show here that (i) Crm1 export inhibition exerts a subtle yet significant redistribution of a relatively small set of proteins, as was also previously reported in frog oocytes (Wuhr et al., 2015) and (ii) nonintuitively, some of the proteins displayed increased abundance in the cytoplasm, presumably by virtue of perturbation of this import/export interplay.

Finally, although each cellular compartment has its own characteristic set of marker proteins, numerous proteins display a complex distribution throughout the different fractions, likely not just simply due to inefficient fractionation but, based on the faithful fractionation of other such markers, reflective of intracellular communication between the different cellular compartments and distributed functionalities of those proteins in the different compartments.

## Materials and methods

### Tissue culture

Tissue culture cell lines (HEK 293T (CVCL_0063), HeLa (CVCL_0030), HOS (CVCL_0312), and HT1080 (CVCL_0317)) were maintained in growth media, Dulbecco’s modified Eagle’s medium (11965092; Gibco) with penicillin–streptomycin (100 U/ml; Life Technologies), and 10% (v/v) fetal bovine serum (Cat. #F2442; Sigma-Aldrich). N2A cells were maintained in growth media, Dulbecco’s modified Eagle’s medium: Nutrient Mixture F-12 (10565018; Gibco) with penicillin–streptomycin (100 U/ml; Life Technologies), and 10% (v/v) fetal bovine serum (Cat. #F2442; Sigma-Aldrich). Transfections into cells for transient and stable transgene expression were performed with the Lipofectamine LTX with Plus Reagent (15338100; Invitrogen) according to the manufacturer’s protocol. Once transfected, stable cells were selected using Geneticin (10131027; 1 μg/ml; Invitrogen) in growth media.

### Molecular cloning

The pHR39-CMV-GFP-LacZ plasmid backbone with or without the nucleoplasmin NLS sequence was a generous gift from Melissa Kane (University of Pittsburgh). We then replaced the EGFP sequence in these plasmids with an mCherry sequence from the pRS426-GPD-mCherry-4xMS2 plasmid using Gibson cloning procedure, as per manufacturer specifications (Serganov et al., 2022).

The pcDNA3-EGFP plasmid was a kind gift from Sigi Benjamin-Hong (Rockefeller University, New York, NY). This plasmid was used to prepare new plasmid with NES/NLS/NLS-NES sequence according to Abkallo et al. (2011); Saito et al. (2004).

To generate pcDNA3-EGFP-NES, an insert DNA fragment was prepared using oligonucleotides encoding LALKLAGLDI from human PKIα NES. Two oligonucleotides, 5′Phos-AATTTAGCCTTGAAATTAGCAGGTCTTGATATCG-3′ and 5′Phos-GATCCGATATCAAGACCTGCTAATTTCAAGGCTA-3′, were annealed and ligated into EcoRI- and BamHI-digested pcDNA3-EGFP.

To generate pcDNA3-EGFP-NLS, an insert DNA fragment was prepared using oligonucleotides encoding PKKKRKV from SV40 large T antigen. Two oligonucleotides, 5′Phos- GAT​CTC​CAA​AAA​AGA​AGA​GAA​AGG​TAC​A-3′ and 5′Phos- AGC​TTG​TAC​CTT​TCT​CTT​CTT​TTT​TGG-3′, were annealed and ligated into HindIII and BglII digested pcDNA3-EGFP.

To generate pcDNA3-EGFP-NLS-NES, an insert DNA fragment was prepared using oligonucleotides encoding PKKKRKV from SV40 large T antigen. Two oligonucleotides, 5′Phos- GAT​CTC​CAA​AAA​AGA​AGA​GAA​AGG​TAC​A-3′ and 5′Phos- AGC​TTG​TAC​CTT​TCT​CTT​CTT​TTT​TGG-3′, were annealed and ligated into HindIII and BglII digested pcDNA3-EGFP-NES.

### Fractionation of mammalian cell lines

Each fractionation procedure was carried out using cells from a 90 to 95% confluent 10 cm plate. Cells were washed with PBS and harvested with 1 ml trypsin (Trypsin-EDTA (0.25%) at 37°C for 1 min. Cells were pelleted at 10 g for 10 min at 4°C and resuspended in 1-ml pre-chilled PBS and pelleted again at 50 g for 2 min at 4°C. The supernatant was carefully removed, and cells were gently resuspended in lysis buffer (see below; 1:5-1:8 v/v depending on the cell line), vortexed for 5 s, and left on ice for 5 min to swell. Cells were next lysed with needle strokes using a 1-ml insulin syringe with a 28 gauge needle. Lysis was monitored by phase contrast microscopy for completion and dispersal of cytoplasmic material away from nuclei. Lysed cells were underlaid with 200 μl 20% sucrose in 8% PVP buffer and pelleted at 3,500 g for 10 min 4°C. The supernatant, including the sucrose phase, was collected (cytoplasmic fraction). The pellet was resuspended with 1 ml 6% PVP supplemented with 1:100 solution P and 1 mM DTT and processed with a handheld polytron (PT 1200 E) twice for 10 s at mid power setting. In the absence of a polytron, it is possible to resuspend the nuclei pellet with repeated pipetting up and down of a 1.0 ml Pipetteman tip. While not as efficient as the Polytron, this method is suitable for many purposes. The dispersed nuclei was underlaid with 1 ml of 2.01 M Sucrose in 8% PVP and 1 mM DTT and centrifuged at 50,000 *g* for 40 min at 4°C in a SW55 rotor (Beckman ultracentrifuge). The interface and the sucrose underlay were collected as the membrane fraction and the pellet constituted the nuclear fraction. To further extract the NE fraction, the pelleted nuclei were resuspended in DNase buffer (see below, 10 × 10^7^ cells resuspended in 1 ml DNase buffer). Nuclei were vortexed briefly and incubated at room temperature for 15–30 min. The digestion progress was monitored by phase contrast microscopy, where the NE was visualized as “C”-shaped structures. The suspension was underlaid with 100 µl of 30% sucrose in 20 mM HEPES, pH 8, 0.1 mM MgCl_2_ and 1:100 Solution P, and the NE was pelleted at 18,000 *g* for 10 min at 4°C in Optima Ultracentrifuge. The supernatant was then collected and considered to be the nucleoplasm fraction, while the pellet contained the NE fraction. For downstream SDS-Page analyses and Western blot samples were methanol precipitated: 900 μl methanol was added to 100 μl of the sample. Samples were vortexed and incubated in −20°C overnight. Next, samples were pelleted at 20,000* g* for 20 min at 4°C, resuspended by 2 s sonication in 500 μl methanol, incubated at −20°C for 20 min, and pelleted again. The resulting pellet was resuspended in 50 μl buffer A and sonicated for 5 s.

### Buffers

#### Phosphate-buffered saline (PBS): 137 mM NaCl, 2.7 mM KCl, 10 mM Na_2_HPO_4_, 1.5 mM KH_2_PO_4_

Solution P: 20 mg/ml phenylmethylsulfonyl fluoride, 0.4 mg/ml Pepstatin A in ethanol.

8% PVP buffer: 8% PVP-40, 20 mM K-Phosphate, 7.5 μM MgCl_2_. pH adjusted to 6.53. with concentrated H_3_PO_4_.

Lysis buffer: 6% PVP, 0.015% Digitonin, 0.015% Triton X-100, 4 μM Cytochalasin B, 1:100 Solution P (100 mg of phenylmethylsulfonyl fluoride, 2 mg of pepstatin A in 5 ml of ethanol), 1 mM DTT (0.045% detergent is required to lyse HeLa and HT1080 cells).

DNase buffer: 10% sucrose in 20 mM HEPES, pH 8 and 0.1 mM MgCl_2_, 10 μM CaCl_2_, 1 mM DTT, 100 μg/ml Heparin, 0.1 μg/ml RNase, 1:1,000 DNase (DNase I (Sigma-Aldrich, DN-EP): Resuspended at 5 mg/ml in buffer containing 50% glycerol, 10 mM Tris–HCl (pH 7.4), 50 mM NaCl, 1 mM DTT, and 2 mM MgCl_2_), 1:100 Solution P.

Buffer A: 0.5 M TRIS base, 5% SDS.

### Protocol notes

A handheld Polytron is highly recommended for this protocol as it is a low-cost laboratory instrument; however, in the absence of a polytron nuclei, can be resuspended with repeated pipetting.

### HeLa cells fractionation with commercial kits

Hela cells were fractionated using #78833, Thermo Fisher Scientific; #AR0106, Boster; and #ab109719, Abcam as per manufacturer’s instructions.

### Quantitative immunoblotting

Proteins were measured using a capillary-based electrophoresis instrument (Wes, ProteinSimple). Protein amounts (0.5 mg/ml) were preoptimized for and were denatured using manufacturer-supplied reagents and loaded into multi-well plates. Protein separation and detection were performed via capillary electrophoresis, antibody binding, and HRP-conjugated visualization following the manufacturer’s instructions. Antibody optimization was completed for all proteins for which Wes analysis was performed to determine ideal dilution conditions. Antibodies used are listed in Table 3. Analysis was performed using the Compass software for Simple Western (ProteinSimple). Chemiluminescence signals were normalized as material derived from the same total number of cells in each fraction, and enrichment percentage were calculated accordingly.

**Table 3.** List of antibody dilutions used for Wes immunoblotting

Antibody	Supplier	Catalog #	dilution
LAMIN A/C	Santa cruz	sc-20681	1:500
GAPDH	CST	2118S	1:500
COX IV	CST	11967S	1:100
b-actin	CST	4970S	1:200
mCherry	CST	43590S	1:500
Histone H2.A	CST	2718S	1:100
Nup62	BD bioscience	610497	1:2,000
Nup214	Abcam	ab70497	1:100
Nup153	CST	98559S	1:100
Nup133	Santa cruz	sc-376763	1:100
Nup88	BD bioscience	611896	1:100
Nup85	Santa cruz	sc-376111	1:200

### Immunofluorescence of mammalian cell lines

HEK293T cells were transiently transfected in 24-well plate on poly-L-lysine-coated cover slips to express mCherry-LacZ fusion proteins with or without NLS (Serganov et al., 2022). Cells were washed with PBS, fixed in 4% paraformaldehyde (PFA) at room temperature for 15 min, washed with PBS, permeabilized using 0.1% Triton X-100 in PBS for 15 min, followed by three PBS washes. Next, cells were blocked with 5% goat serum, 1% BSA, in PBS at room temperature for 1 h and subsequently nuclei were stained with 300 nM 4′,6-diamidino-2-phenylindole (DAPI) in PBS at room temperature for 10 min. Coverslips were mounted with ProLong Gold Antifade. Fluorescent images were obtained with the Revolve R4, Model: RVL2-K2 with Olympus 60× Plan Fluorite Oil IRIS Phase 3 objective with an NA of 1.25 at room temperature. Images were acquired with Echo Pro version 6.4.1. and processed with Photoshop.

### Nuclear permeability assay

HEK293T cells were fractionated according to protocol above. Nuclear permeability was measured using 1 μM Tetramethylrhodamine Dextran (#D1816, #D1842, and #D1819, Thermo Fisher scientific). Fluorescent images were obtained with the Revolve R4, Model: RVL2-K2 with Olympus 60× Plan Fluorite Oil IRIS Phase 3 objective with an NA of 1.25 at room temperature. Images were acquired with Echo Pro version 6.4.1. and processed with Photoshop.

### Sample preparation for mass spectrometry

Methanol precipitated fractions were reduced and alkylated (25 mM iodoacetamide in the dark) protein samples were run ∼5 mm into a 10% bis-Tris SDS-polyacrylamide gel, and gels were Coomassie-blue stained.

### Mass spectrometric label-free quantification

Proteins in gel plugs were digested and peptides were extracted as described in Bosch et al. (2021). Peptide solution from each biological replicate was divided into two parts and the peptides were bound to C18 StageTips. Peptides eluted from the StageTip were analyzed by LCMS using a Thermo Q Exactive Plus or a Orbitrap Exploris mass spectrometer coupled with an Easy-nLC system (Thermo Fisher Scientific).

SpectroMine (Biognosys AG) software was used for label-free quantitation (LFQ). The protein LFQ outputs from SpectroMine were further analyzed using Microsoft Excel. To compare LFQs across samples from a cell fractionation, including biological replicates and technical replicates, normalization was applied so that the sum of the normalized LFQs for 6–8 abundant proteins from each LCMS run was the same. After normalization, the relative standard deviations of LFQs across samples for these 6–8 proteins were within 20%. Data distribution was assumed to be normal but this was not formally tested. Student *t* test function in Microsoft Excel was used to calculate the P values, with parameters for a two-tailed distribution and two-sample unequal variance (Bosch et al., 2021). The heat maps were derived from percentages of each protein detected by mass spectrometry (Bosch et al., 2021), as distributed between equal total protein loadings from samples of each fraction. Mass spectrometry data are deposited in Zenodo at https://doi.org/10.5281/zenodo.7630027.

### Sample preparation for electron microscopy

Nuclei and NE fractions were fixed in 2% glutaraldehyde (VWR 100503-966) in 0.1 M sodium cacodylate buffer (pH 7.2) for >1 h at room temperature and then overnight at 4ºC, postfixed in 1% osmium tetroxide 1%, en bloc stained in 1% uranyl acetate in 0.05 M sodium maleate buffer (pH 5.2), and processed for Epon embedding.

Ultrathin sections (60–65 nm) were counterstained with uranyl acetate and lead citrate and imaged on a Tecnai 12 electron microscope (FEI, Hillsboro, Oregon), equipped with an AMT BioSprint29 digital camera.

### Crm1 inhibition assay

Cellular fractionation: HEK293T cells stably expressing GFP_2_-NLS-NES were treated with 20 nM Leptomycin B (#L2913; Sigma-Aldrich) or vehicle (70/30 methanol/water) for 1 h at 37°C and the reporter fluorescence was examined with the Revolve R4, Model: RVL2-K2 with Olympus 40× Plan Fluorite Phase Ph2 NA: 0.75 at room temperature.

Immunofluorescence: HEK293T cells stably expressing GFP_2_-NLS-NES were seeded at low density on poly-L-lysine-coated cover slips in 24-well plates. At the desired confluence, the cells were treated with 20 nM LMB or vehicle for 1 h at 37°C. Cells were washed with PBS, fixed in 4% PFA at room temperature for 15 min, washed with PBS, and permeabilized using 0.1% Triton X-100 in PBS for 15 min followed by three PBS washes. Next, cells were blocked with blocking buffer (5% goat serum, 1% BSA in PBS) at room temperature for 1 h and subsequently incubated with primary antibodies LTV1 (1:500, #NBP1-86735; Novus Biologicals) and FOXK1 (1:1,000, #PA5-81177; Thermo Fisher Scientific) diluted in blocking buffer at room temperature for 1 h. After PBS wash, cells were incubated with secondary antibody conjugated to Cy3 (#111-165-144; Jackson ImmunoResearch diluted in blocking buffer 1:3,000) for 1 h at room temperature. After incubation, cells were washed with PBS and nuclei were stained with (DAPI, 300 nM) in PBS at room temperature for 10 min. Coverslips were mounted with ProLong Gold Antifade. Fluorescent images were obtained with the Revolve R4, Model: RVL2-K2 with Olympus 60× Plan Fluorite Oil IRIS Phase 3 objective with an NA of 1.25 at room temperature. Images were acquired with Echo Pro version 6.4.1. and processed with Photoshop.

### Mean fluorescence intensities (MFIs) quantification

Cell nuclei (stained with DAPI) were segmented using the Cellpose software (Stringer et al., 2021), model: cyto2, diameter: 300 pixels; and the resulting label image was saved as a PNG file. A custom script in Fiji was used to convert the Cellpose-generated label image into individual nuclei regions-of-interest (ROIs). The corresponding Cy3 channel of the image, showing LTV1 or FOXK1 distribution, was opened in Fiji and background fluorescence signal was subtracted with a rolling ball radius of 50 pixels. Nuclei ROIs from the DAPI channel were then transferred to the background subtracted Cy3 channel image and MFI was measured for each nucleus.

### Online supplemental material

Fig. S1 shows Wes protein analysis of HeLa cells fractionated using commercial kits. Fig. S2 shows nuclear permeability assay. Fig. S3 shows EM images nuclei and NE from HeLa cells. Fig. S4 shows heatmap of transport factors and membrane markers. Fig. S5 Crm1 inhibition in HEK293T cells. Table S1 list of the proteins and their abundance in Fig. 4. Table S2 list of the proteins and their abundance in Fig. S4 A. Table S3 list of the proteins and their abundance in Fig. S4 B.

## Supplementary Material

Table S1 lists the proteins and their abundance in Fig. 4.

Table S2 lists the proteins and their abundance in Fig. S4 A.

Table S3 lists the proteins and their abundance in Fig. S4 B.

SourceData F2 is the source file for Fig. 2.

SourceData F3 is the source file for Fig. 3.

## Data Availability

The data underlying Figs. 2 and 3 are available in the published article, its online supplemental material, and from the authors upon reasonable request. The data underlying Figs. 4 and 5 and Fig. S5 are openly available in Zenodo at https://doi.org/10.5281/zenodo.7630027.
