# Supplementary material for: A general method for quantitative fractionation of mammalian cells
Source: J Cell Biol. 2023 Mar 15;222(6):e202209062. doi: 10.1083/jcb.202209062 (PMC10040634; doi:10.1083/jcb.202209062)
Supplement: SourceData F2 — is the source file for Fig. 2. [file JCB_202209062_SourceDataF2.pdf]

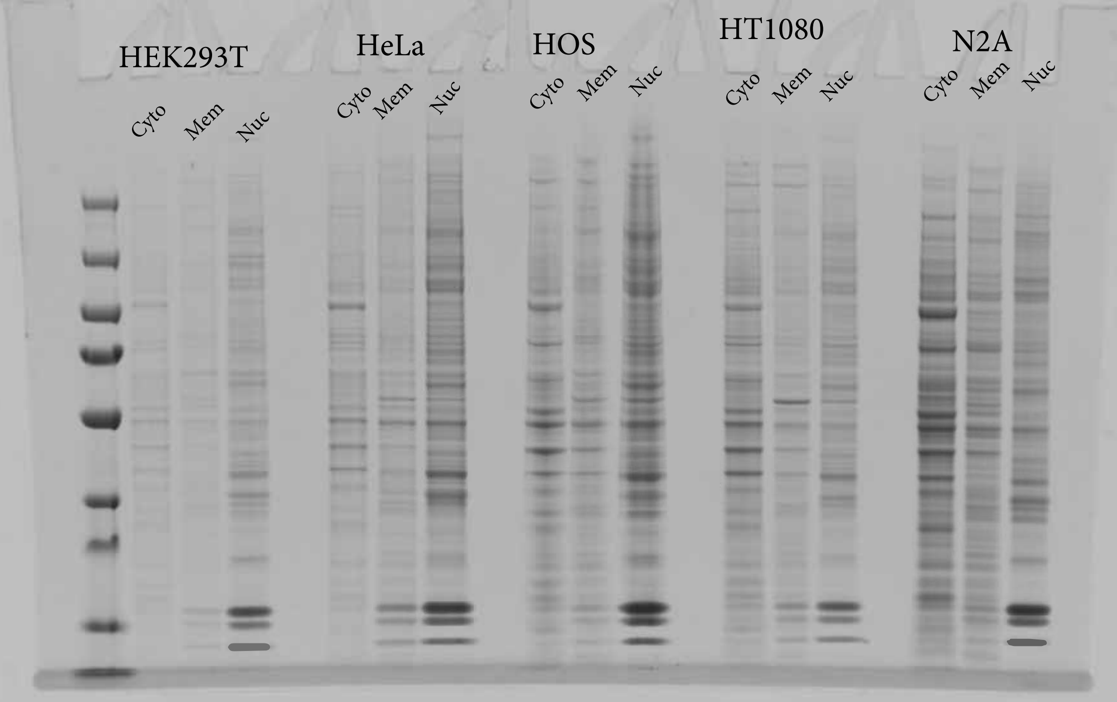

HEK293T
HeLa
HOS
HT1080
N2A
Cyto
Mem
Nuc
Cyto
Mem
Nuc
Cyto
Mem
Nuc
Cyto
Mem
Nuc
Cyto
Mem
Nuc

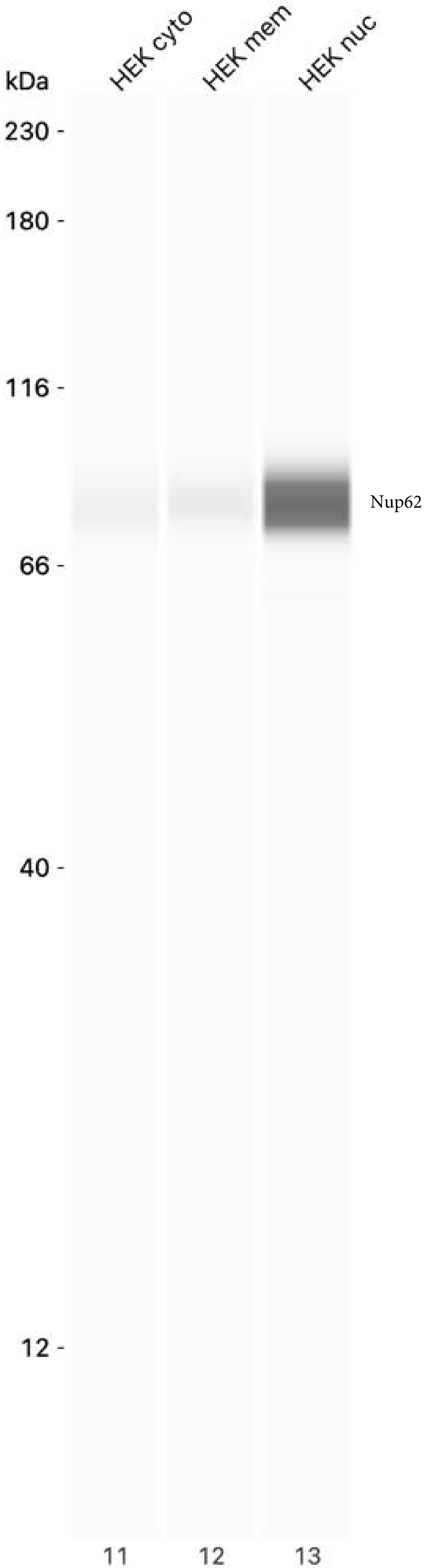
kDa
HEK cyto
HEK mem
HEK nuc
230
180
116
66
40
12
Nup62
11
12
13

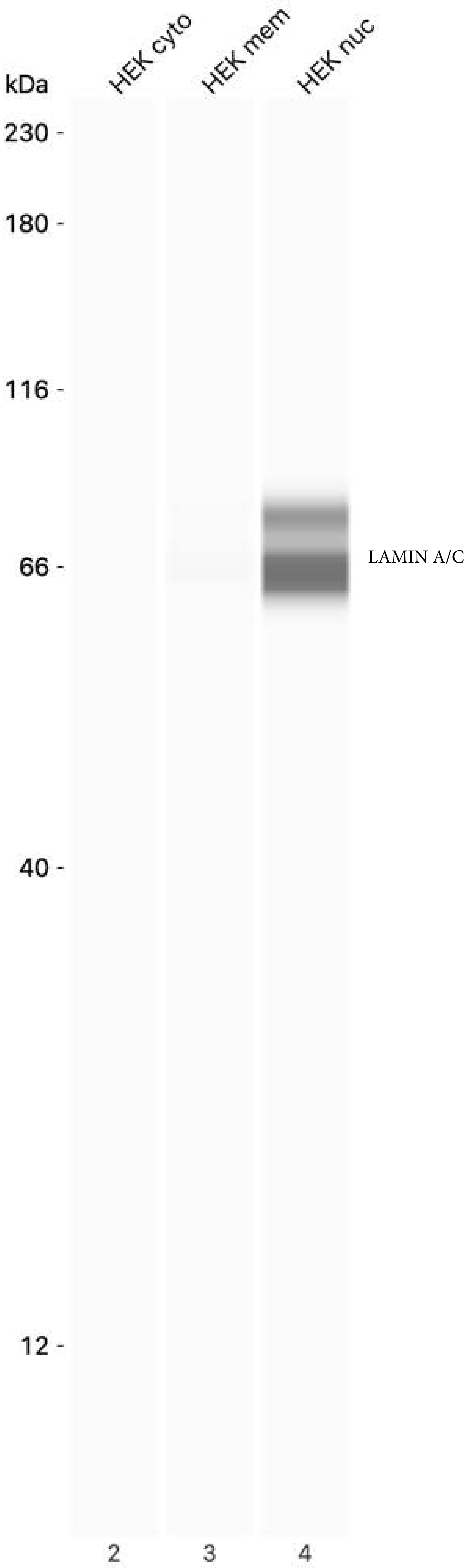
kDa
HEK cyto
HEK mem
HEK nuc
230 -
180 -
116 -
66 -
40 -
12 -
LAMIN A/C
2
3
4

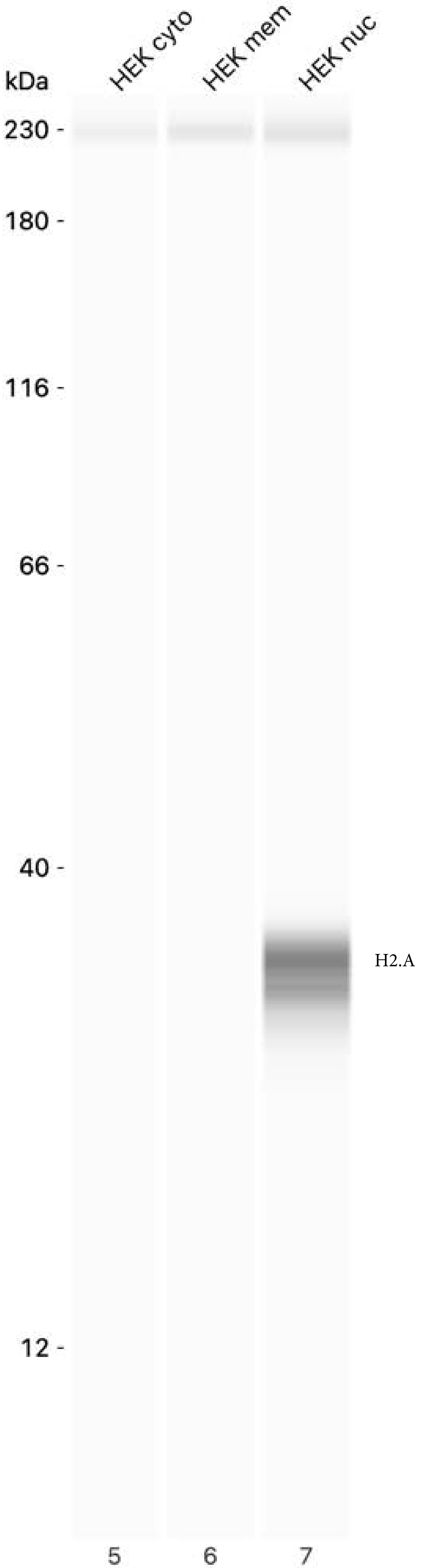
kDa
HEK cyto
HEK mem
HEK nuc
230 -
180 -
116 -
66 -
40 -
12 -
H2.A
5
6
7

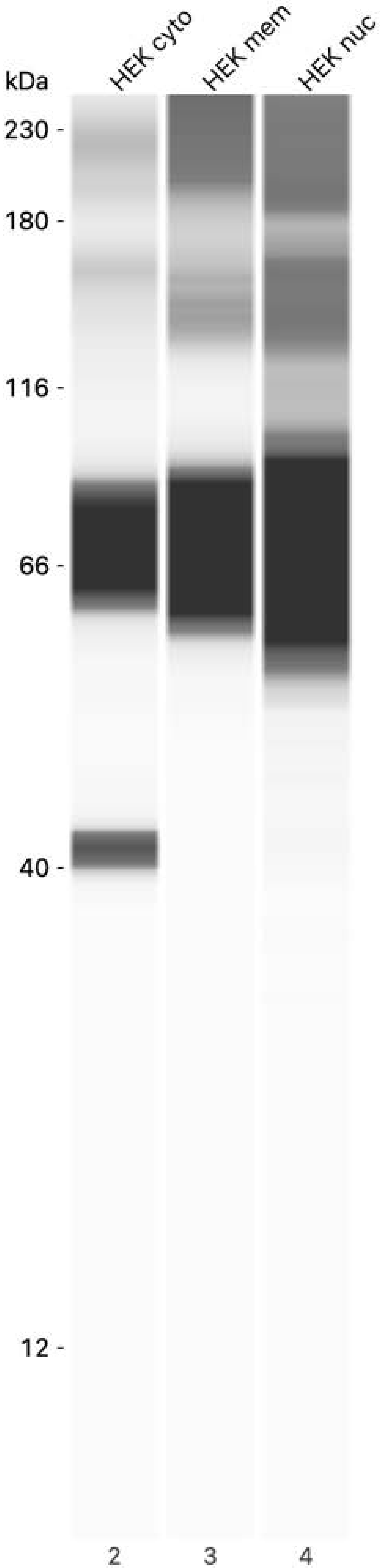

GAPDH

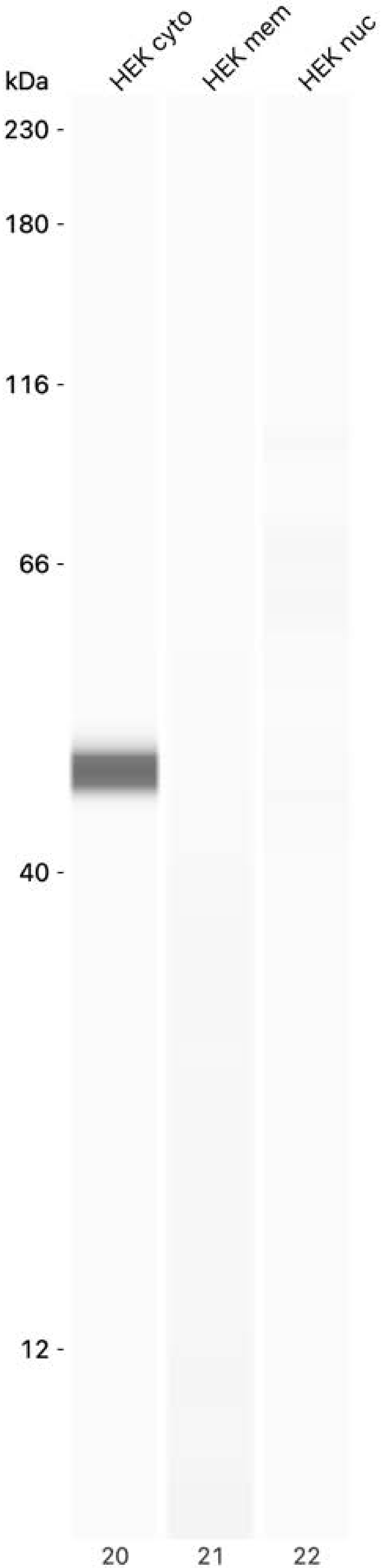

Actin

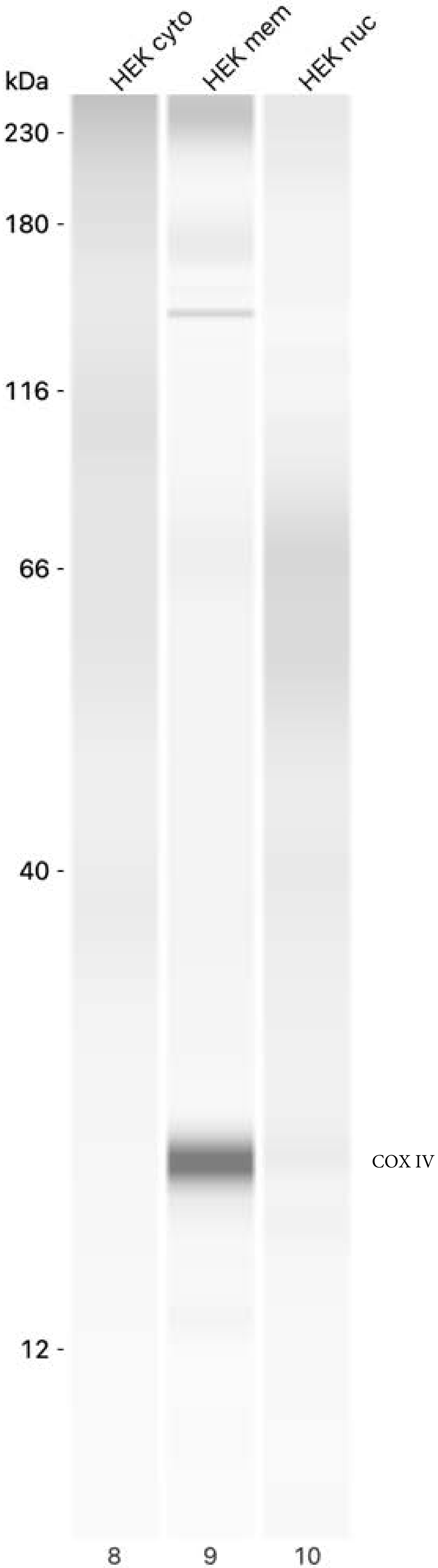

kDa
HEK cyto
HEK mem
HEK nuc
230
180
116
66
40
12
COX IV
8
9
10

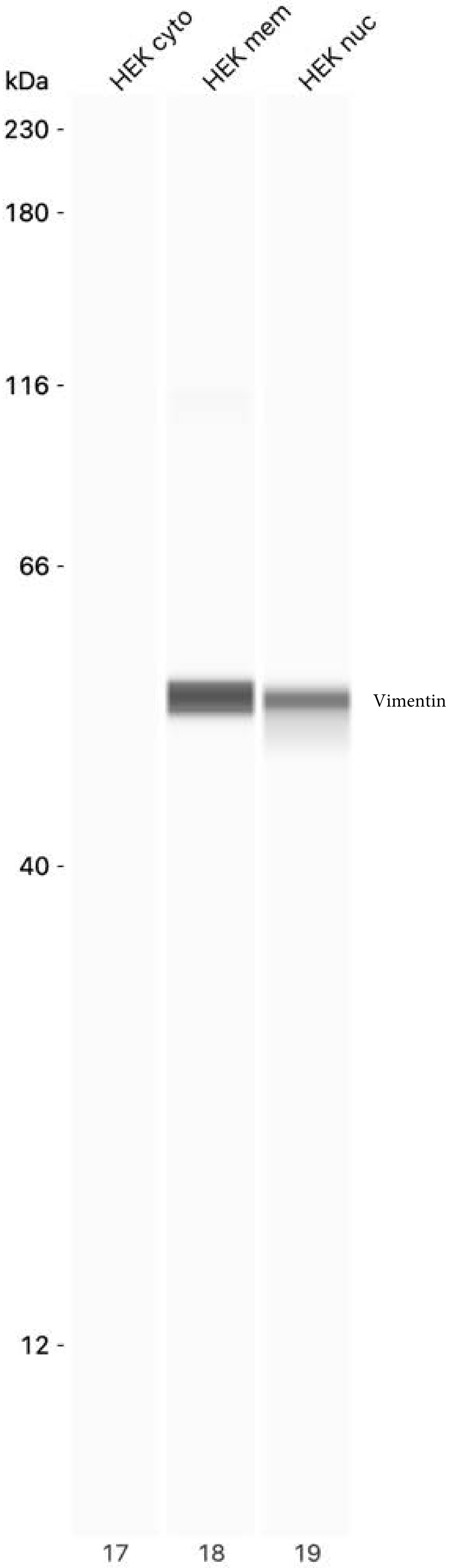
kDa
HEK cyto
HEK mem
HEK nuc
230 -
180 -
116 -
66 -
40 -
12 -
Vimentin
17
18
19

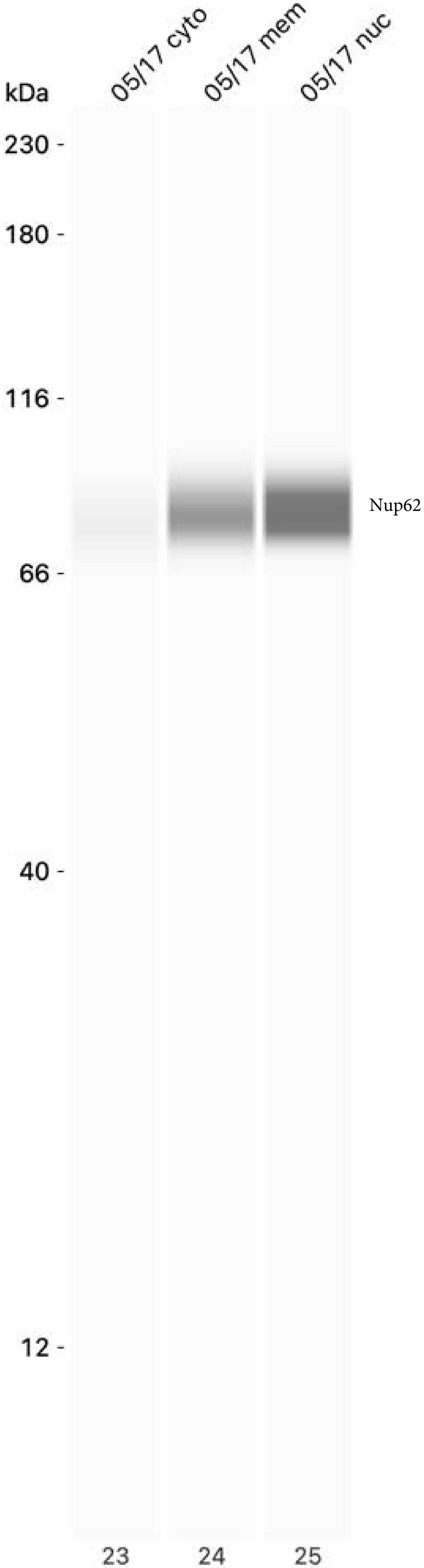
kDa
05/17 cyto
05/17 mem
05/17 nuc
230
180
116
66
40
12
Nup62
23
24
25

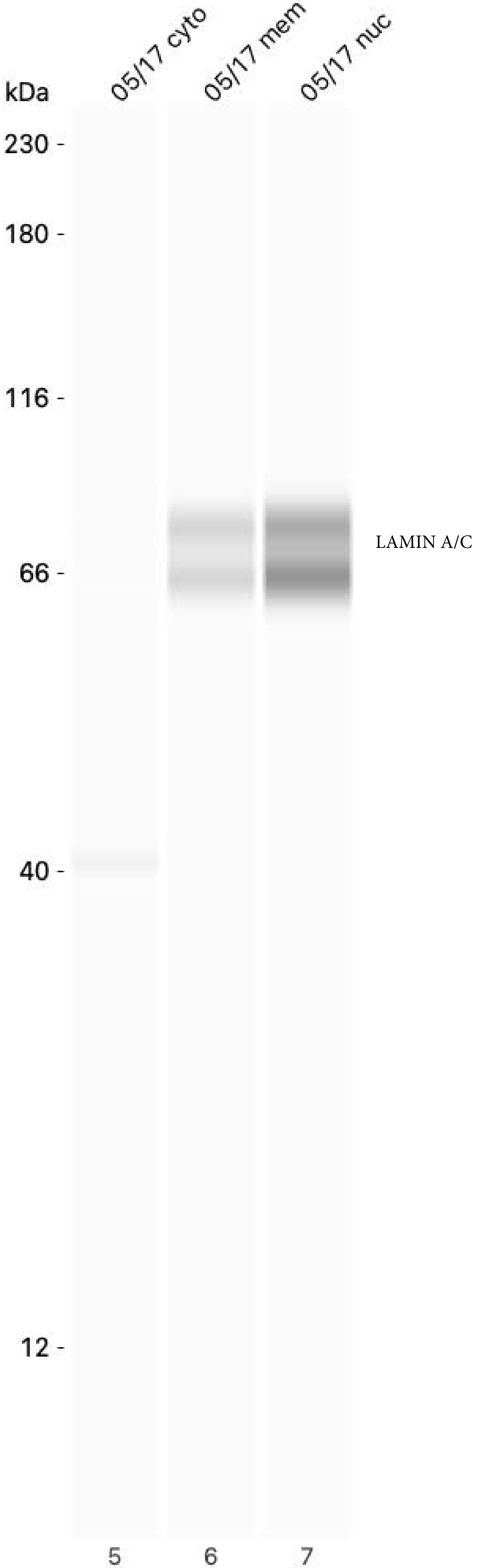
kDa
05/17 cyto
05/17 mem
05/17 nuc
230
180
116
66
40
12
LAMIN A/C
5
6
7

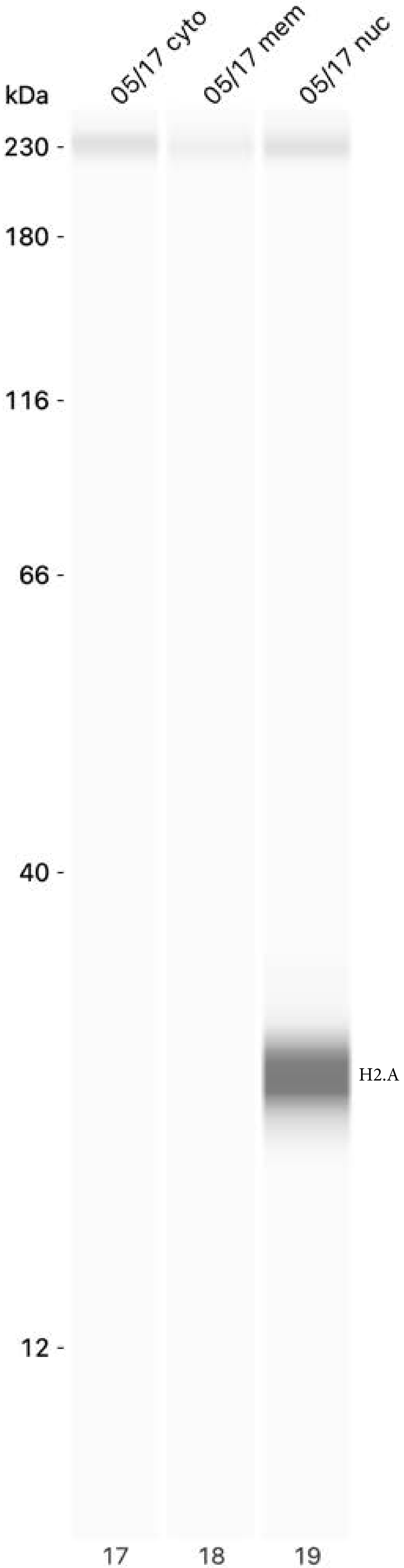
05/17 cyto
05/17 mem
05/17 nuc
kDa
230
180
116
66
40
12
H2.A
17
18
19

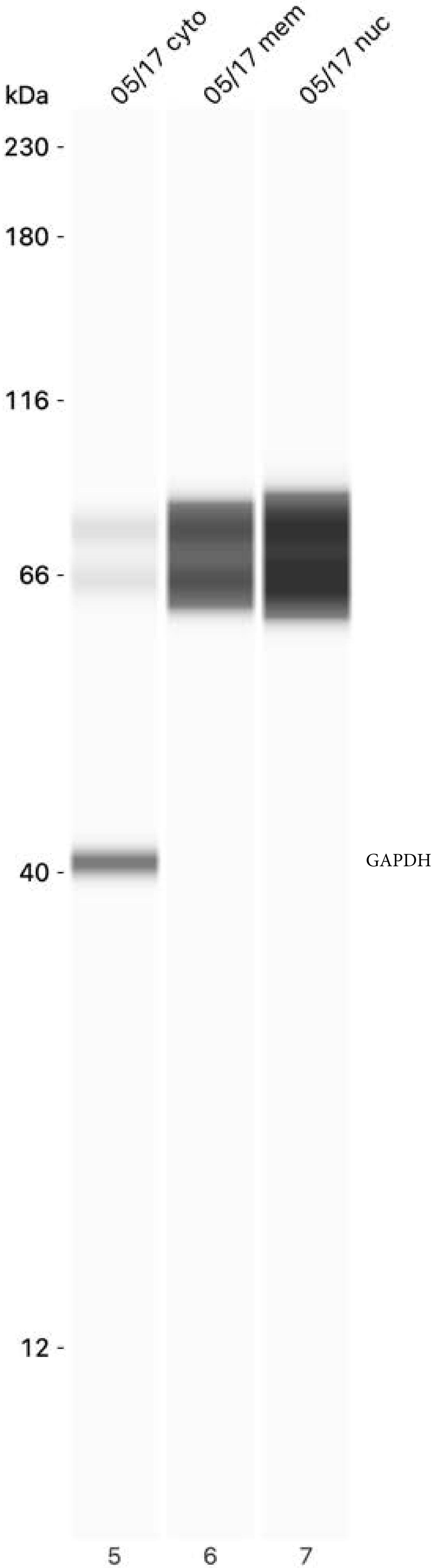

kDa
05/17 cyto
05/17 mem
05/17 nuc
230
180
116
66
40
12
GAPDH
5
6
7

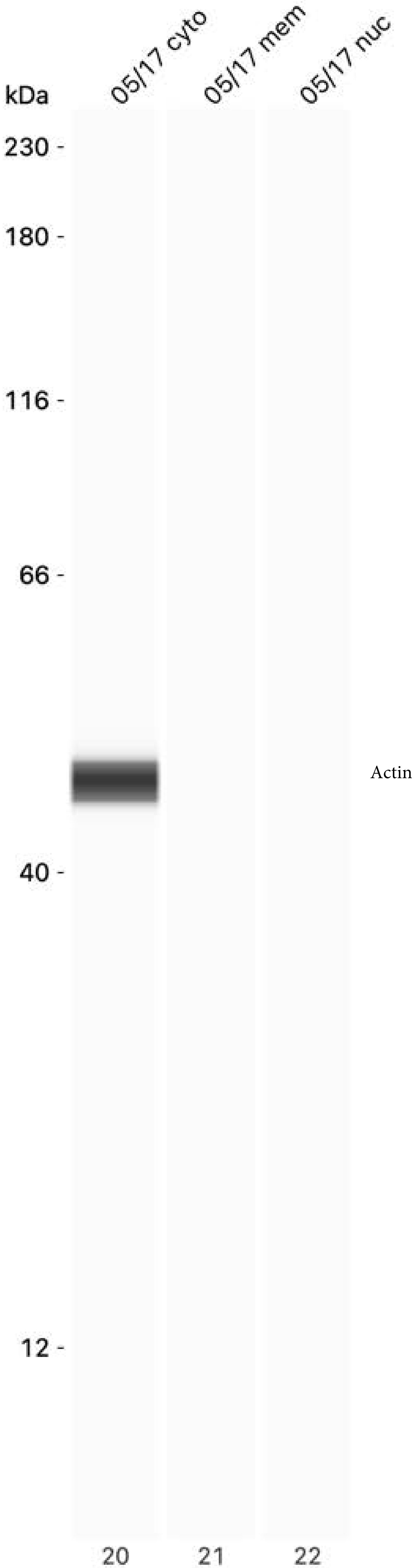
05/17 cyto
05/17 mem
05/17 nuc
kDa
230
180
116
66
40
12
20
21
22
Actin

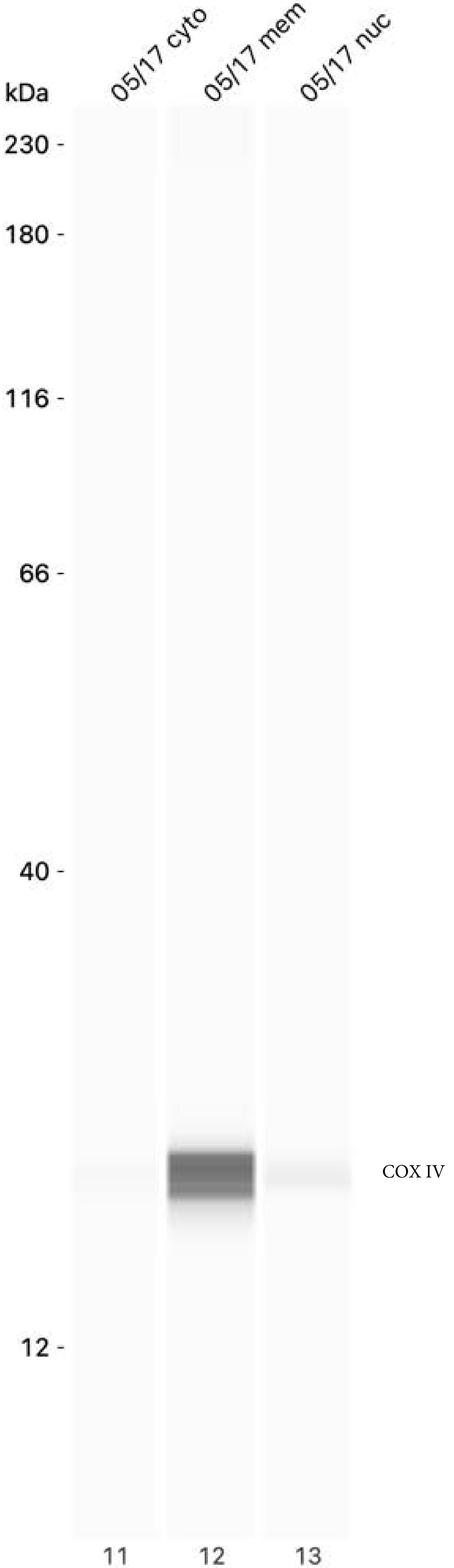
kDa
05/17 cyto
05/17 mem
05/17 nuc
230
180
116
66
40
12
COX IV
11
12
13

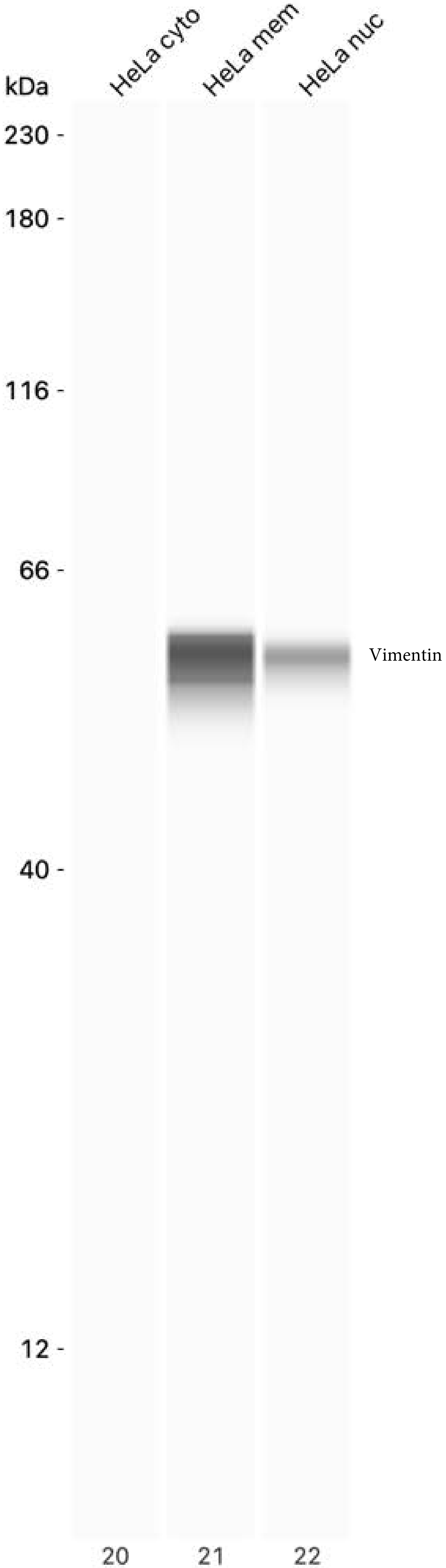
kDa
HeLa cyto
HeLa mem
HeLa nuc
230
180
116
66
40
12
Vimentin
20
21
22

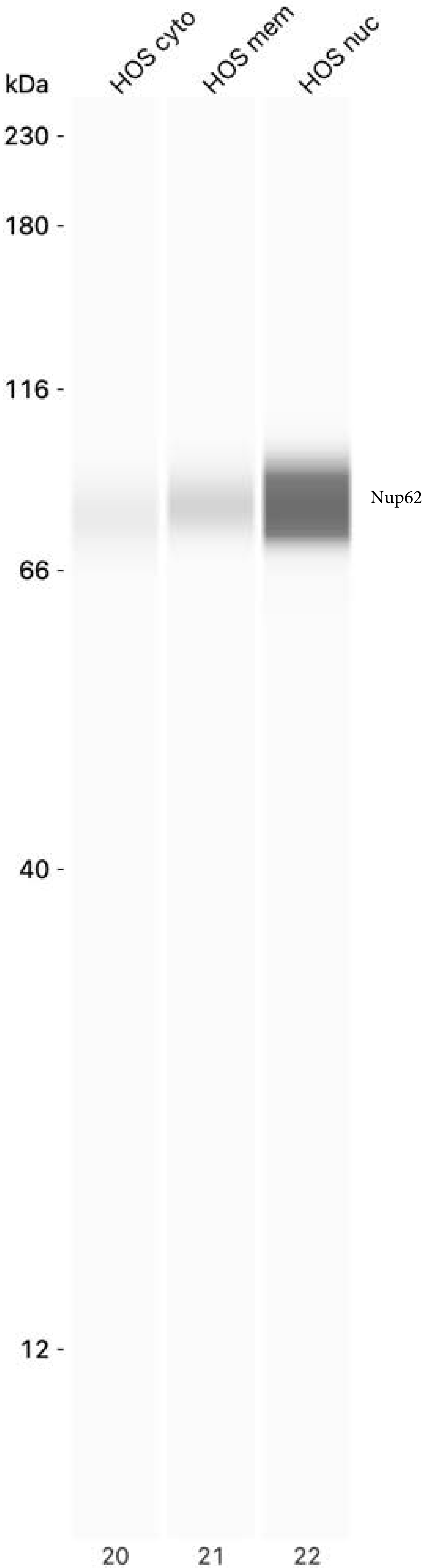
kDa
HOS cyto
HOS mem
HOS nuc
230 -
180 -
116 -
66 -
40 -
12 -
Nup62
20
21
22

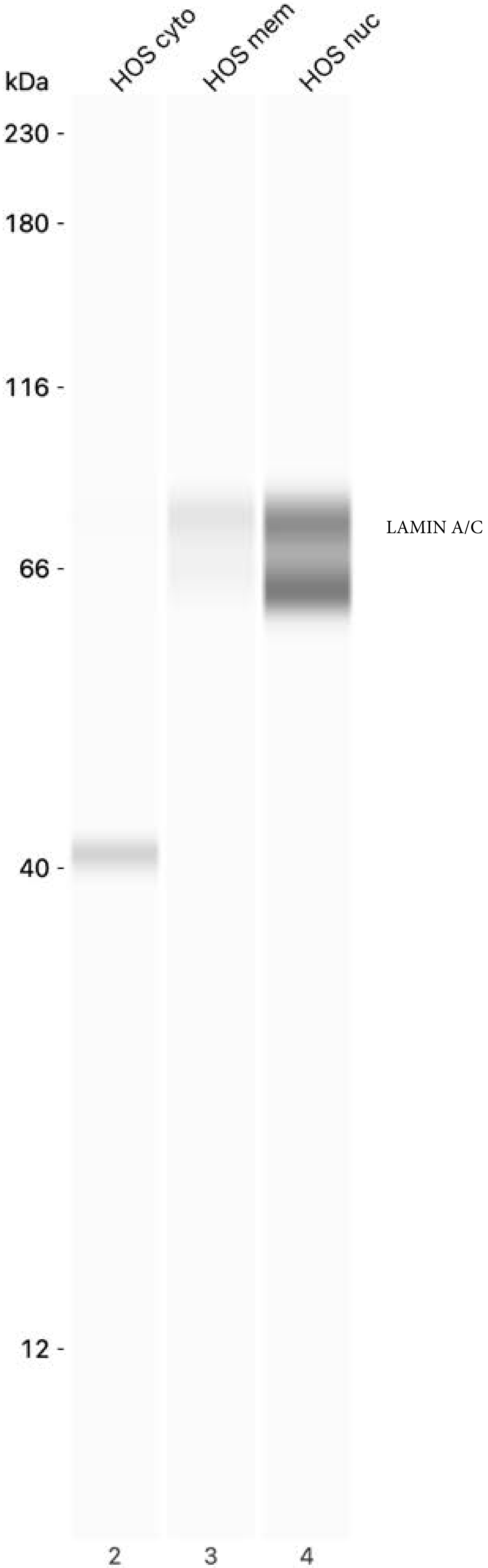
kDa
HOS cyto
HOS mem
HOS nuc
230
180
116
66
40
12
2
3
4
LAMIN A/C

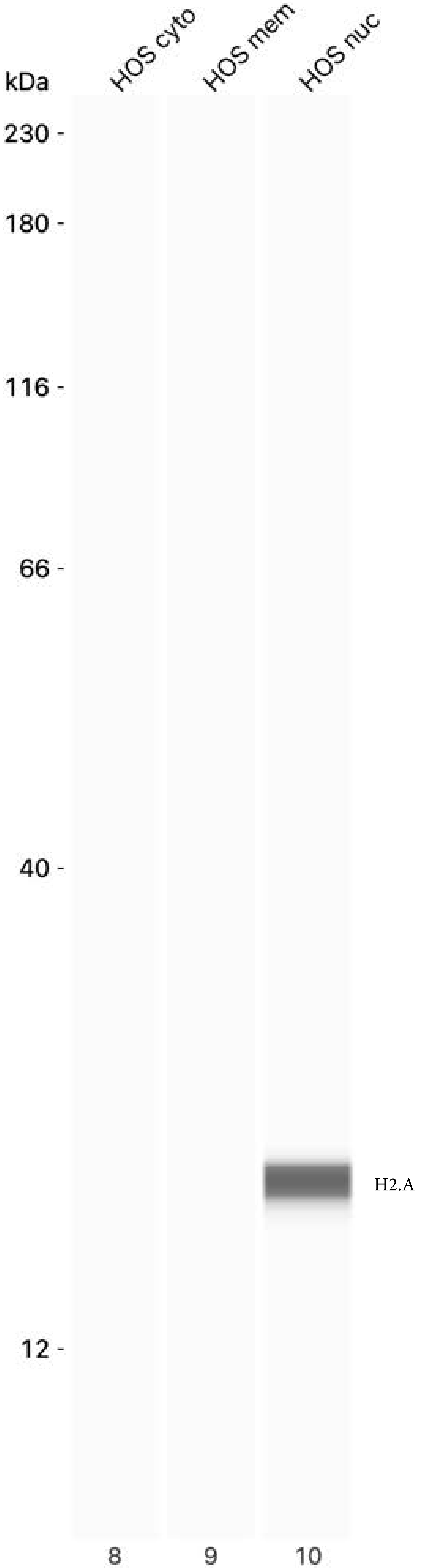
HOS cyto
HOS mem
HOS nuc
kDa
230
180
116
66
40
12
H2.A
8
9
10

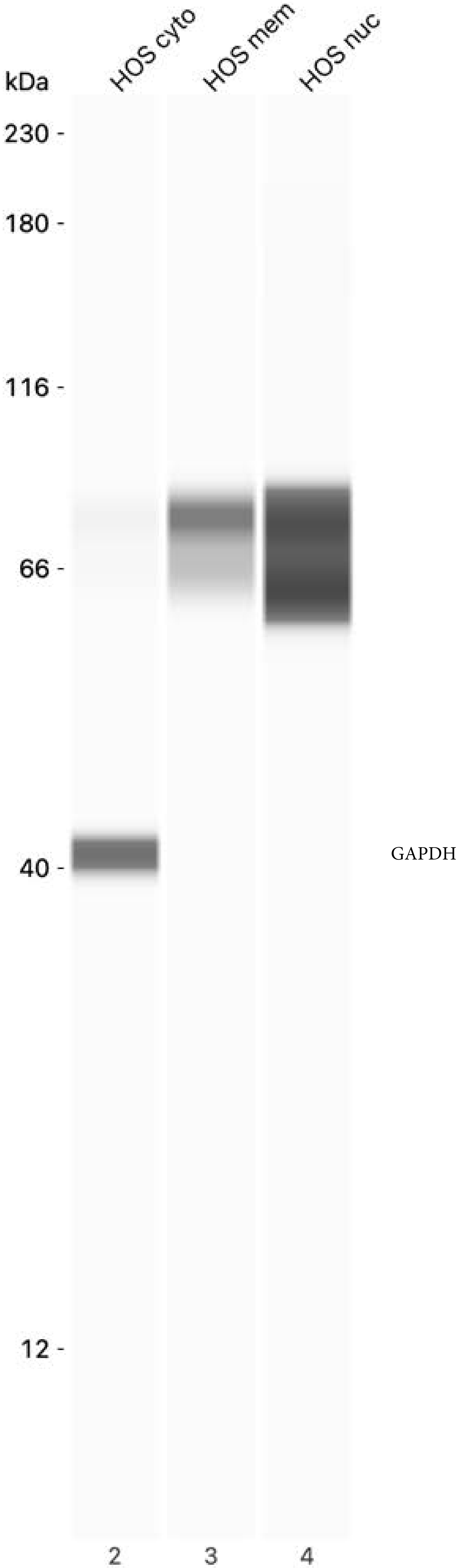
kDa
HOS cyto
HOS mem
HOS nuc
230 -
180 -
116 -
66 -
40 -
12 -
2
3
4
GAPDH

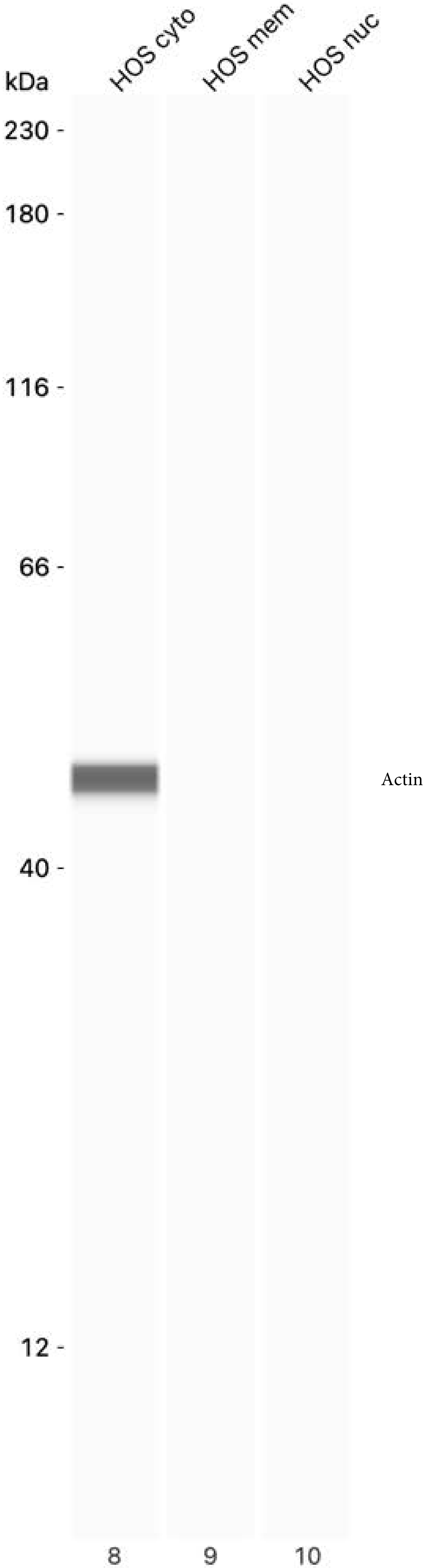
kDa
HOS cyto
HOS mem
HOS nuc
230 -
180 -
116 -
66 -
40 -
12 -
8
9
10
Actin

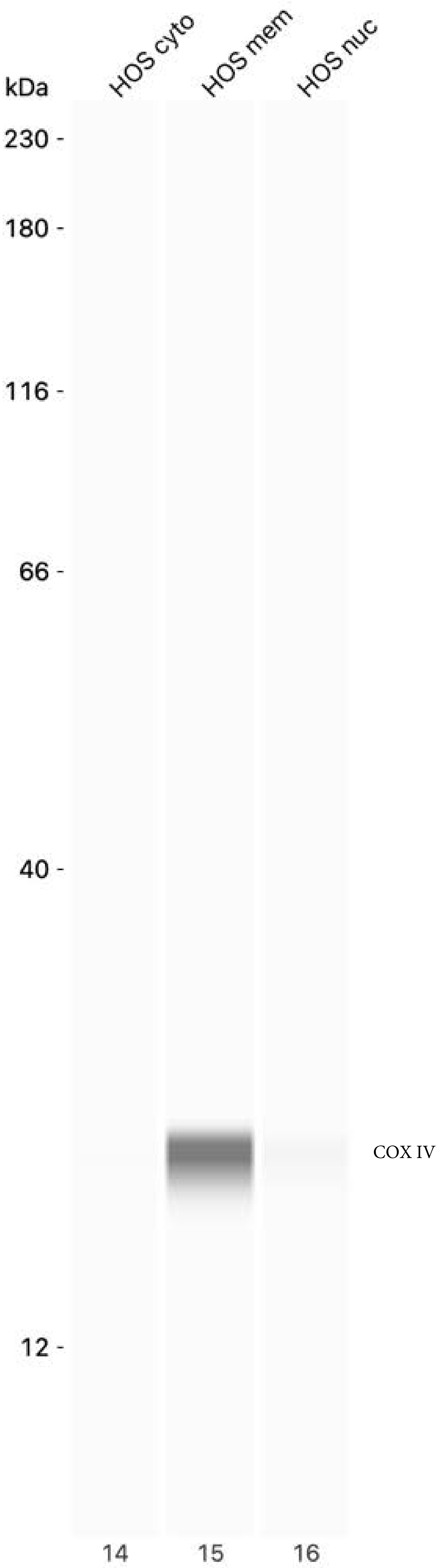

kDa
HOS cyto
HOS mem
HOS nuc
230 -
180 -
116 -
66 -
40 -
12 -
COX IV
14
15
16

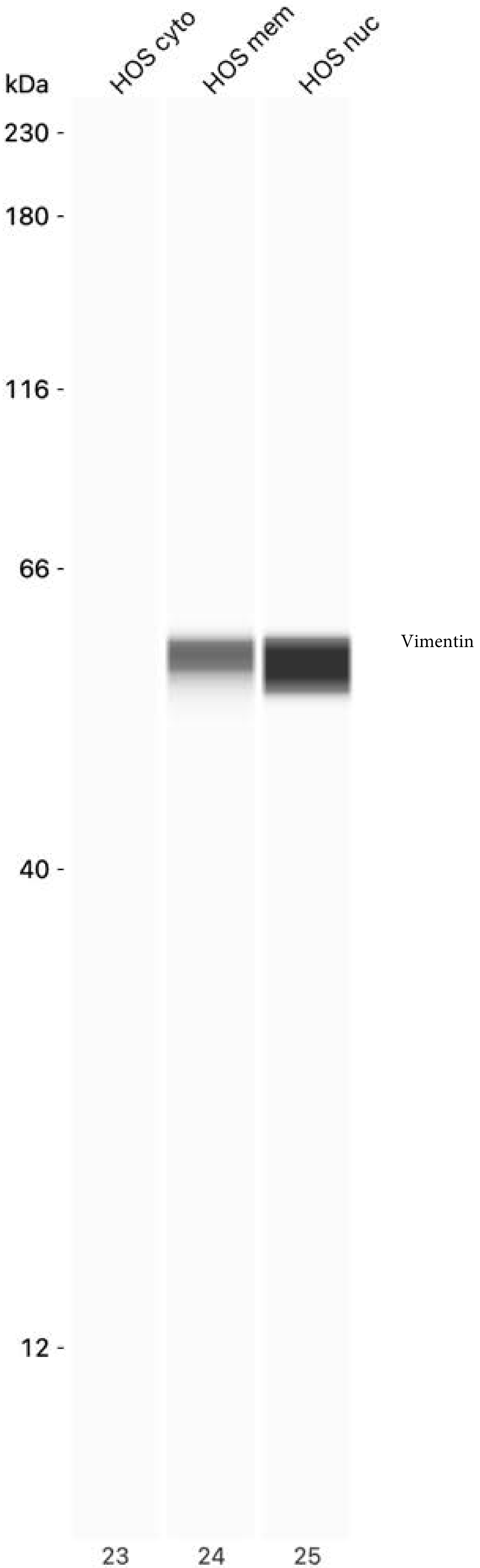

kDa
HOS cyto
HOS mem
HOS nuc
230 -
180 -
116 -
66 -
40 -
12 -
Vimentin
23
24
25

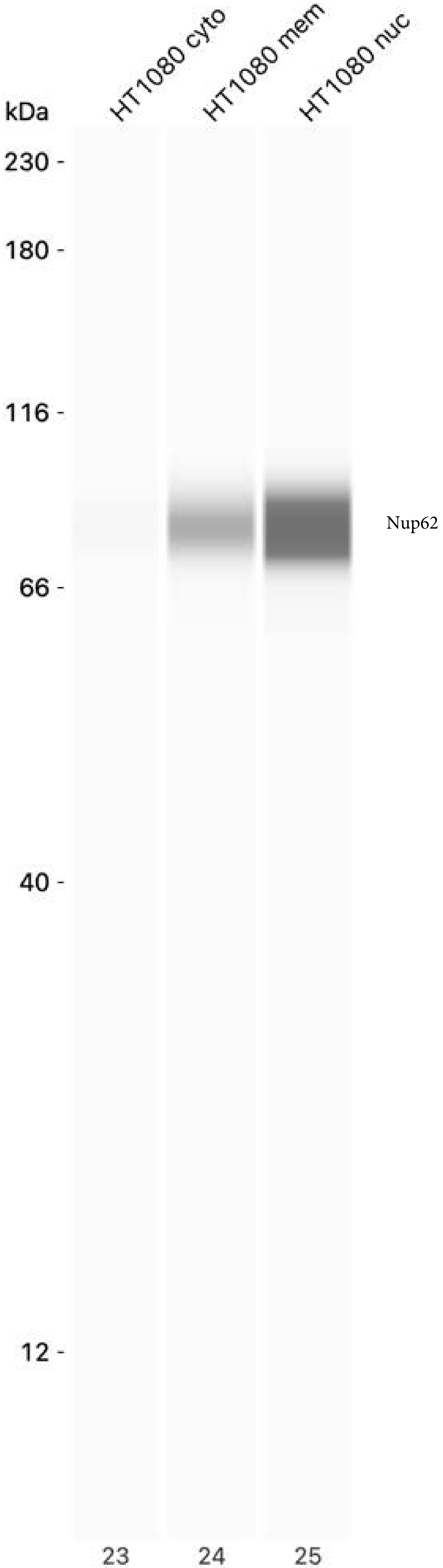
kDa
HT1080 cyto
HT1080 mem
HT1080 nuc
230 -
180 -
116 -
66 -
40 -
12 -
Nup62
23
24
25

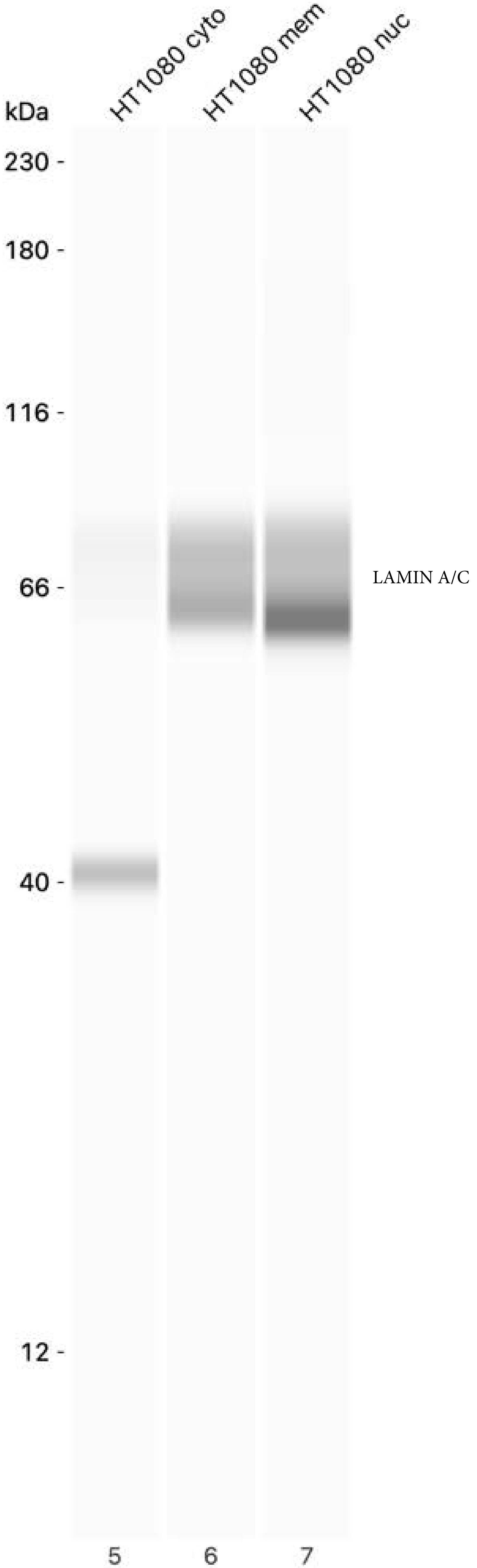
kDa
HT1080 cyto
HT1080 mem
HT1080 nuc
230 -
180 -
116 -
66 -
40 -
12 -
LAMIN A/C
5
6
7

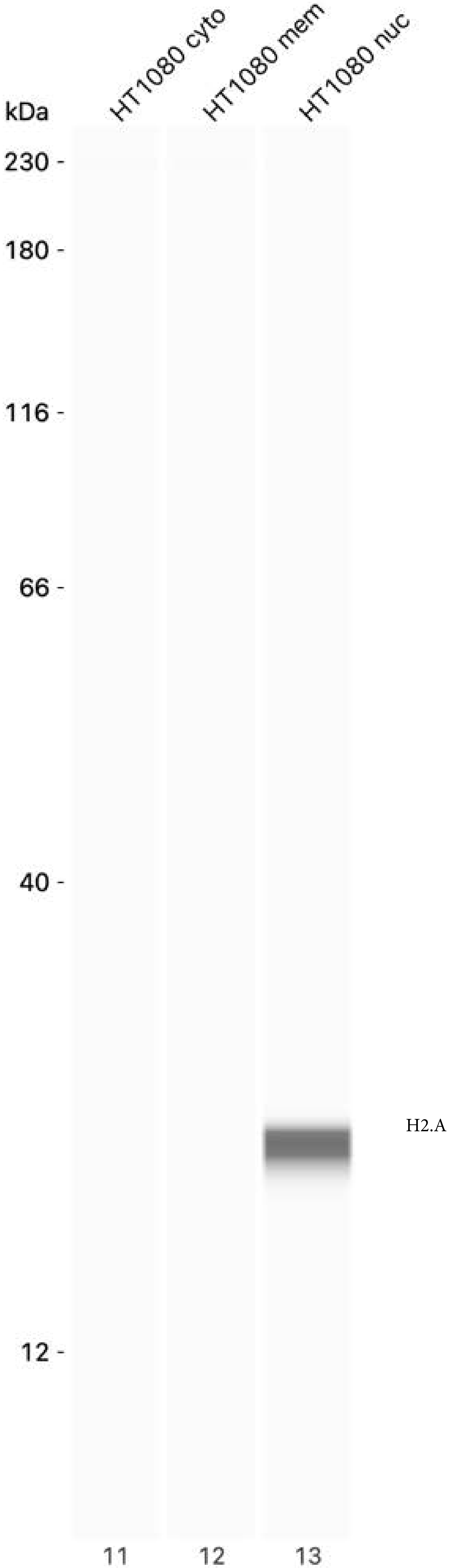

kDa
HT1080 cyto
HT1080 mem
HT1080 nuc
230
180
116
66
40
12
H2.A
11
12
13

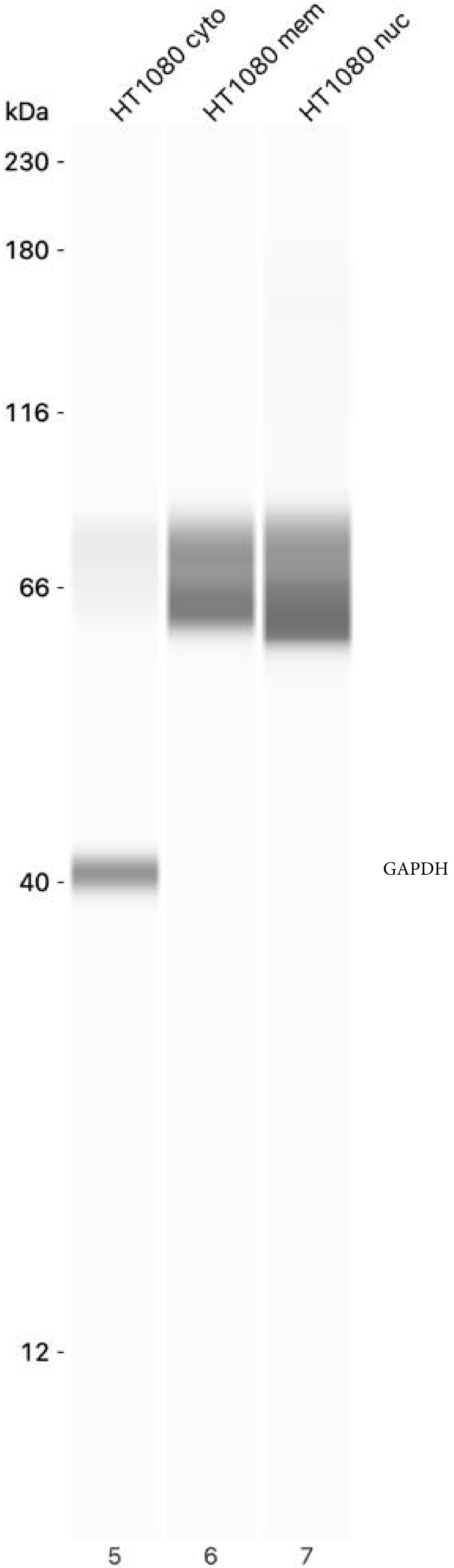
kDa
HT1080 cyto
HT1080 mem
HT1080 nuc
230
180
116
66
40
12
5
6
7
GAPDH

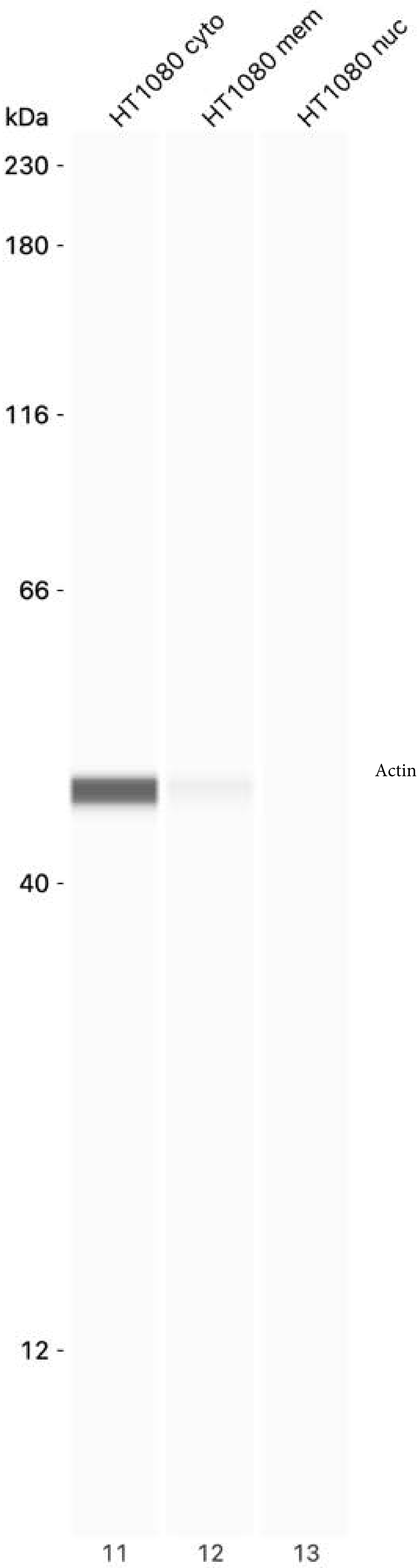

HT1080 cyto
HT1080 mem
HT1080 nuc
kDa
230 -
180 -
116 -
66 -
40 -
12 -
11
12
13

Actin

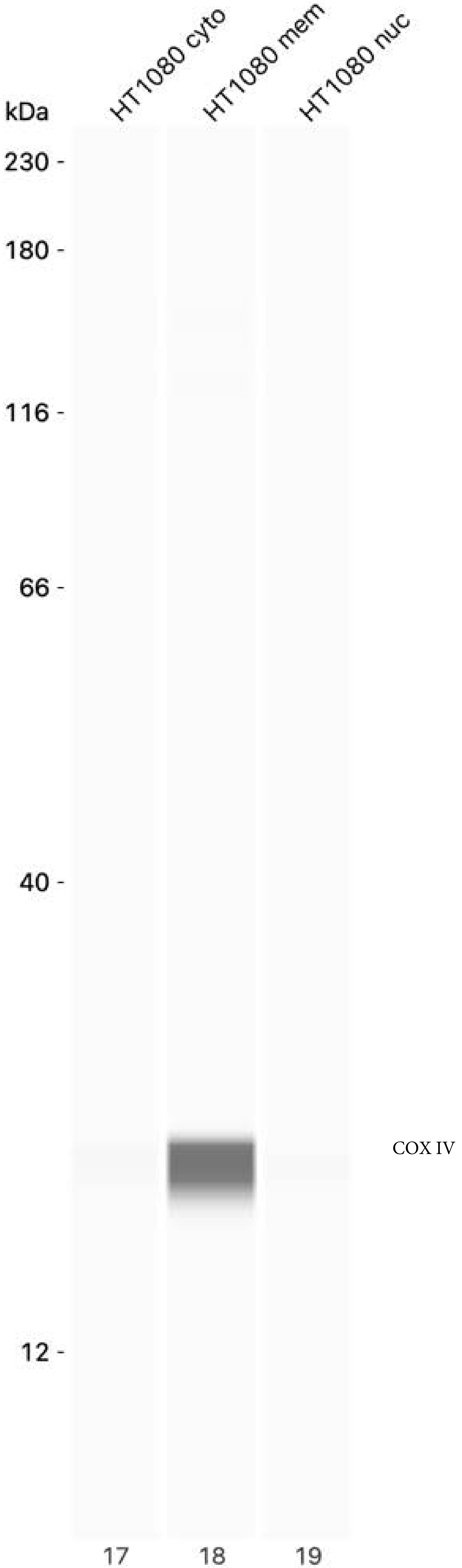
kDa
HT1080 cyto
HT1080 mem
HT1080 nuc
230 -
180 -
116 -
66 -
40 -
12 -
COX IV
17
18
19

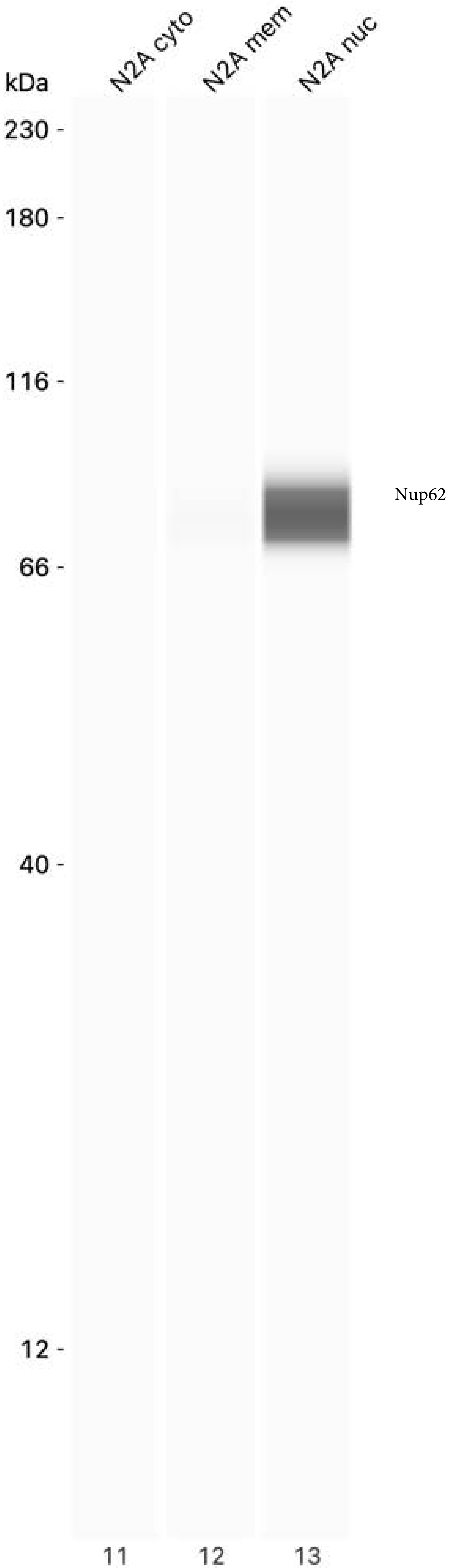
kDa
N2A cyto
N2A mem
N2A nuc
230
180
116
66
40
12
Nup62
11
12
13

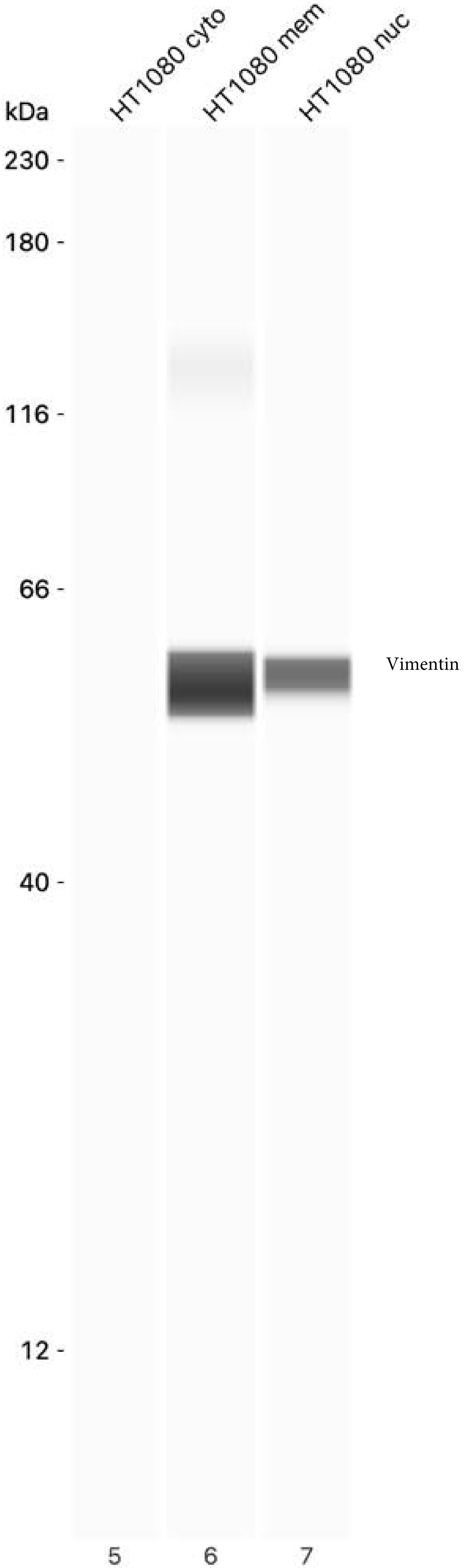
kDa
HT1080 cyto
HT1080 mem
HT1080 nuc
230
180
116
66
40
12
Vimentin
5
6
7

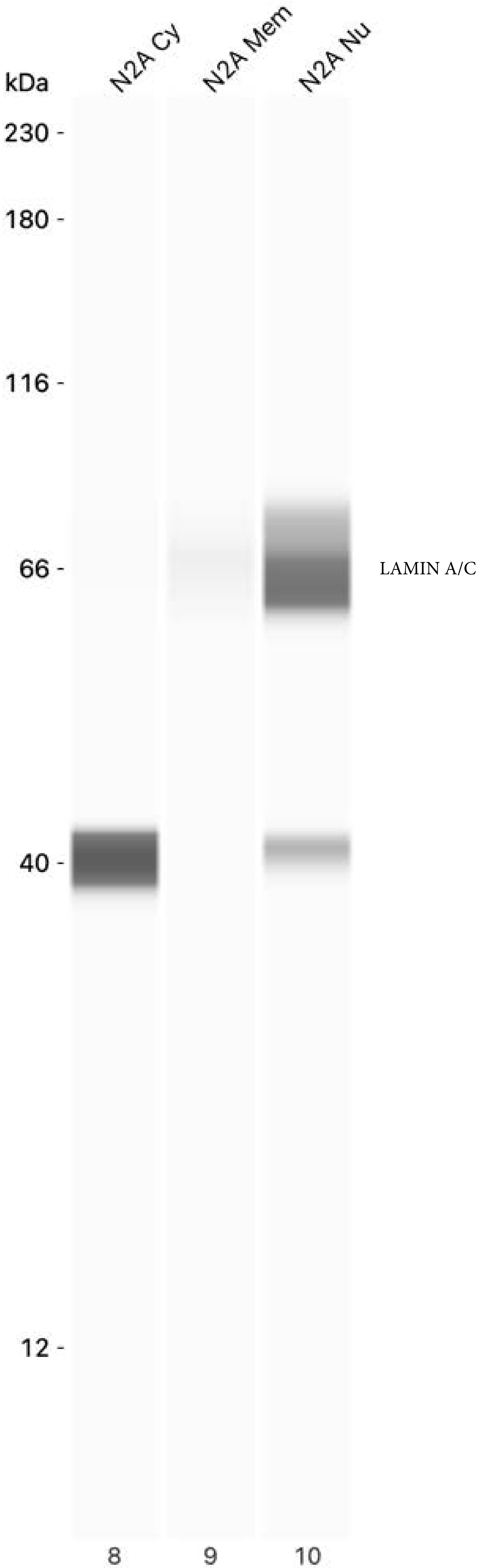

kDa
N2A Cy
N2A Mem
N2A Nu
230 -
180 -
116 -
66 -
40 -
12 -
LAMIN A/C
8
9
10

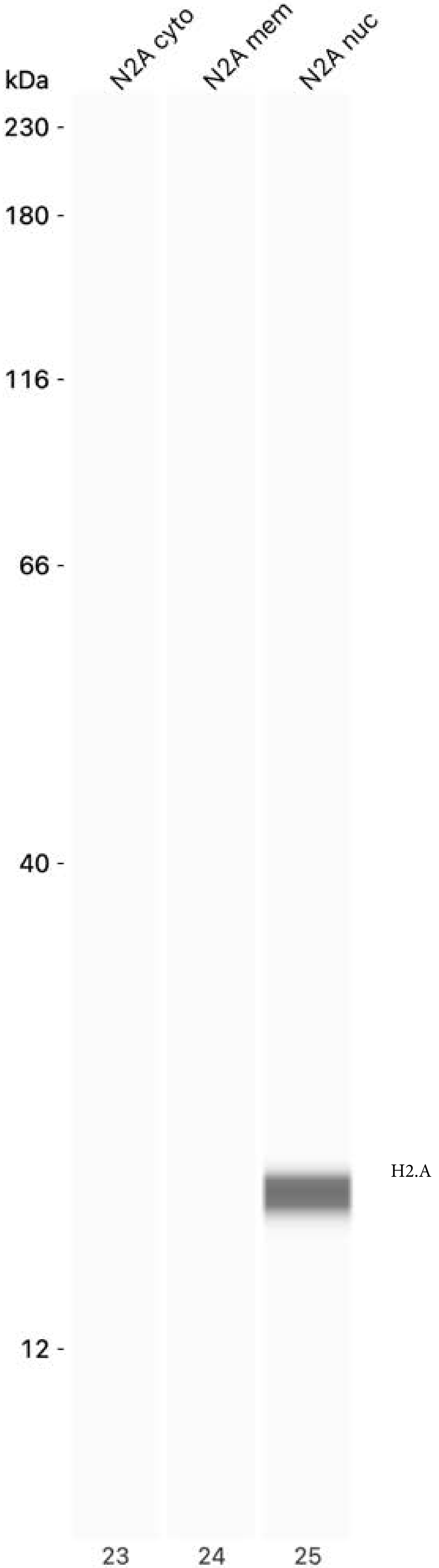

kDa
N2A cyto
N2A mem
N2A nuc
230 -
180 -
116 -
66 -
40 -
12 -
H2.A
23
24
25

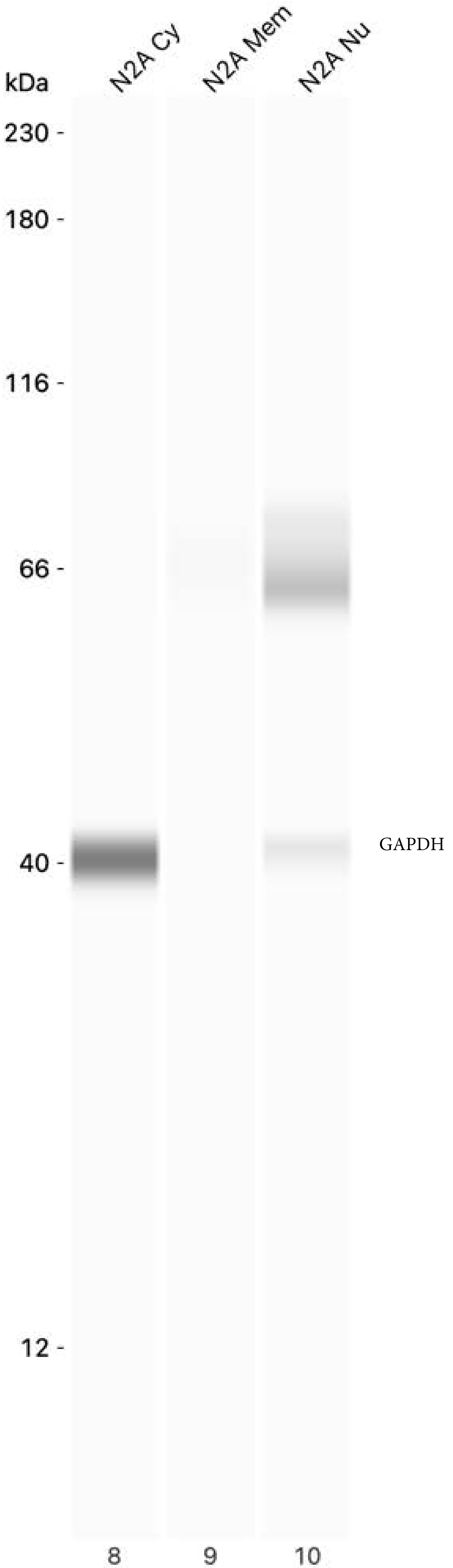
kDa
N2A Cy
N2A Mem
N2A Nu
230
180
116
66
40
12
GAPDH
8
9
10

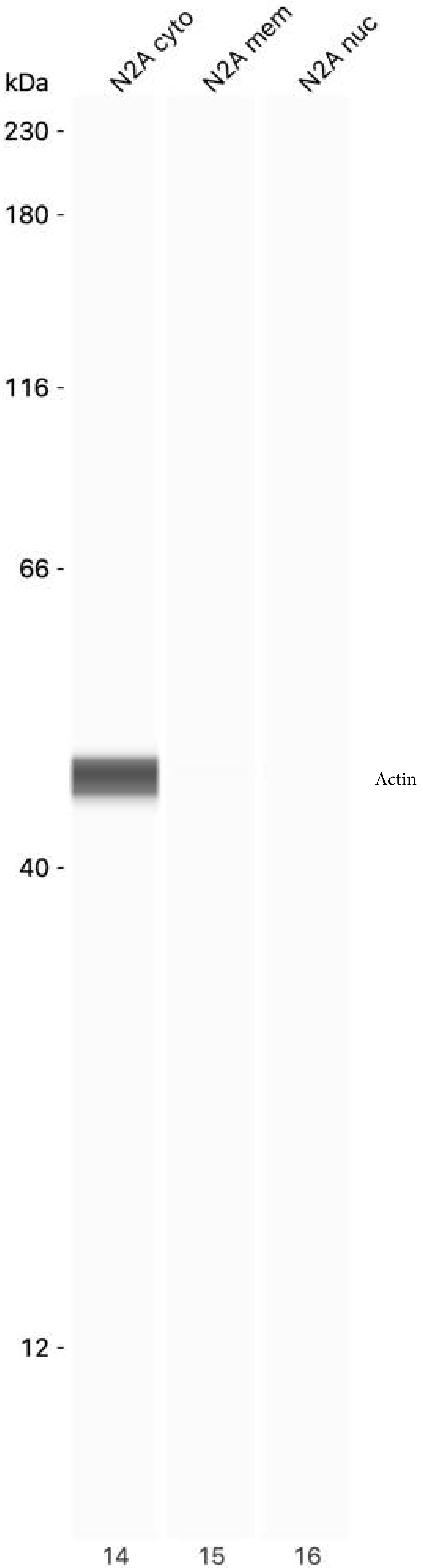
kDa
N2A cyto
N2A mem
N2A nuc
230 -
180 -
116 -
66 -
40 -
12 -
Actin
14
15
16

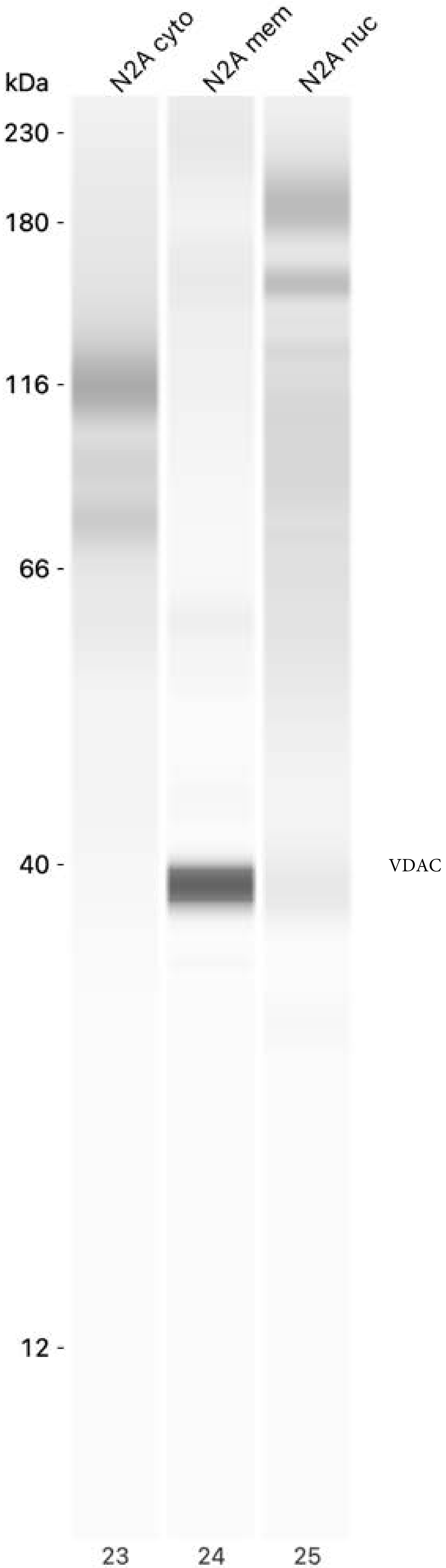

kDa
N2A cyto
N2A mem
N2A nuc
230
180
116
66
40
12
23
24
25
VDAC

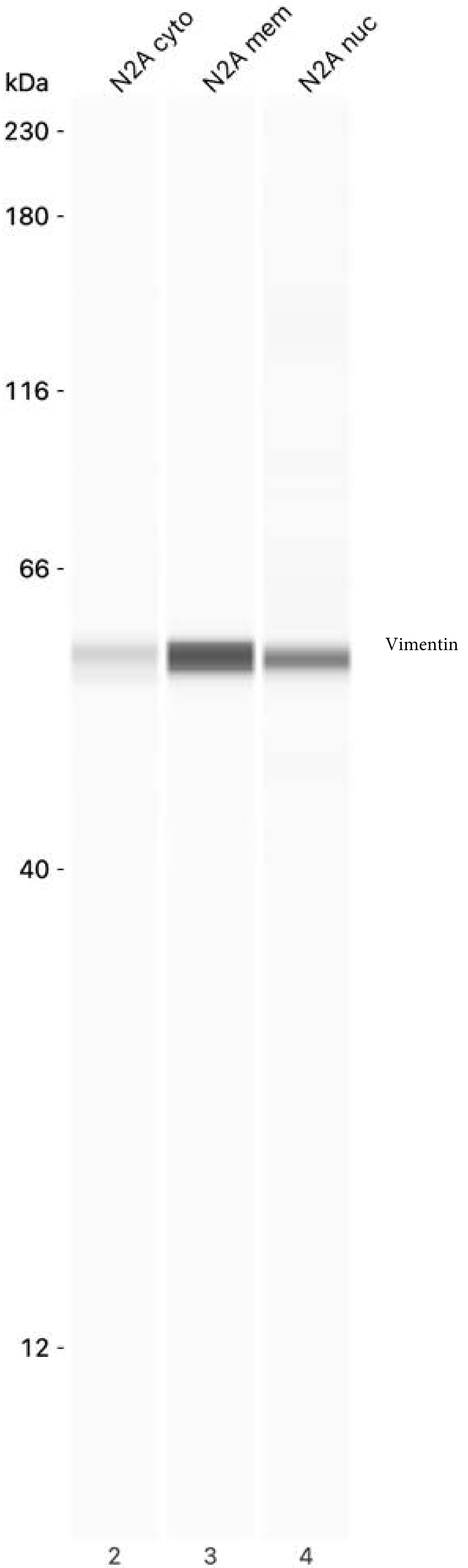

N2A cyto
N2A mem
N2A nuc
kDa
230
180
116
66
40
12
Vimentin
2
3
4

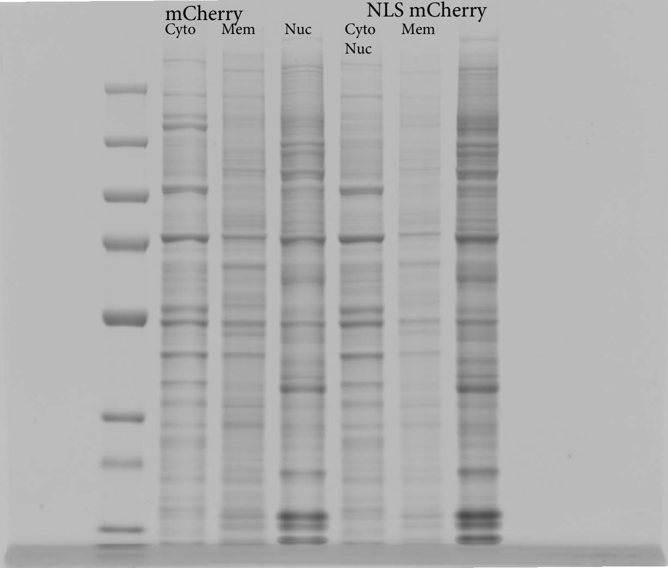
mCherry
NLS mCherry
Cyto
Mem
Nuc
Cyto
Nuc
Mem

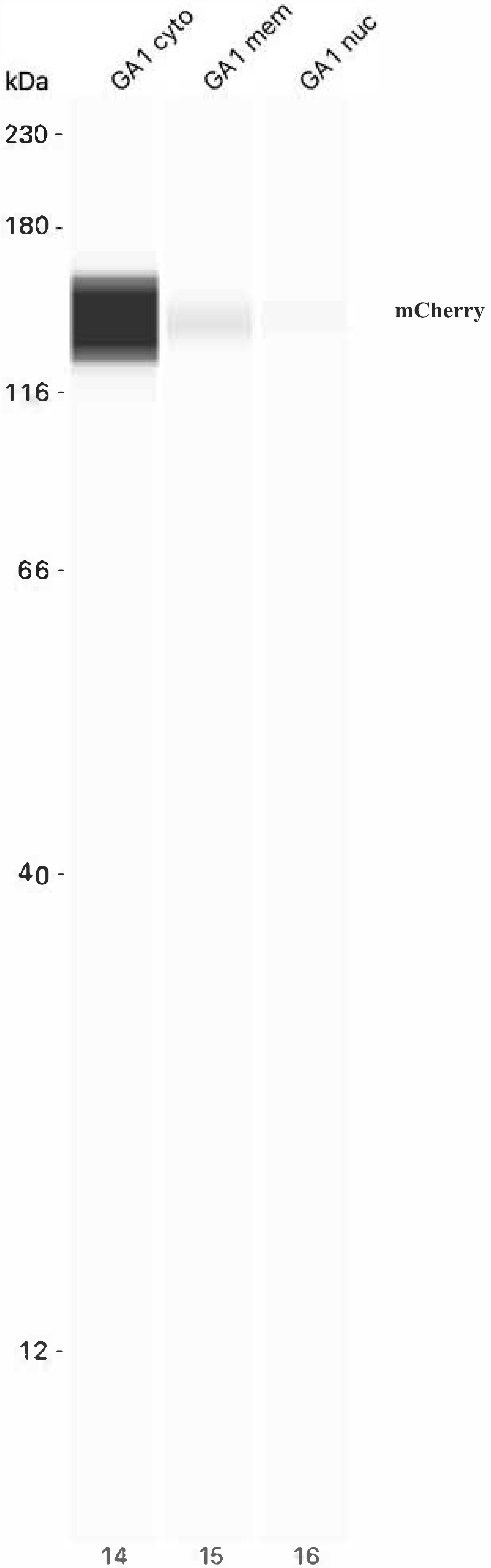
kDa
GA1 cyto
GA1 mem
GA1 nuc
230
180
116
66
40
12
mCherry
14
15
16

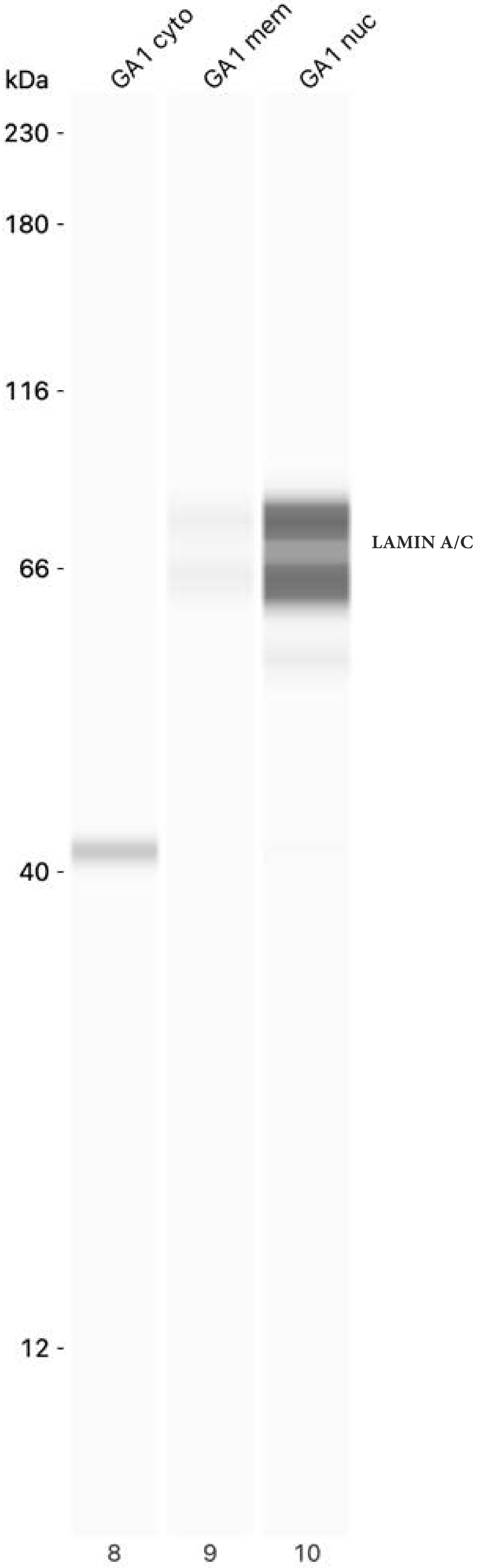

kDa
GA1 cyto
GA1 mem
GA1 nuc
230
180
116
66
40
12
LAMIN A/C
8
9
10

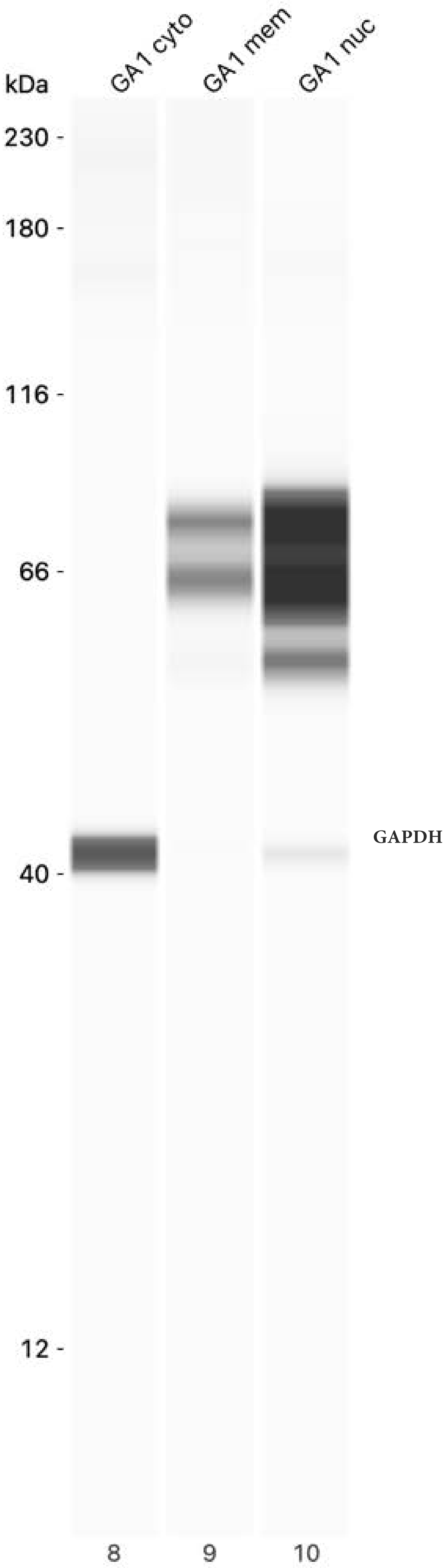
kDa
GA1 cyto
GA1 mem
GA1 nuc
230
180
116
66
40
12
GAPDH
8
9
10

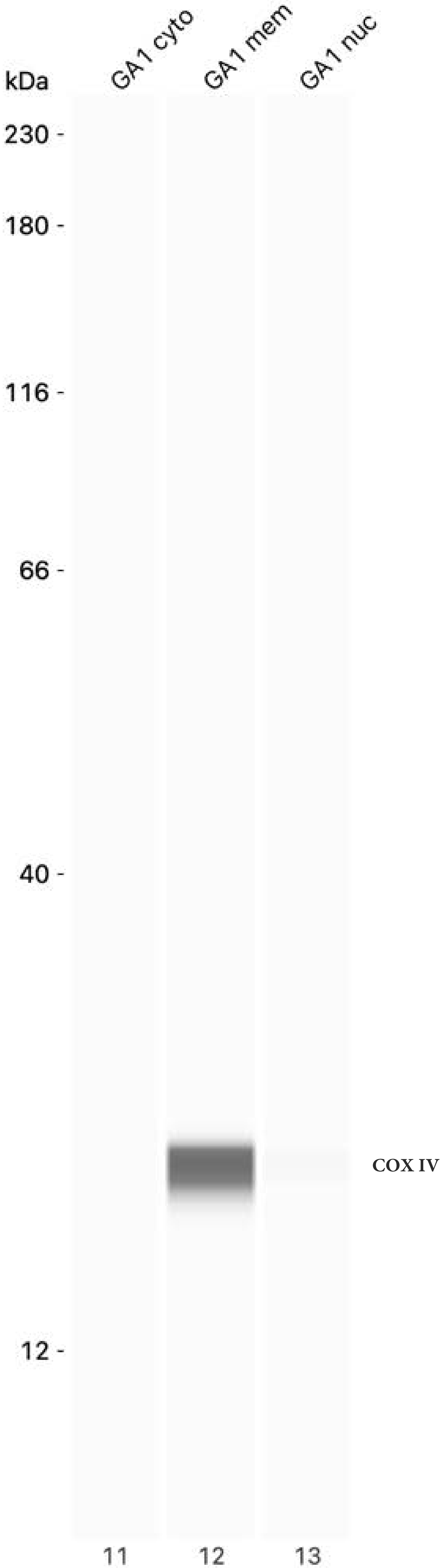

kDa
GA1 cyto
GA1 mem
GA1 nuc
230
180
116
66
40
12
COX IV
11
12
13

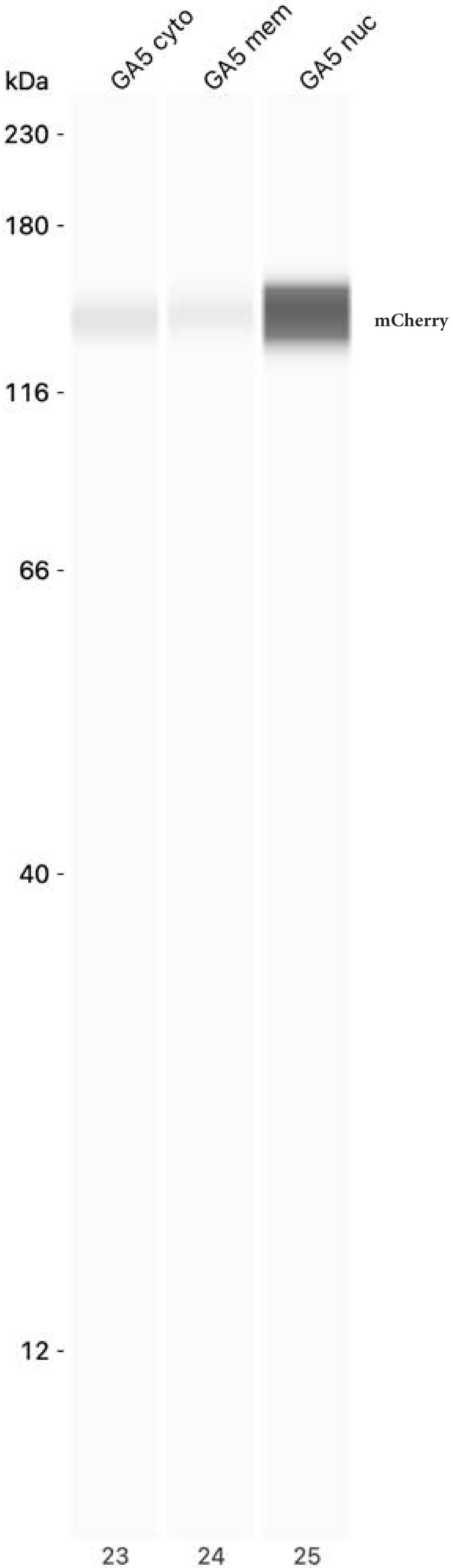
kDa
GA5 cyto
GA5 mem
GA5 nuc
230
180
116
66
40
12
mCherry
23
24
25

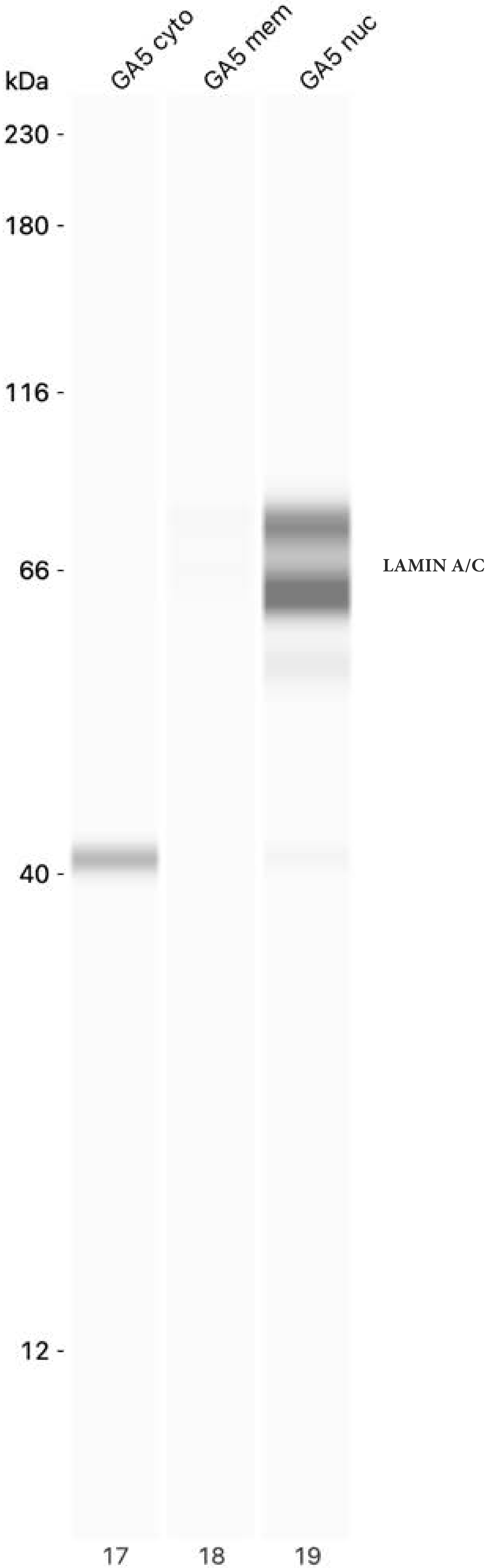

kDa
GA5 cyto
GA5 mem
GA5 nuc
230 -
180 -
116 -
66 -
40 -
12 -
LAMIN A/C
17
18
19

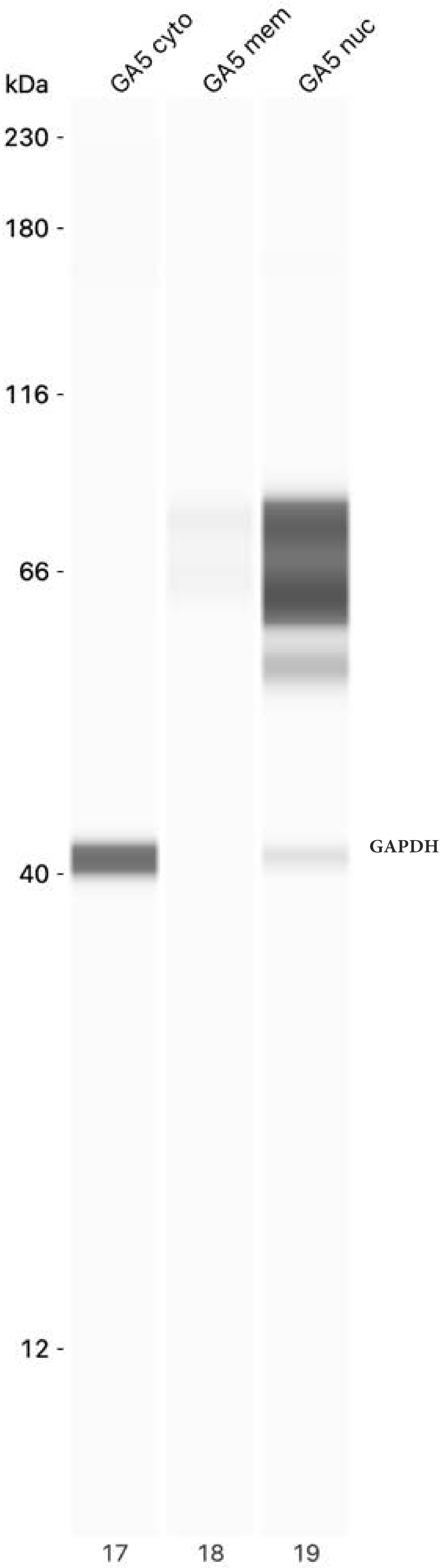

kDa
GA5 cyto
GA5 mem
GA5 nuc
230 -
180 -
116 -
66 -
40 -
12 -
GAPDH
17
18
19

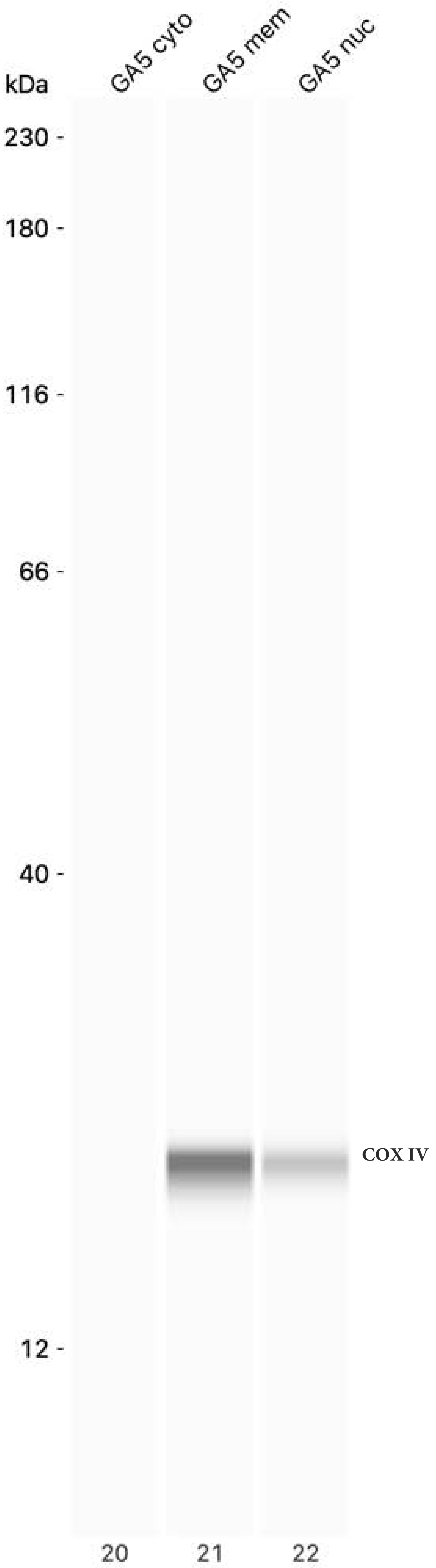
kDa
GA5 cyto
GA5 mem
GA5 nuc
230
180
116
66
40
12
COX IV
20
21
22
