# Supplementary material for: A general method for quantitative fractionation of mammalian cells
Source: J Cell Biol. 2023 Mar 15;222(6):e202209062. doi: 10.1083/jcb.202209062 (PMC10040634; doi:10.1083/jcb.202209062)
Supplement: SourceData F3 — is the source file for Fig. 3. [file JCB_202209062_SourceDataF3.pdf]

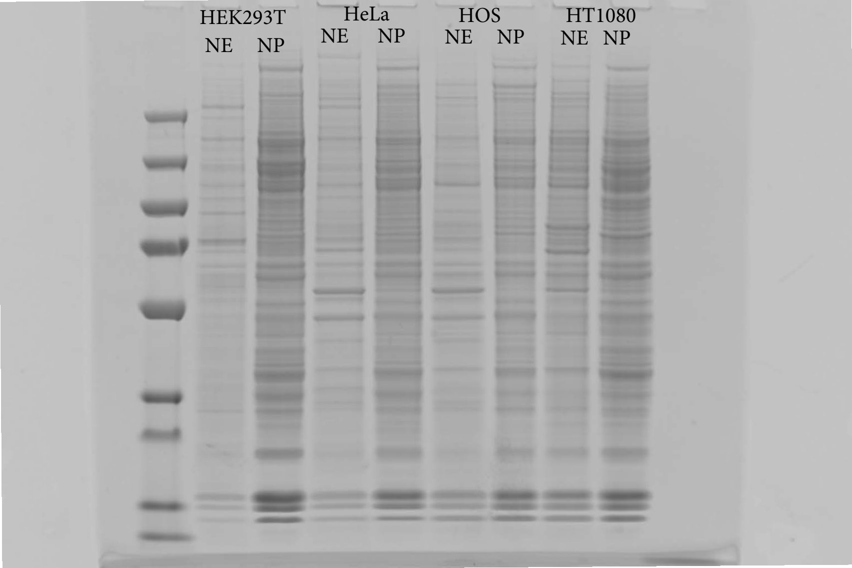

HEK293T
NE
NP
HeLa
NE
NP
HOS
NE
NP
HT1080
NE
NP

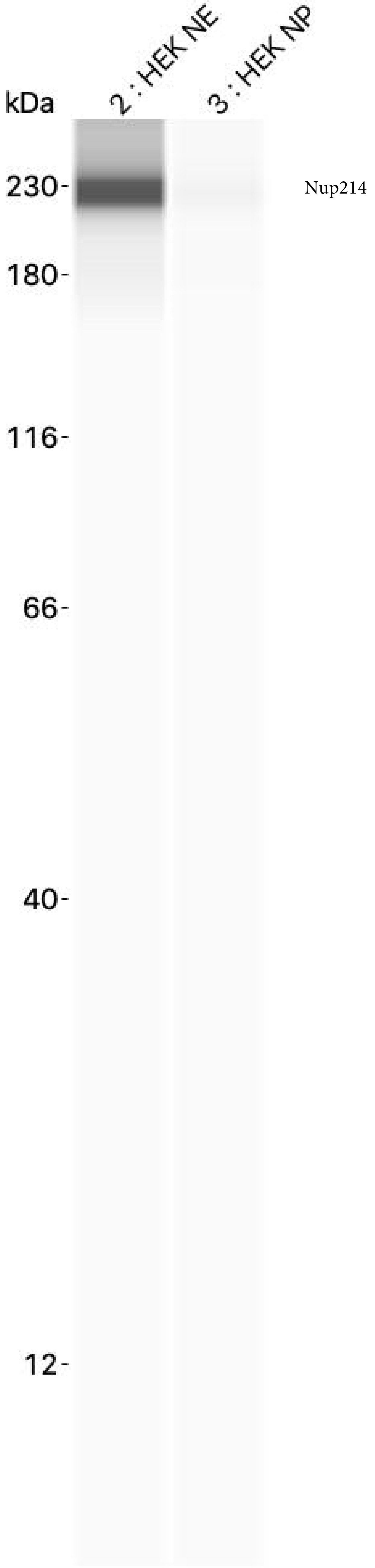

kDa
2 : HEK NE
3 : HEK NP
230
180
116
66
40
12
Nup214

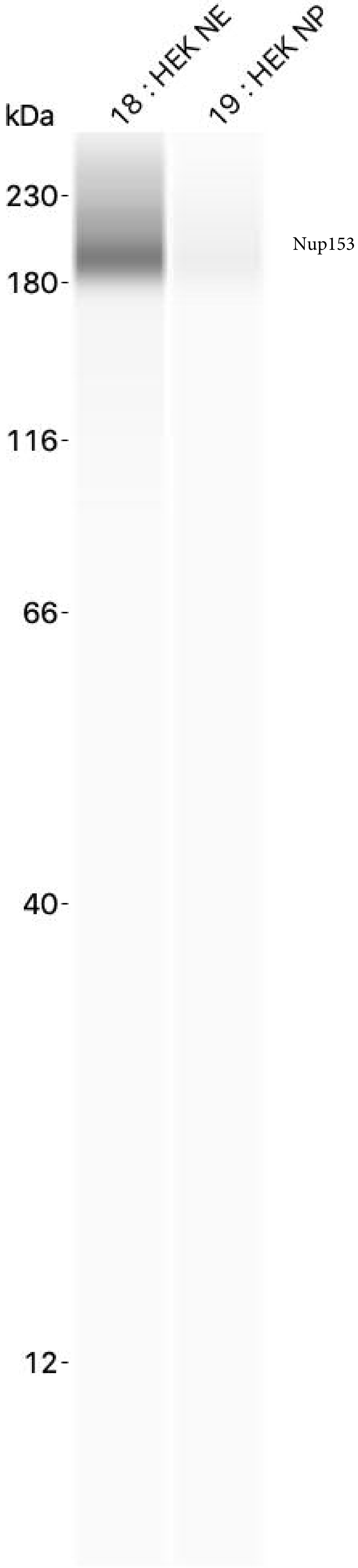

18 : HEK NE
19 : HEK NP
kDa
230
180
116
66
40
12
Nup153

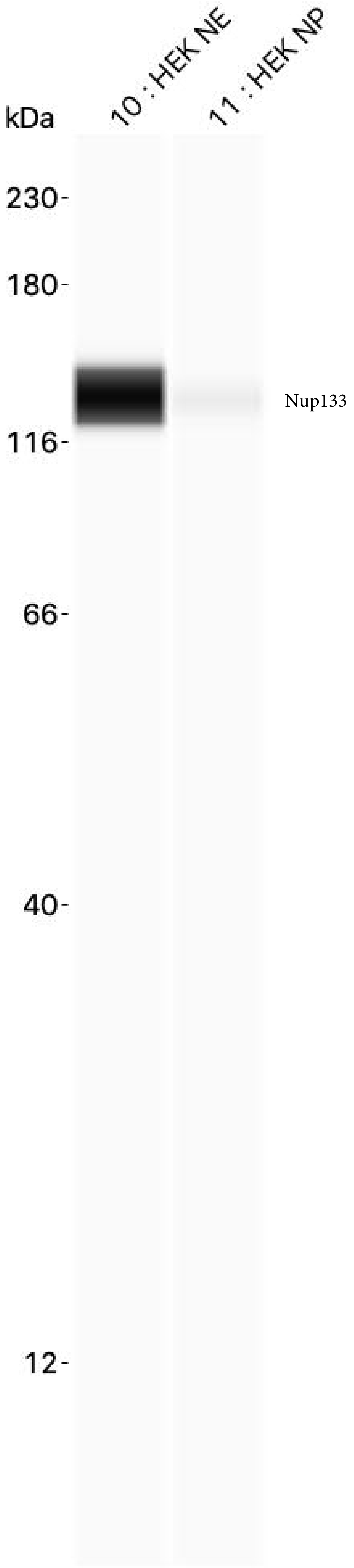
kDa
10 : HEK NE
11 : HEK NP
230
180
116
66
40
12
Nup133

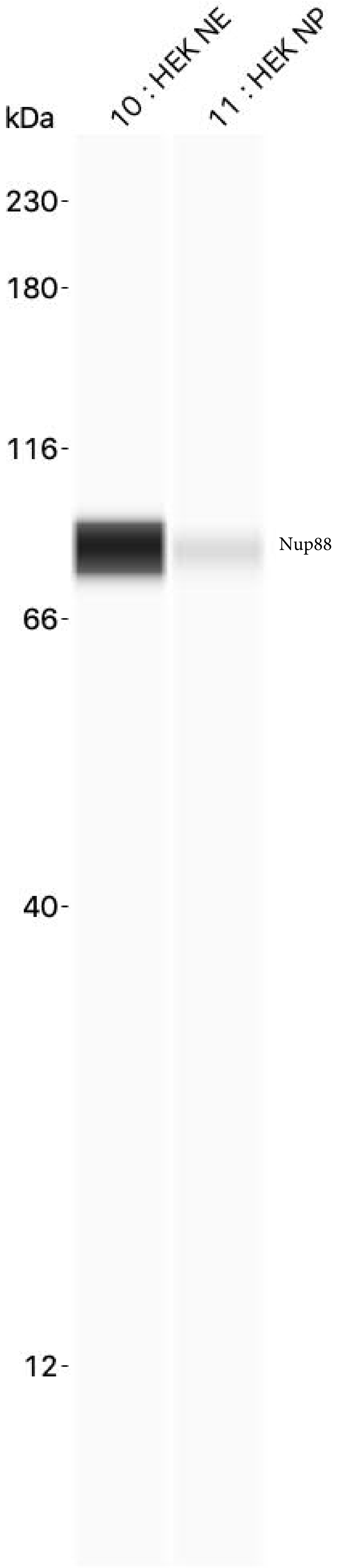

kDa
10 : HEK NE
11 : HEK NP
230
180
116
66
40
12
Nup88

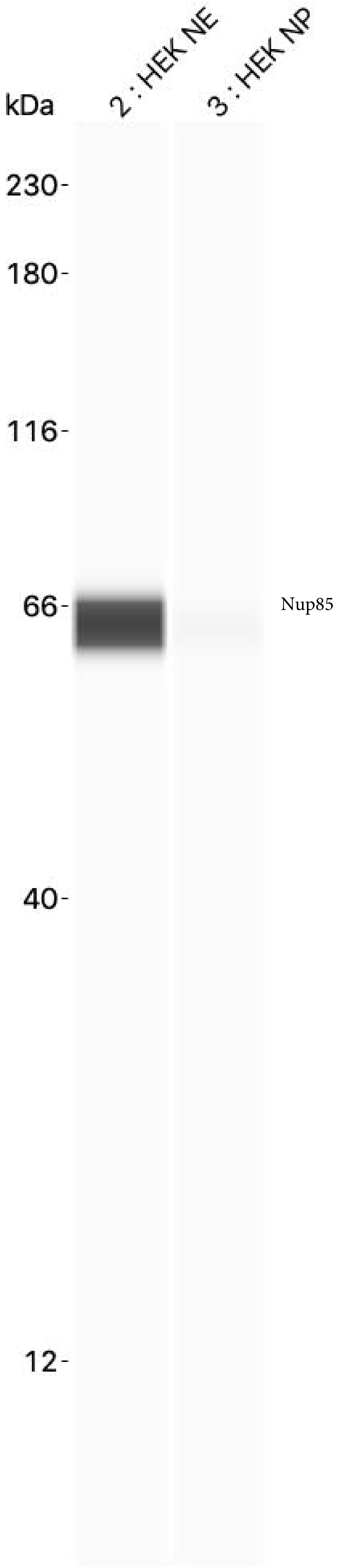
kDa
2 : HEK NE
3 : HEK NP
230
180
116
66
40
12
Nup85

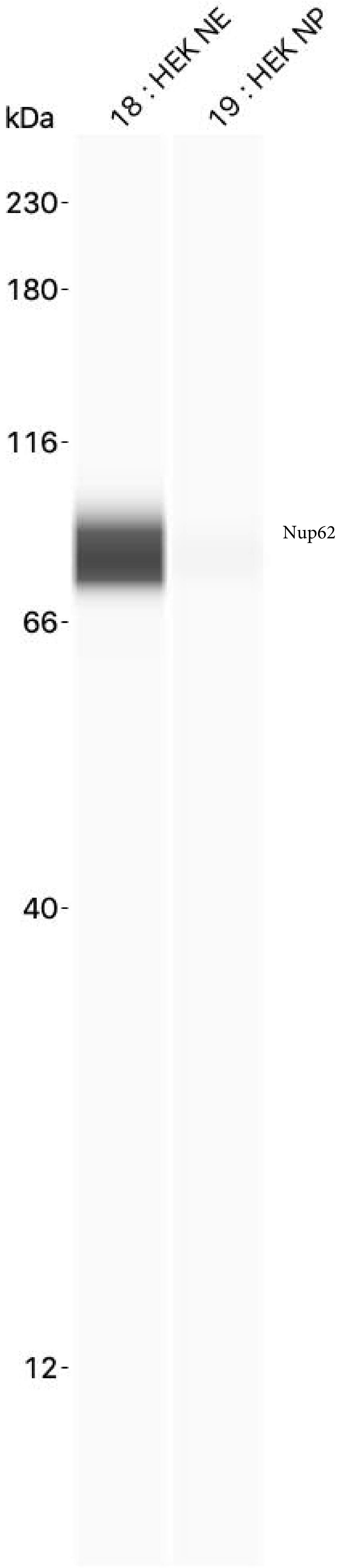
18 : HEK NE
19 : HEK NP
kDa
230
180
116
66
40
12
Nup62

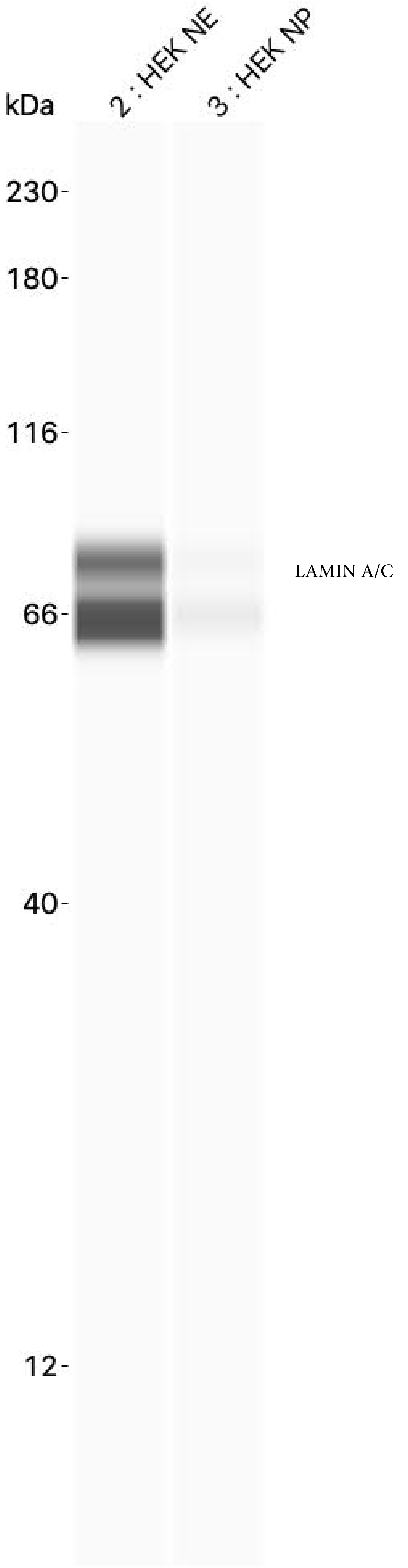
kDa
2 : HEK NE
3 : HEK NP
230
180
116
66
40
12
LAMIN A/C

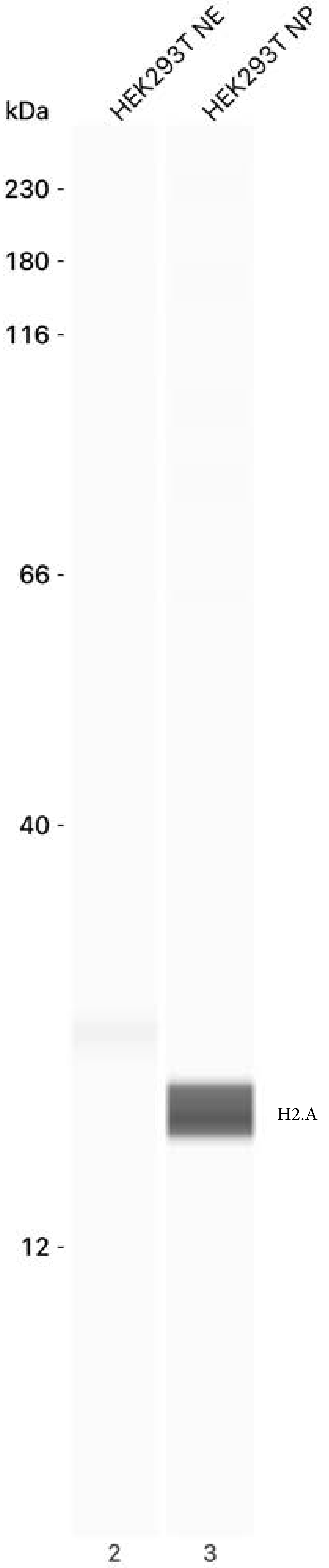
kDa
HEK293T NE
HEK293T NP
230 -
180 -
116 -
66 -
40 -
12 -
H2.A
2
3

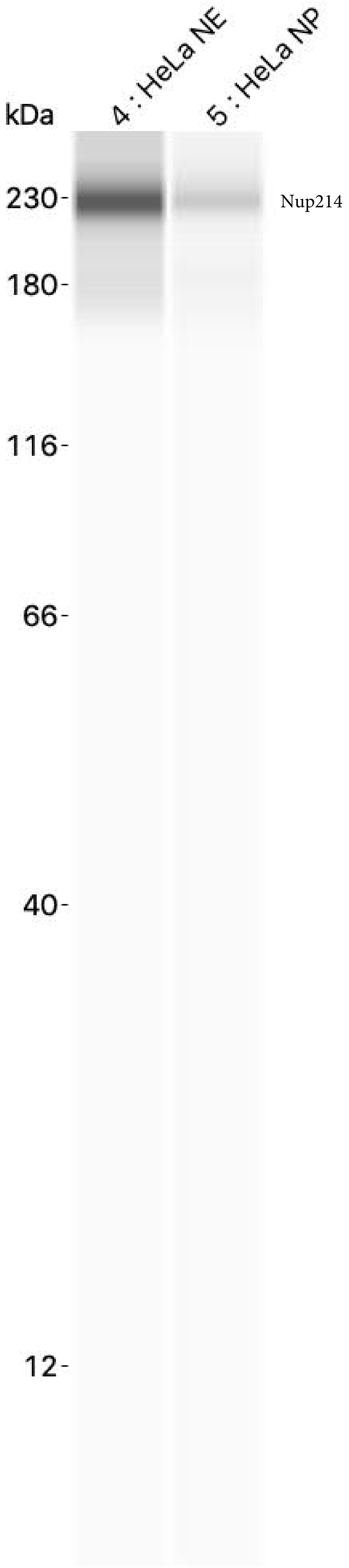

kDa
4 : HeLa NE
5 : HeLa NP
230
180
116
66
40
12
Nup214

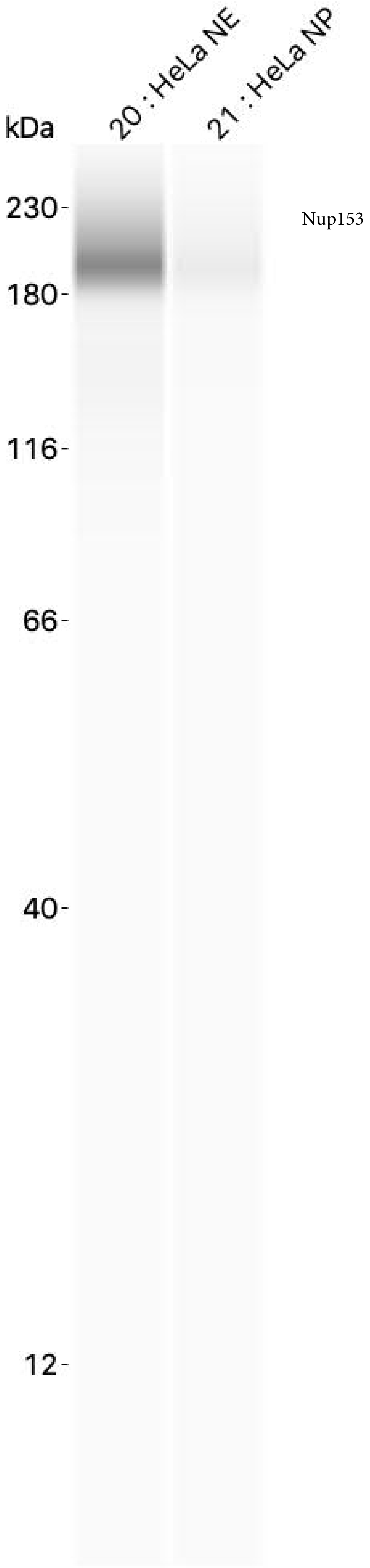
kDa
20 : HeLa NE
21 : HeLa NP
230
180
116
66
40
12
Nup153

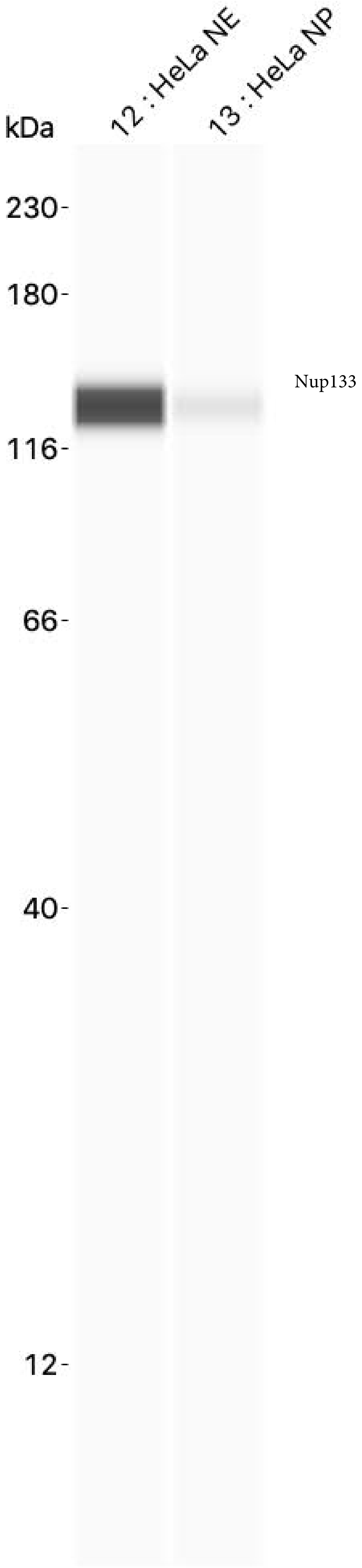
kDa
12 : HeLa NE
13 : HeLa NP
230
180
116
66
40
12
Nup133

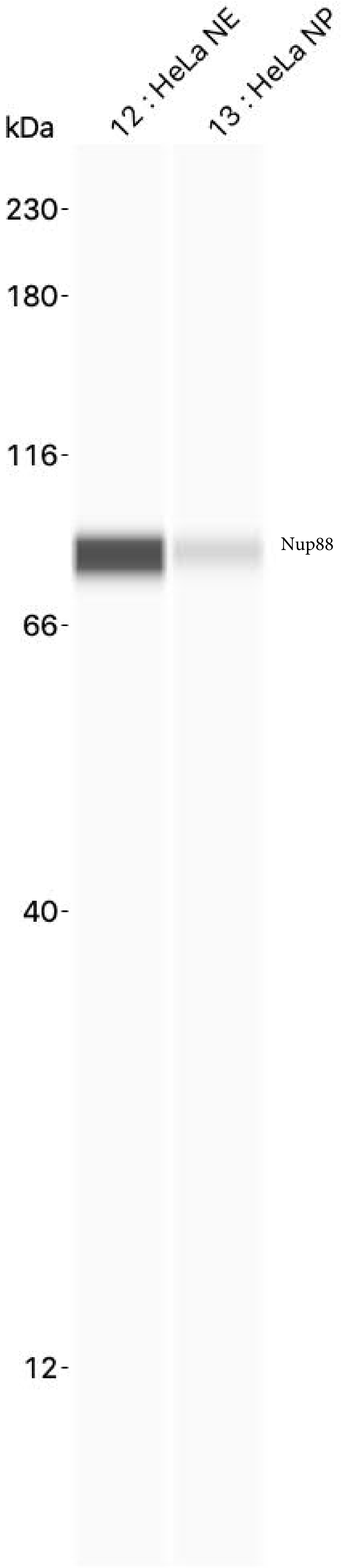

kDa
12 : HeLa NE
13 : HeLa NP
230
180
116
66
40
12
Nup88

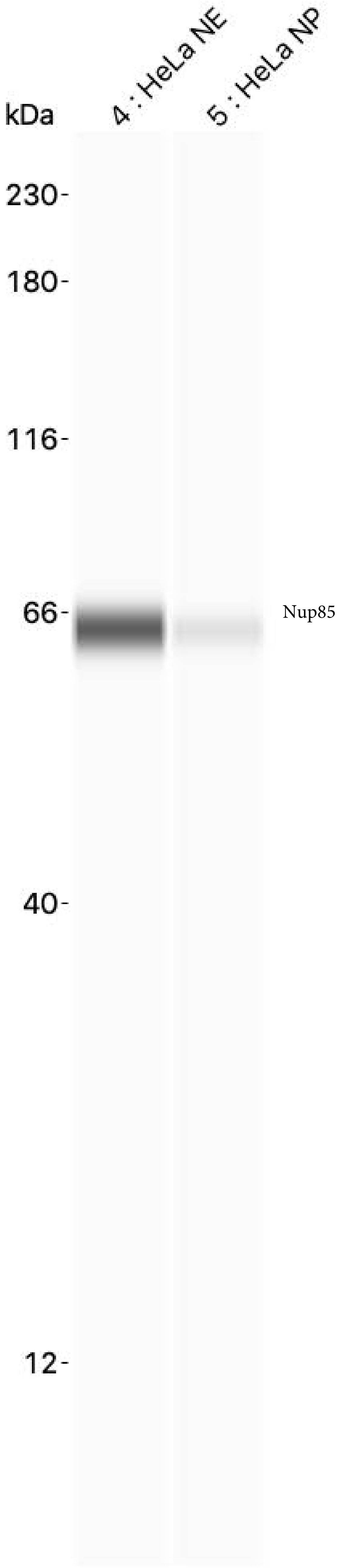

kDa
4 : HeLa NE
5 : HeLa NP
230
180
116
66
40
12
Nup85

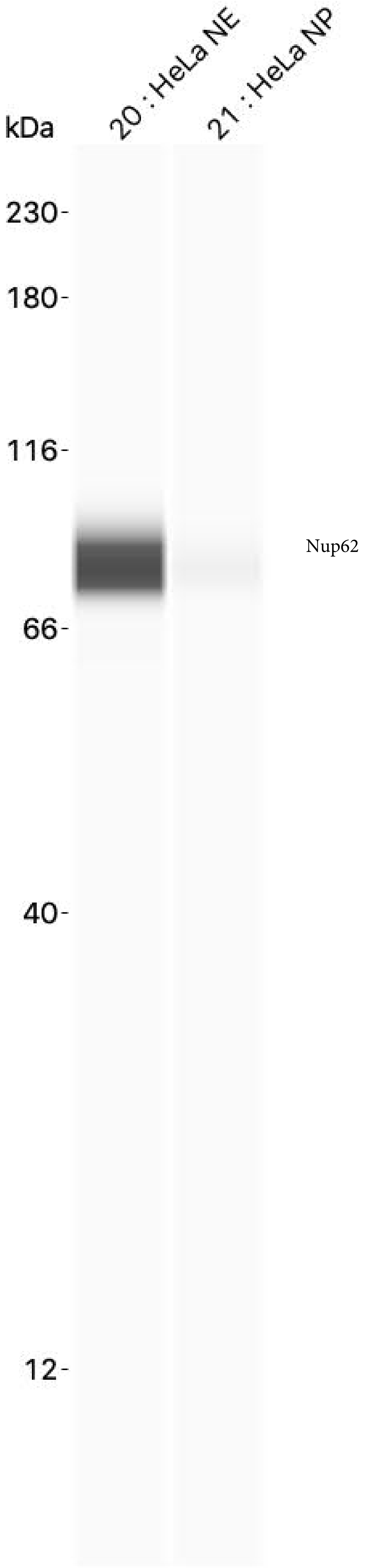

kDa
20 : HeLa NE
21 : HeLa NP
230
180
116
66
40
12
Nup62

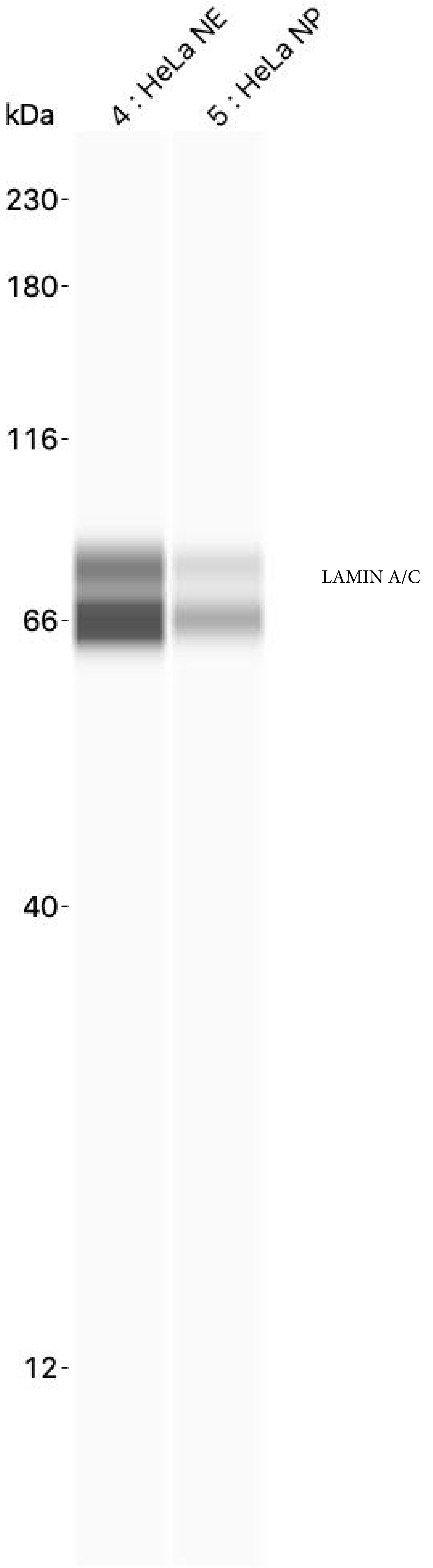
kDa
4 : HeLa NE
5 : HeLa NP
230
180
116
66
40
12
LAMIN A/C

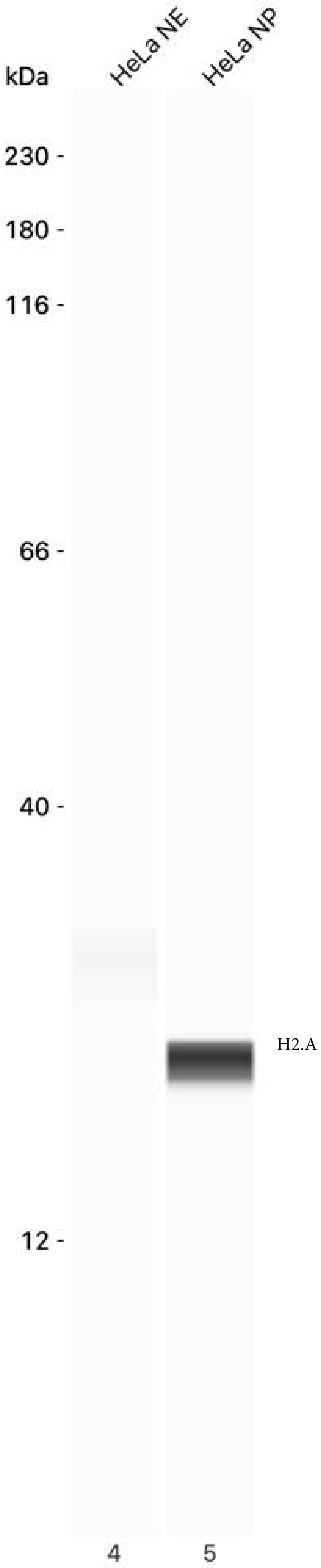
kDa
HeLa NE
HeLa NP
230 -
180 -
116 -
66 -
40 -
12 -
H2.A
4
5

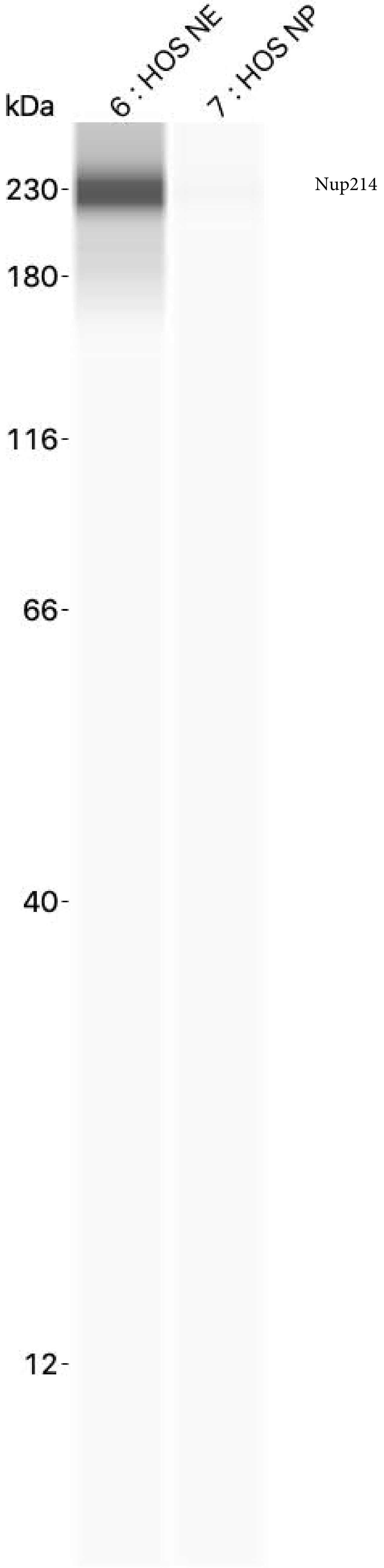

kDa
6 : HOS NE
7 : HOS NP
230
180
116
66
40
12
Nup214

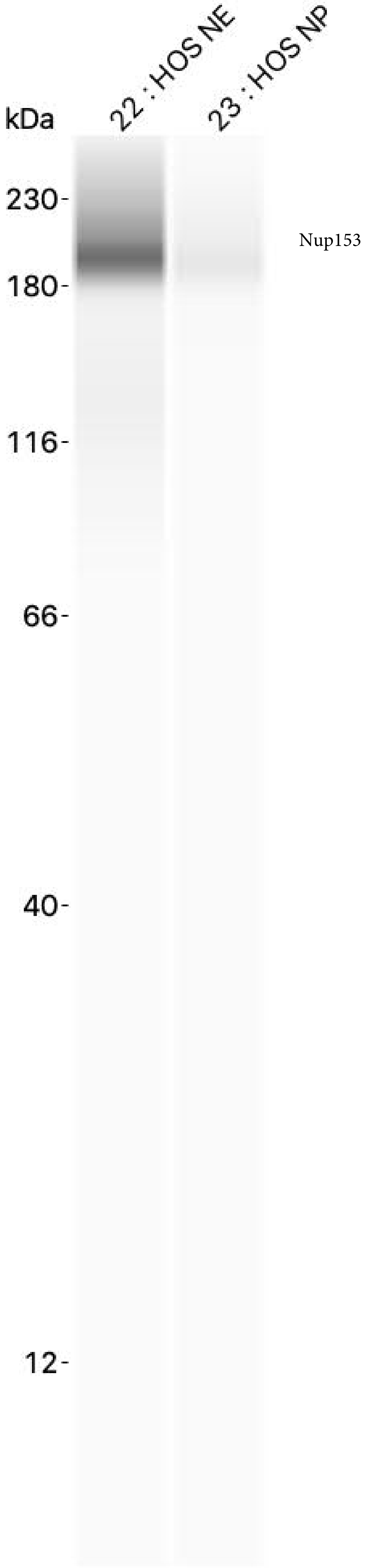
kDa
22 : HOS NE
23 : HOS NP
230
180
116
66
40
12
Nup153

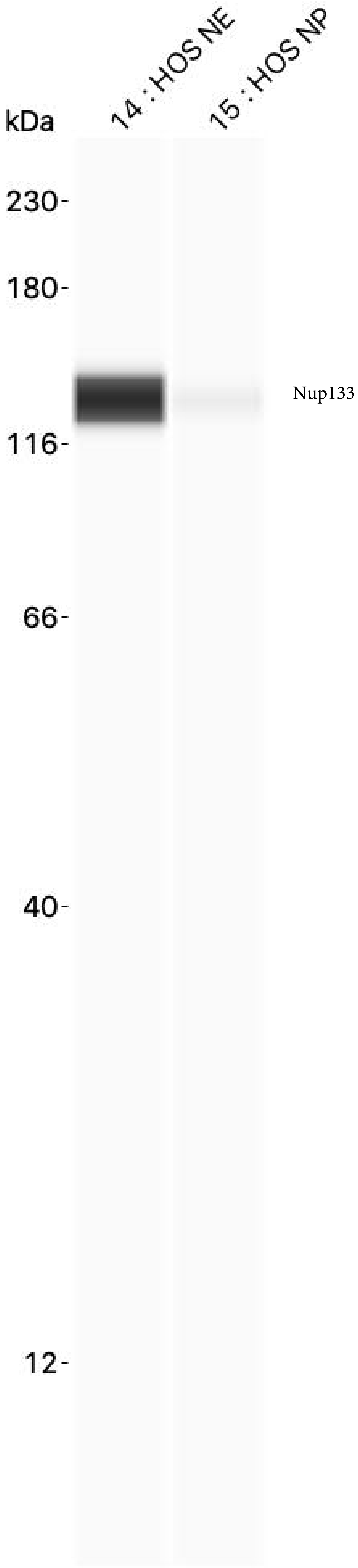

kDa
14 : HOS NE
15 : HOS NP
230
180
116
66
40
12
Nup133

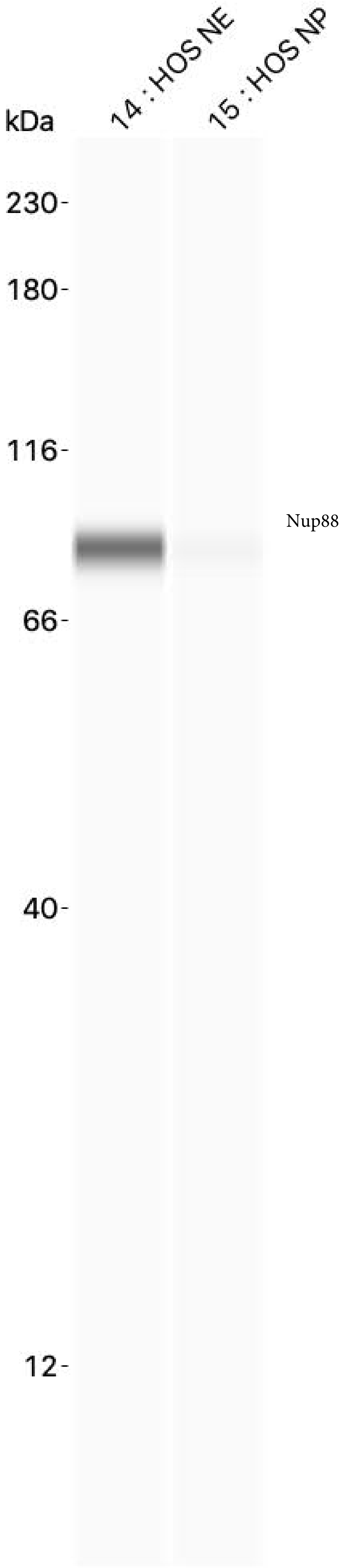

14 : HOS NE
15 : HOS NP
kDa
230
180
116
66
40
12
Nup88

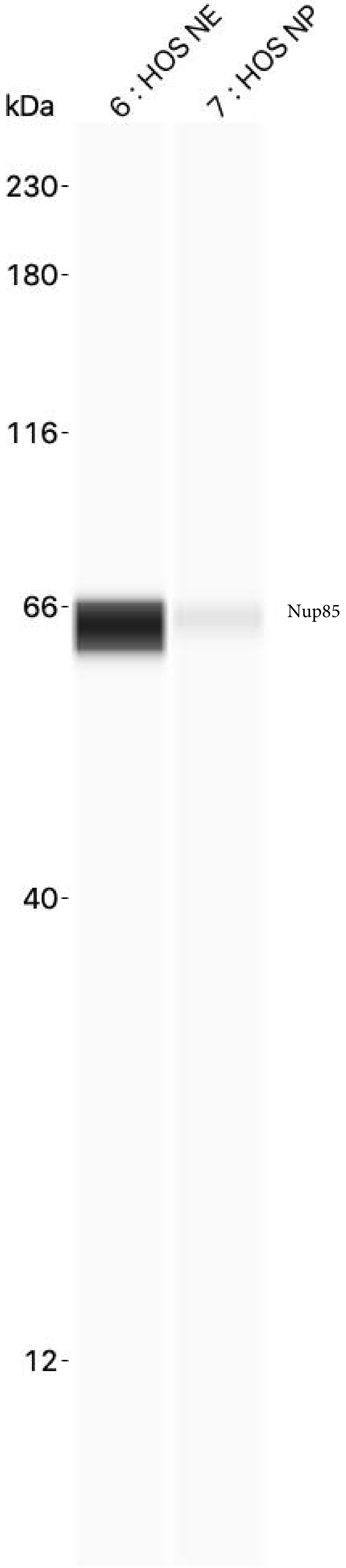

kDa
6 : HOS NE
7 : HOS NP
230-
180-
116-
66-
40-
12-
Nup85

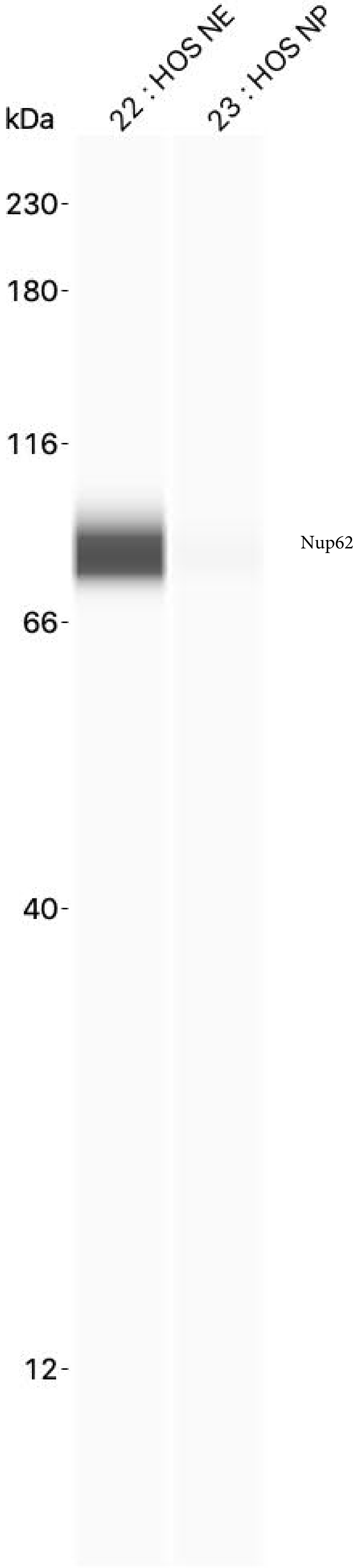
kDa
22 : HOS NE
23 : HOS NP
230
180
116
66
40
12
Nup62

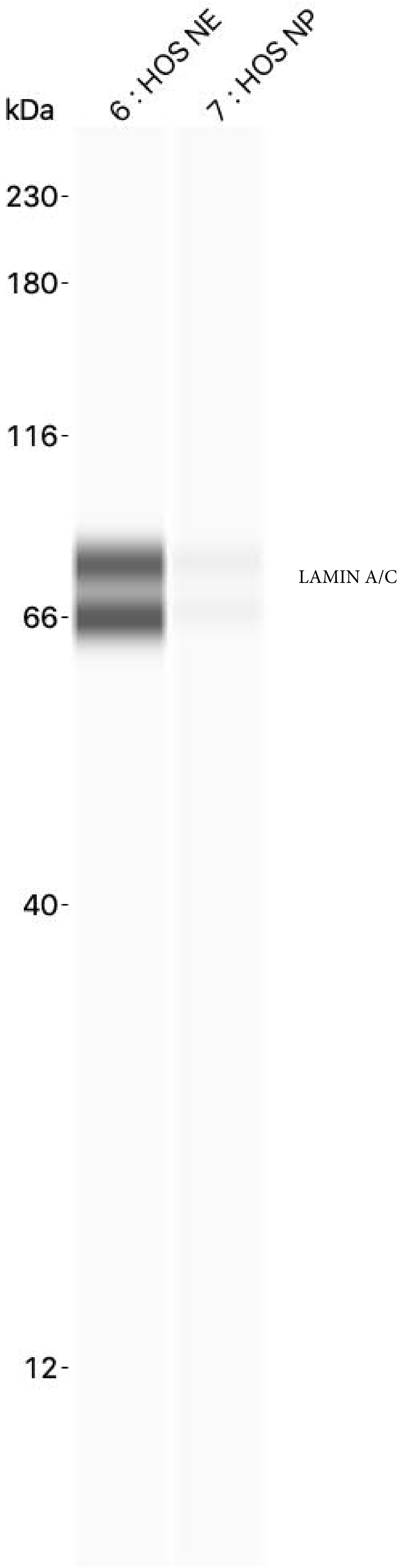
kDa
6 : HOS NE
7 : HOS NP
230
180
116
66
40
12
LAMIN A/C

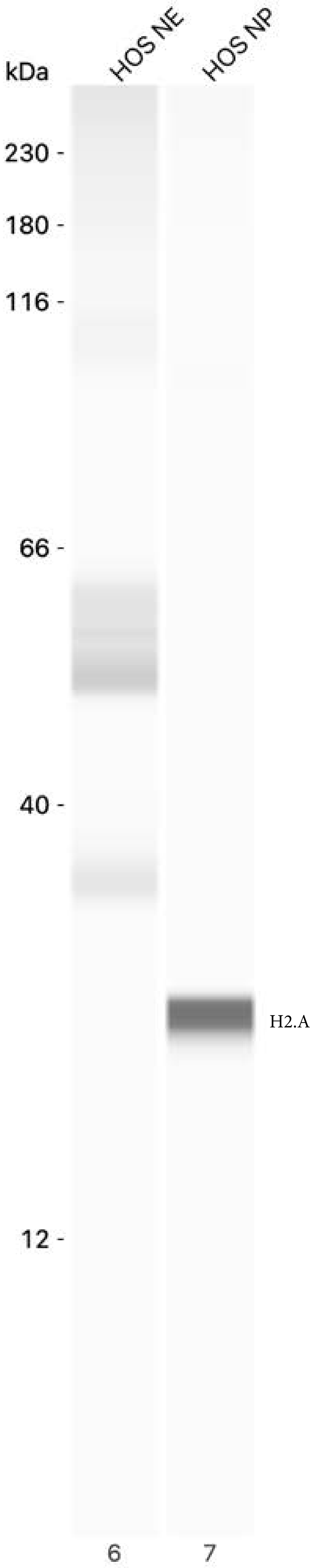
kDa
HOS NE
HOS NP
230 -
180 -
116 -
66 -
40 -
12 -
H2.A
6
7

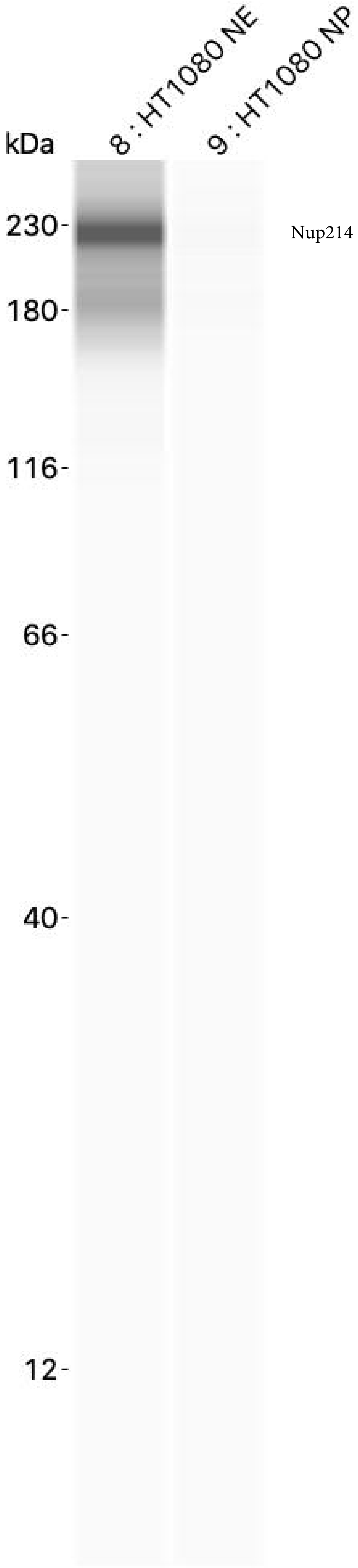

kDa
8 : HT1080 NE
9 : HT1080 NP
230
180
116
66
40
12
Nup214

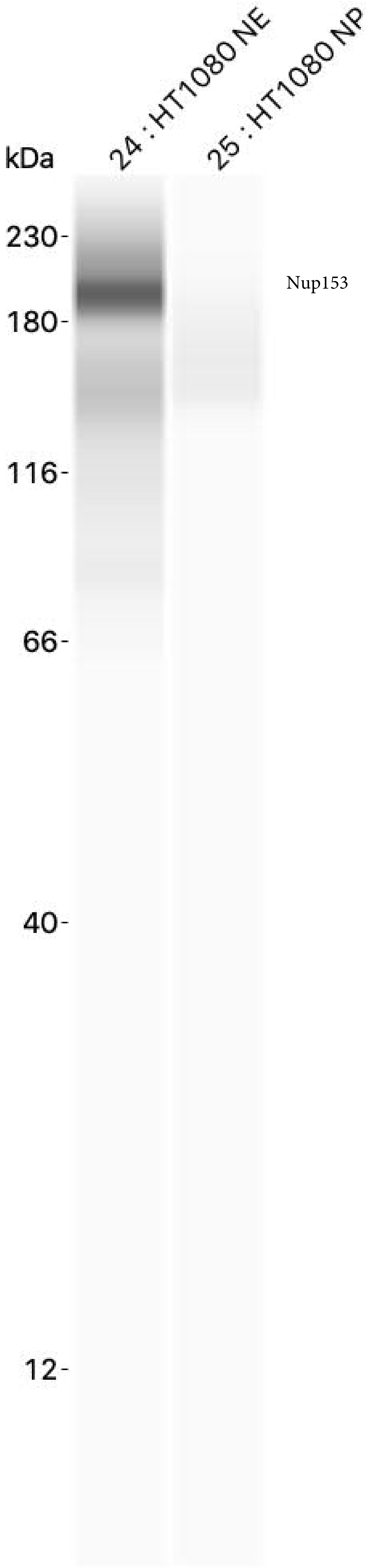
kDa
24 : HT1080 NE
25 : HT1080 NP
230
180
116
66
40
12
Nup153

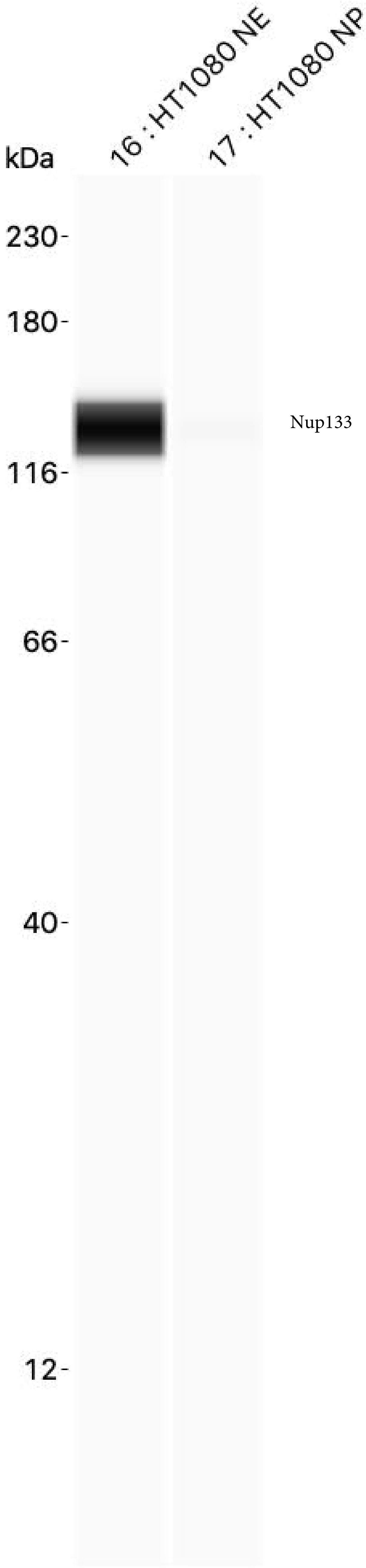
kDa
16 : HT1080 NE
17 : HT1080 NP
230-
180-
116-
66-
40-
12-
Nup133

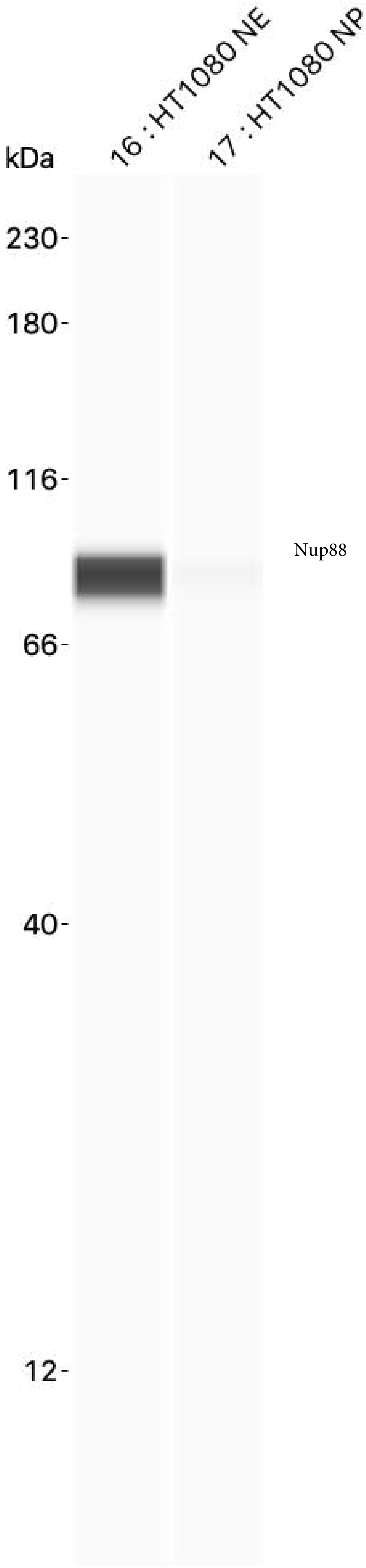
kDa
16 : HT1080 NE
17 : HT1080 NP
230
180
116
66
40
12
Nup88

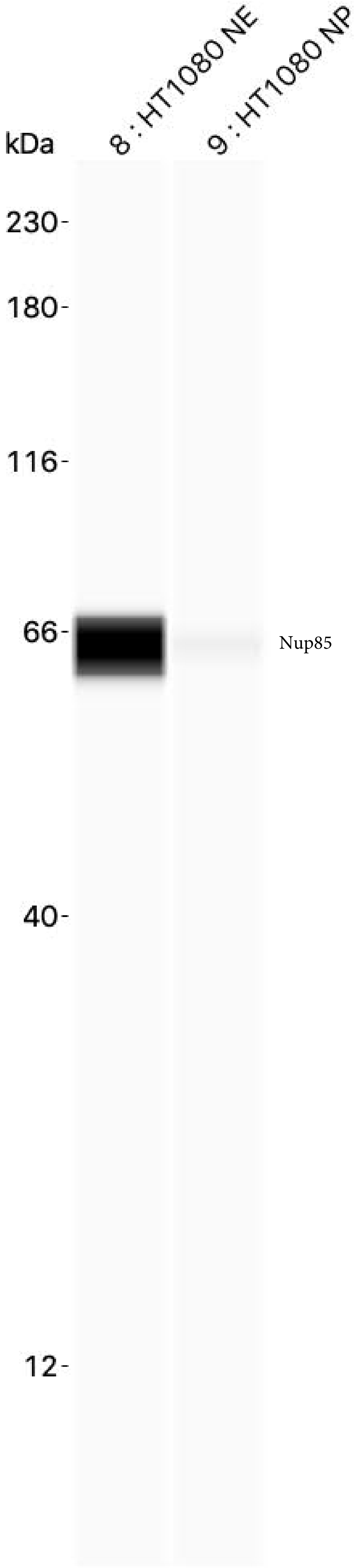
kDa
8 : HT1080 NE
9 : HT1080 NP
230
180
116
66
40
12
Nup85

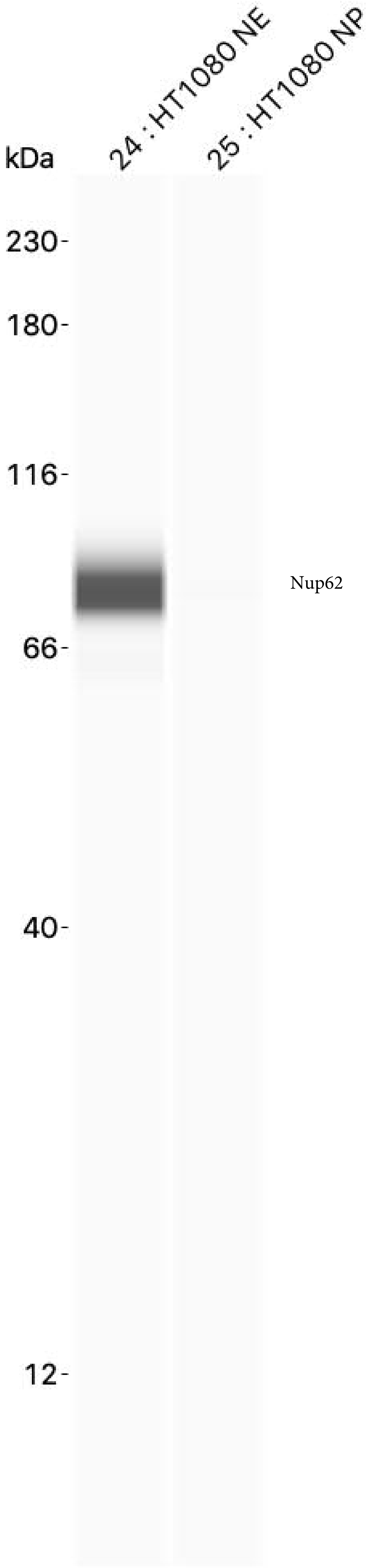
24 : HT1080 NE
25 : HT1080 NP
kDa
230
180
116
66
40
12
Nup62

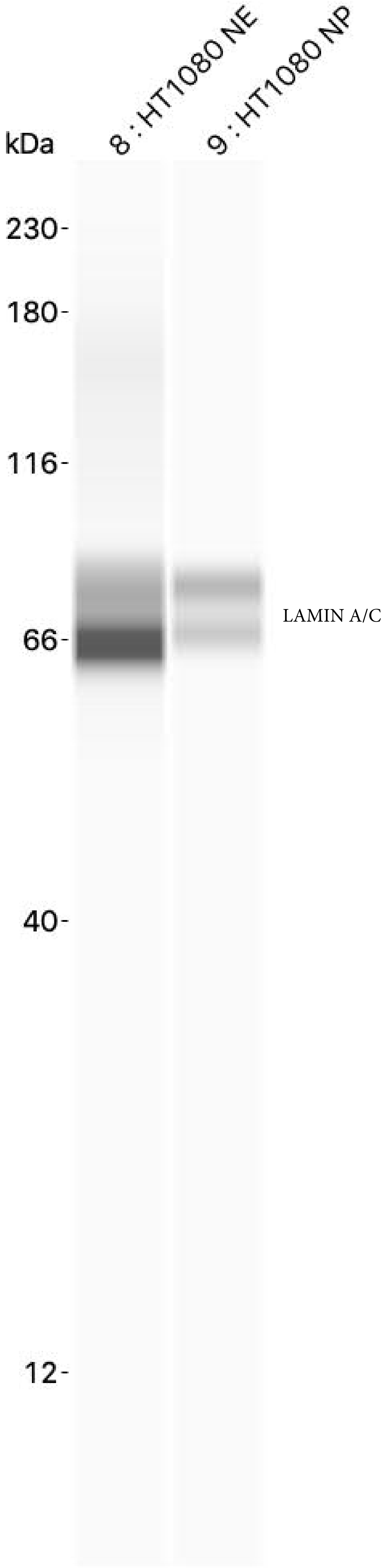
kDa
8 : HT1080 NE
9 : HT1080 NP
230
180
116
66
40
12
LAMIN A/C

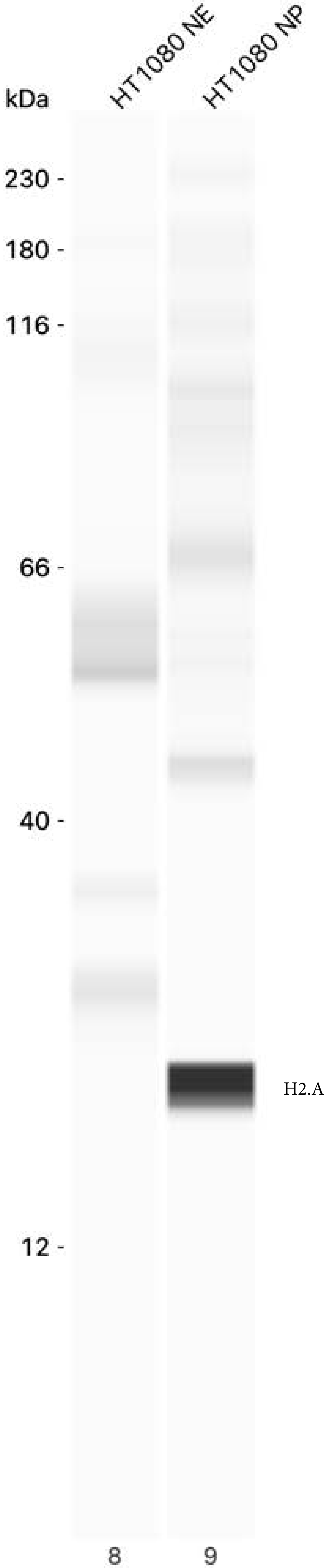
kDa
HT1080 NE
HT1080 NP
230 -
180 -
116 -
66 -
40 -
12 -
H2.A
8
9
